# Supplementary material for: Novel Bis- and Mono-Pyrrolo[2,3-d]pyrimidine and Purine Derivatives: Synthesis, Computational Analysis and Antiproliferative Evaluation
Source: Molecules. 2021 Jun 1;26(11):3334. doi: 10.3390/molecules26113334 (PMC8199500; doi:10.3390/molecules26113334)
Supplement: Supplementary file 1 [file molecules-26-03334-s001.zip › molecules-1232551-supplementary.pdf]

Supporting Information for:

Novel 6-substituted bis- and mono-pyrrolo[2,3-*d*]pyrimidine and purine derivatives: synthesis, computational analysis and antiproliferative evaluation

Andrea Bistrović Popov[1,2], Robert Vianelo[3], Petra Grbčić[4], Mirela Sedić[4], Sandra Kraljević Pavelić[5], Krešimir Pavelić[6], Silvana Raić-Malić[1,*]

[1] Department of Organic Chemistry, Faculty of Chemical Engineering and Technology, University of Zagreb, Marulićev trg 20, HR-10000 Zagreb, Croatia, e-mail: sraic@fkit.hr (SRM)

[2] Department of Chemical Engineering and Biotechnology, University of Cambridge, Philippa Fawcet Drive, CB30AS, Cambridge, UK, e-mail: ab2602@cam.ac.uk (ABP)

[3] Division of Organic Chemistry and Biochemistry, Ruđer Bošković Institute, Bijenička 54, HR-10000 Zagreb, Croatia, e-mail: robert.vianello@irb.hr (RV)

[4] Department of Biotechnology, University of Rijeka, Ulica Radmile Matejčić 2, HR-51000 Rijeka, Croatia, e-mail: petra.grbcic@ biotech.uniri.hr (PG), msedic@biotech.uniri.hr (MS)

[5] University of Rijeka, Faculty of Health Studies, Ulica Viktora Cara Emina 5, 51000 Rijeka, Croatia, sandrakp@uniri.hr (SKP)

[6] Faculty of Medicine, Juraj Dobrila University of Pula, HR-52100, Pula, Croatia, e-mail: pavelic@unipu.hr (KP)

Content	Pages
1. ^{1}H and ^{13}C NMR spectra of novel compounds	S2–S40
2. Computational analysis	S41–S47

1. ^{1}H and ^{13}C NMR spectra of novel compounds

Fig. S1 a) ^{1}H NMR and b) ^{13}C NMR of compd. **2b**

a)

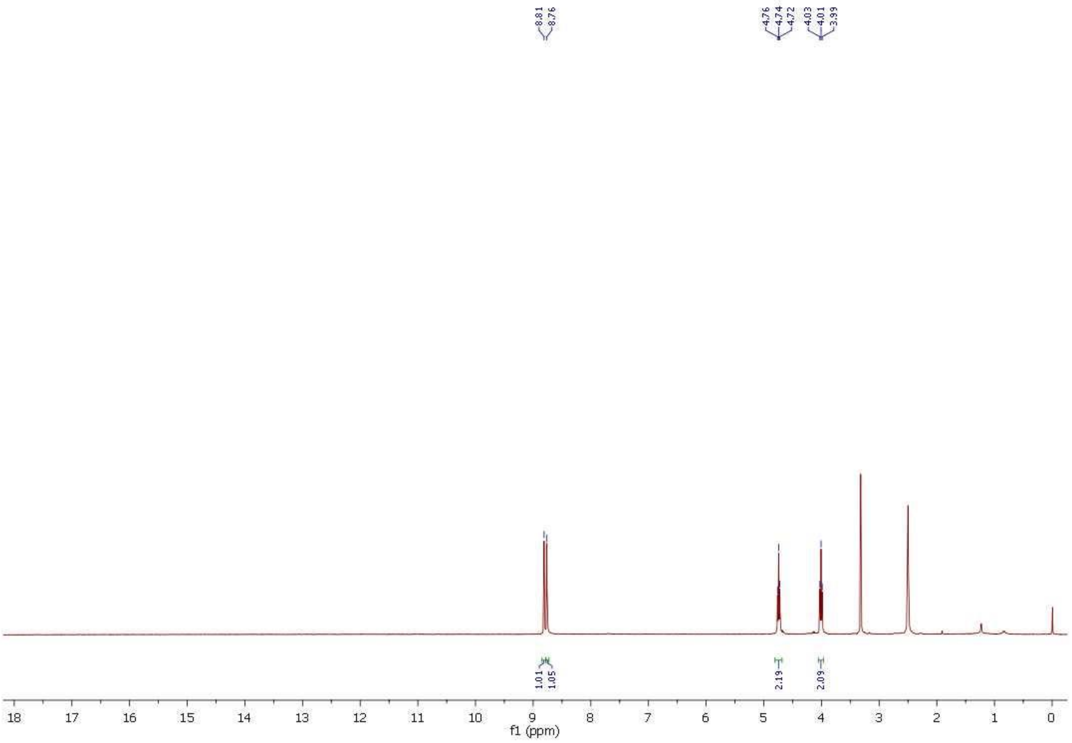

b)

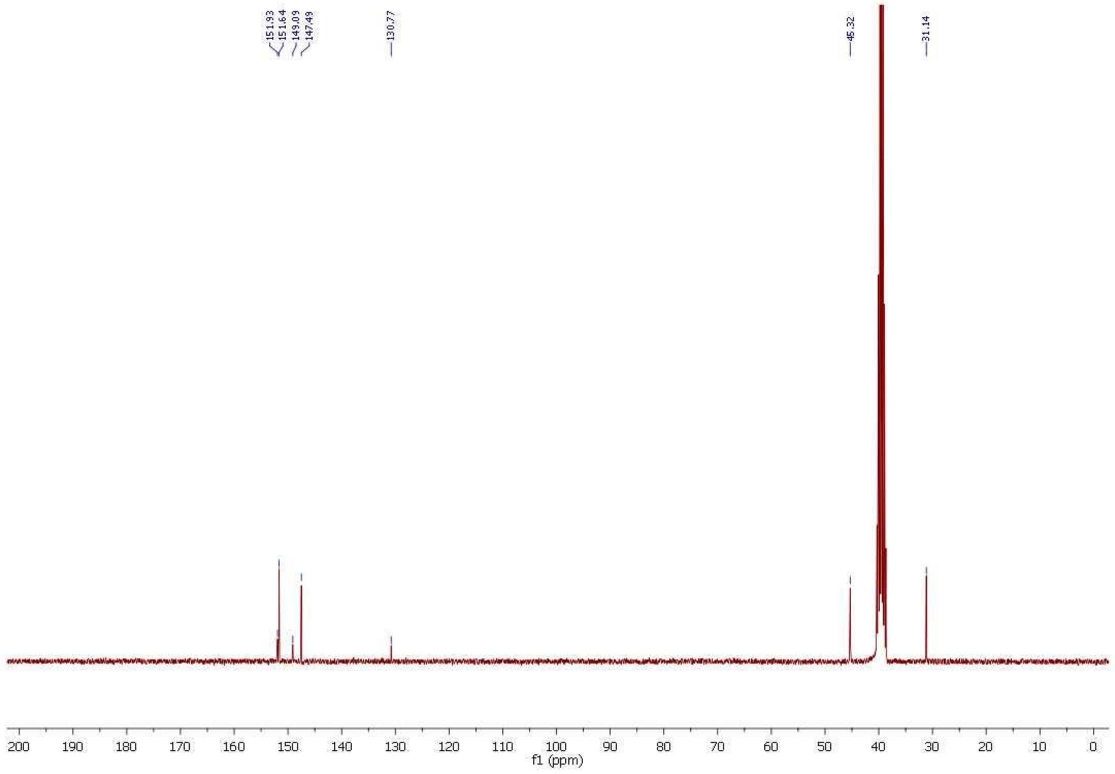

Fig. S2 a) ^{1}H NMR and b) ^{13}C NMR of compd. **2c**

a)

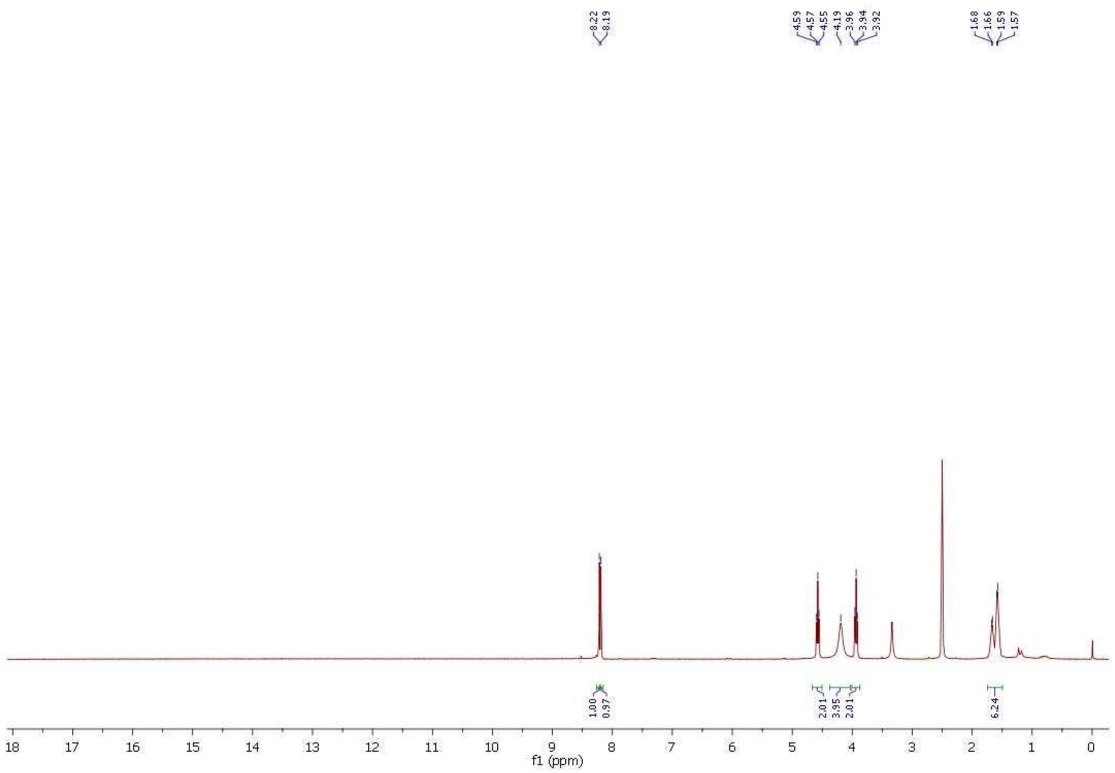

b)

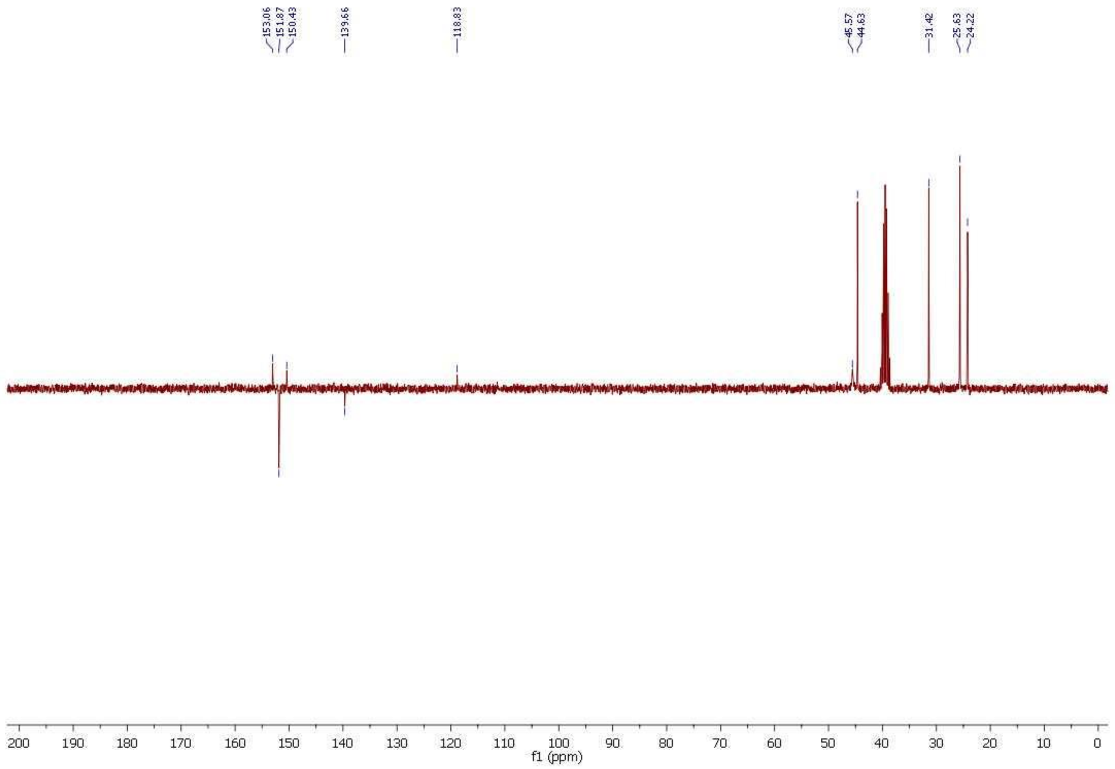

Fig. S3 a) ^{1}H NMR and b) ^{13}C NMR of compd. **2d**

a)

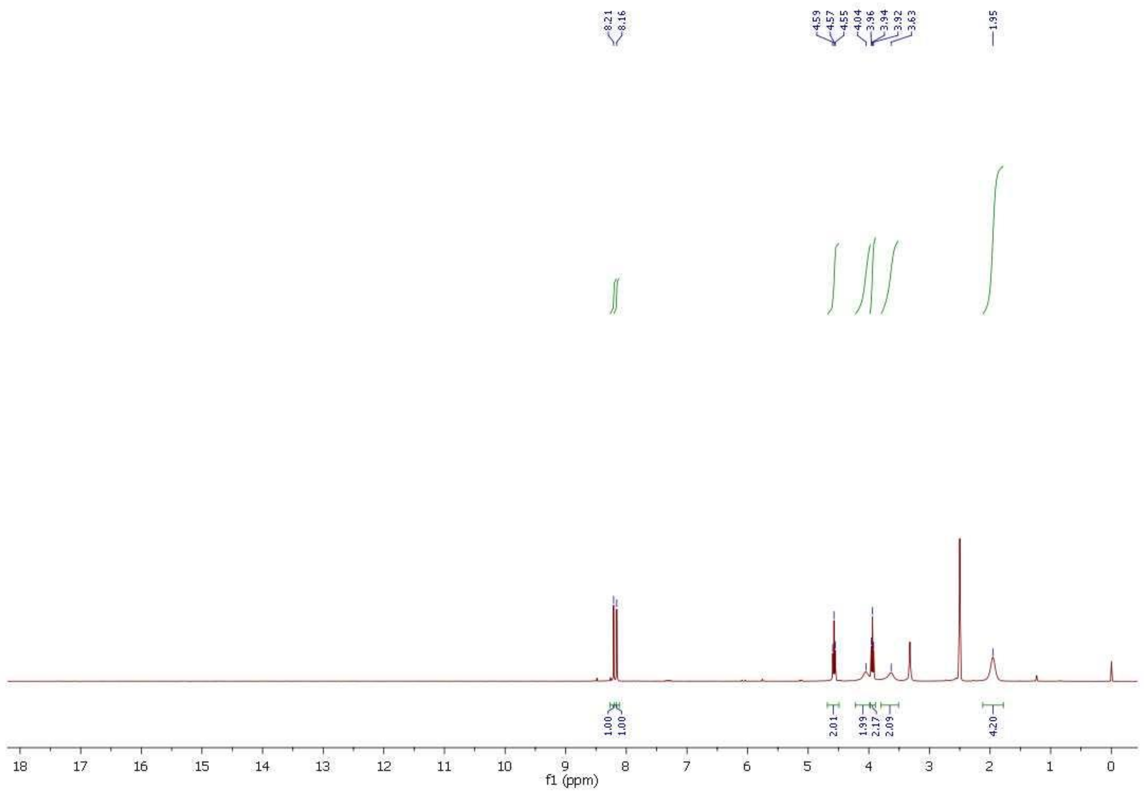

b)

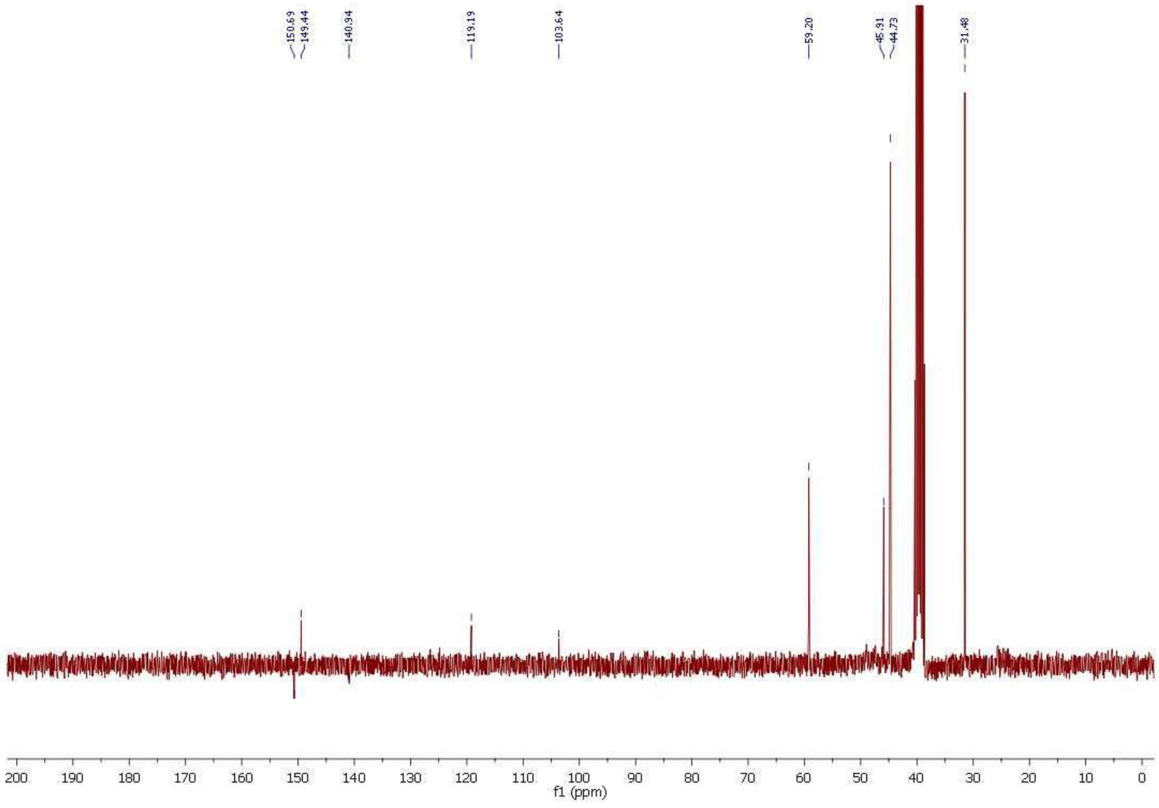

Fig. S4 a) ^{1}H NMR and b) ^{13}C NMR of compd. **3b**

a)

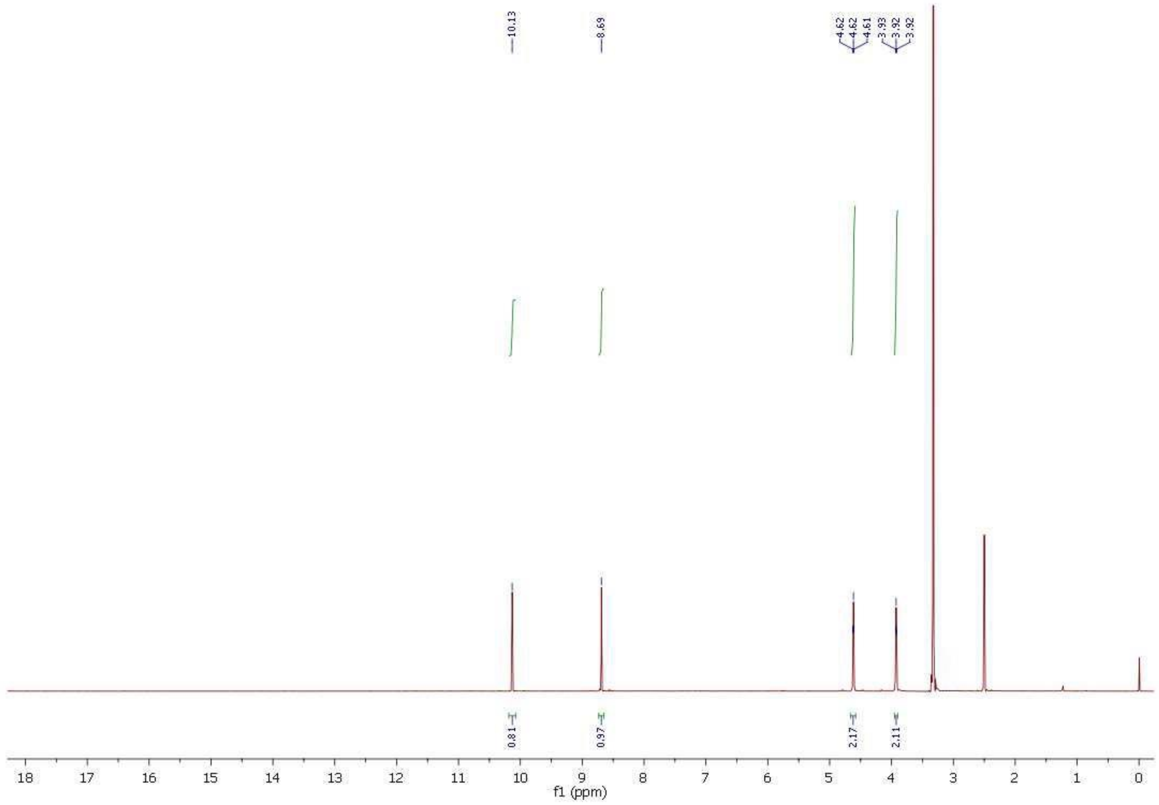

b)

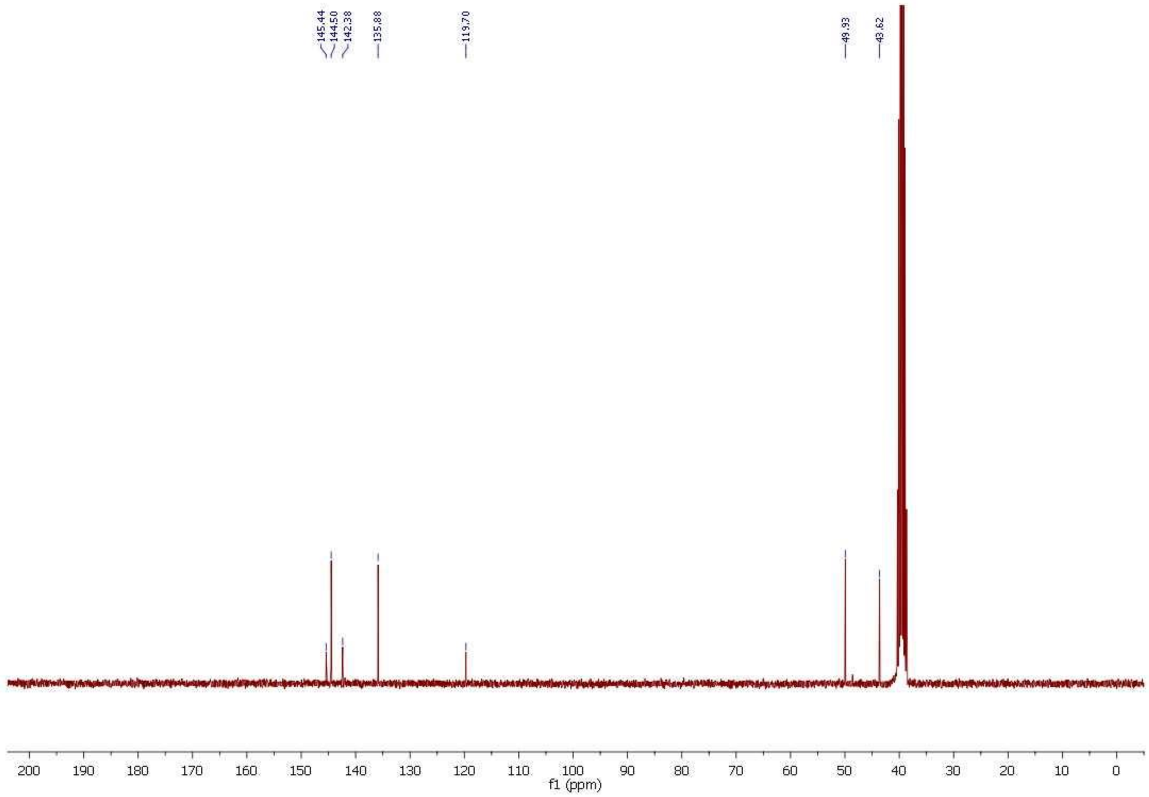

Fig. S5 a) ^{1}H NMR and b) ^{13}C NMR of compd. **3c**

a)

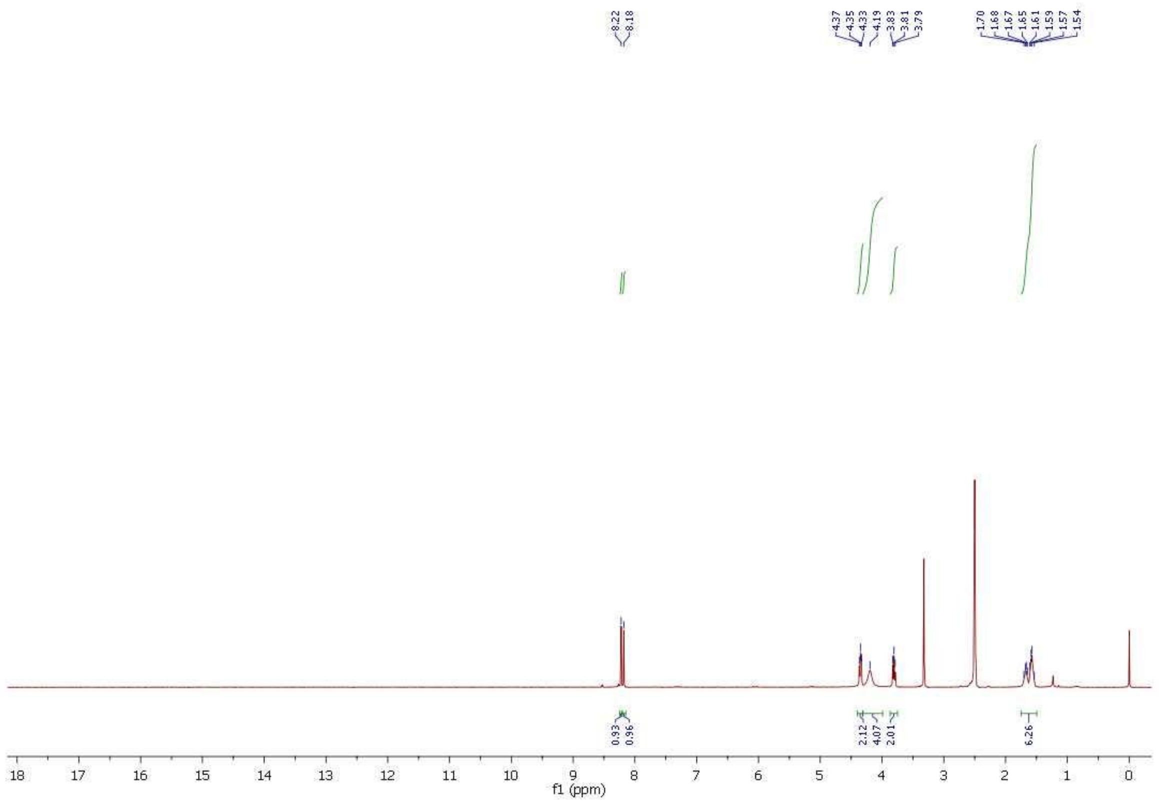

b)

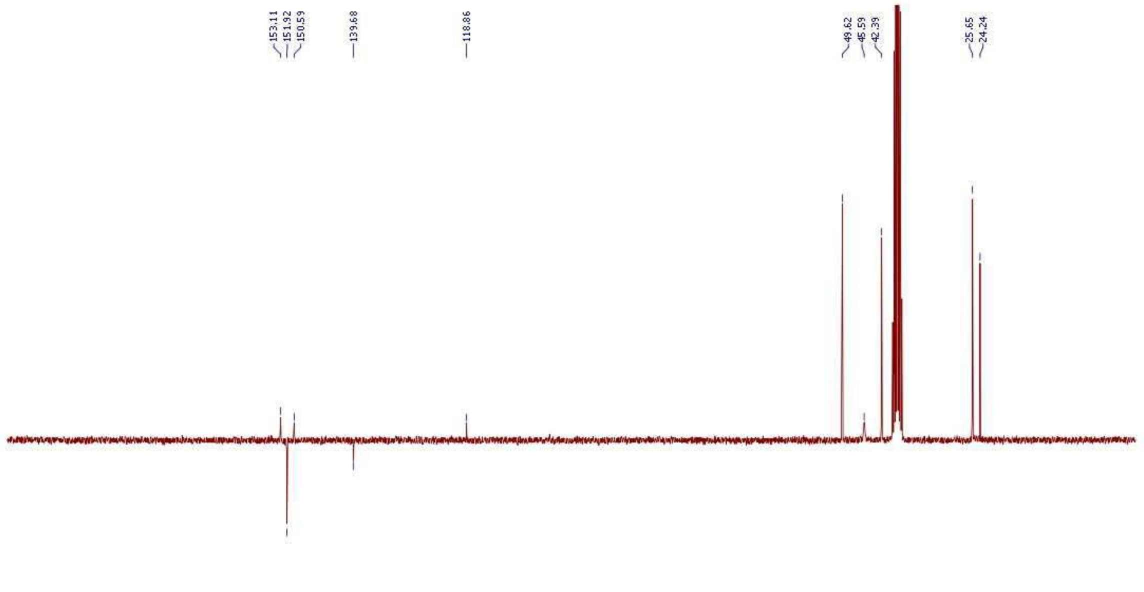

Fig. S6 a) ^{1}H NMR and b) ^{13}C NMR of compd. **3d**

a)

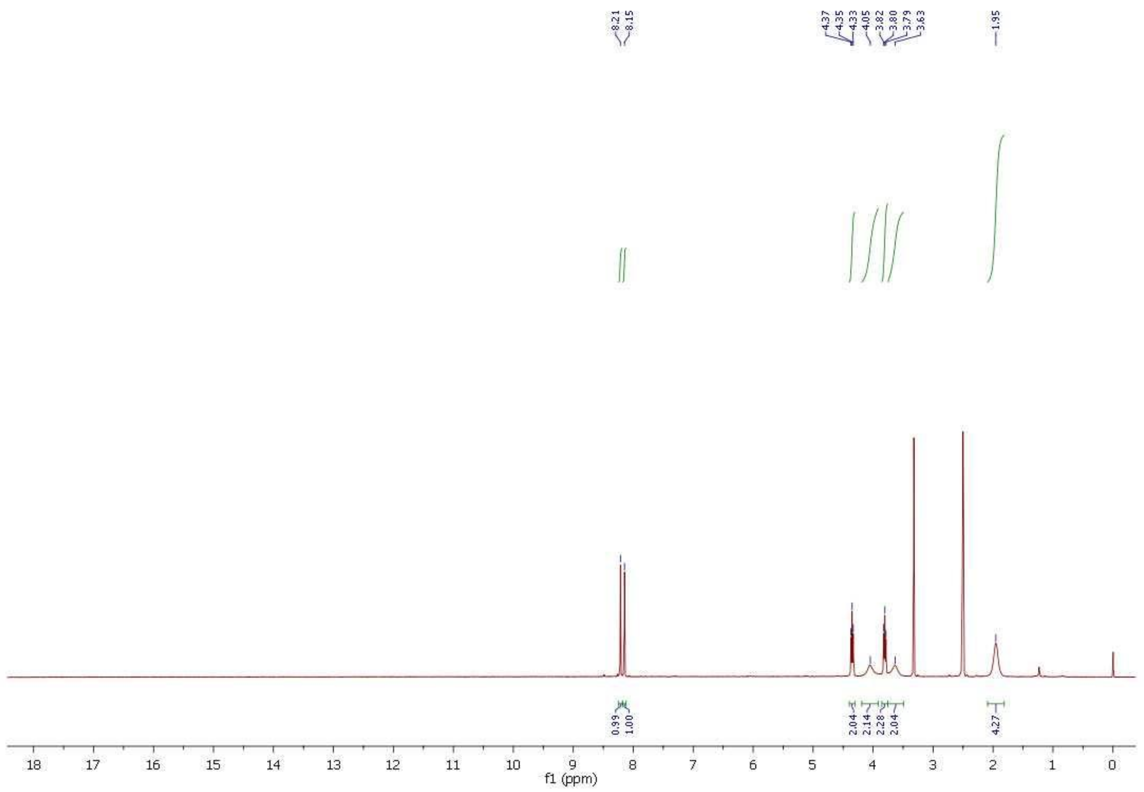

b)

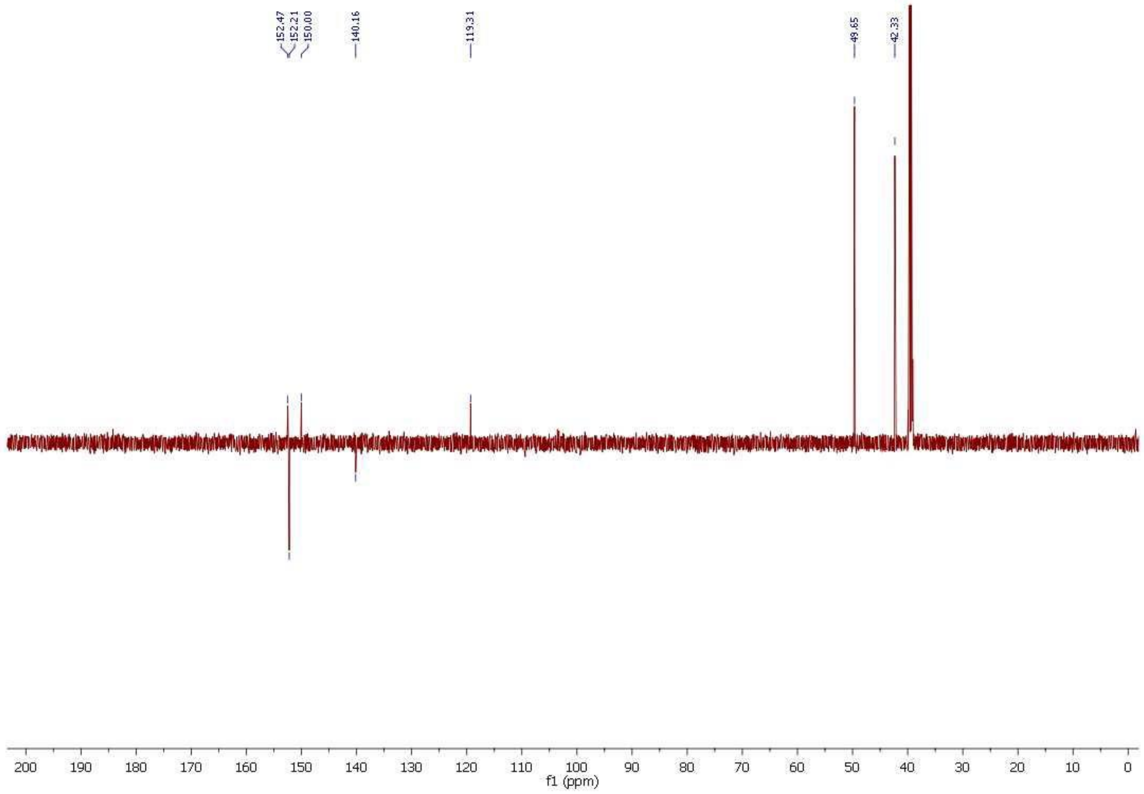

Fig. S7 a) ^{1}H NMR and b) ^{13}C NMR of compd. **3f**

a)

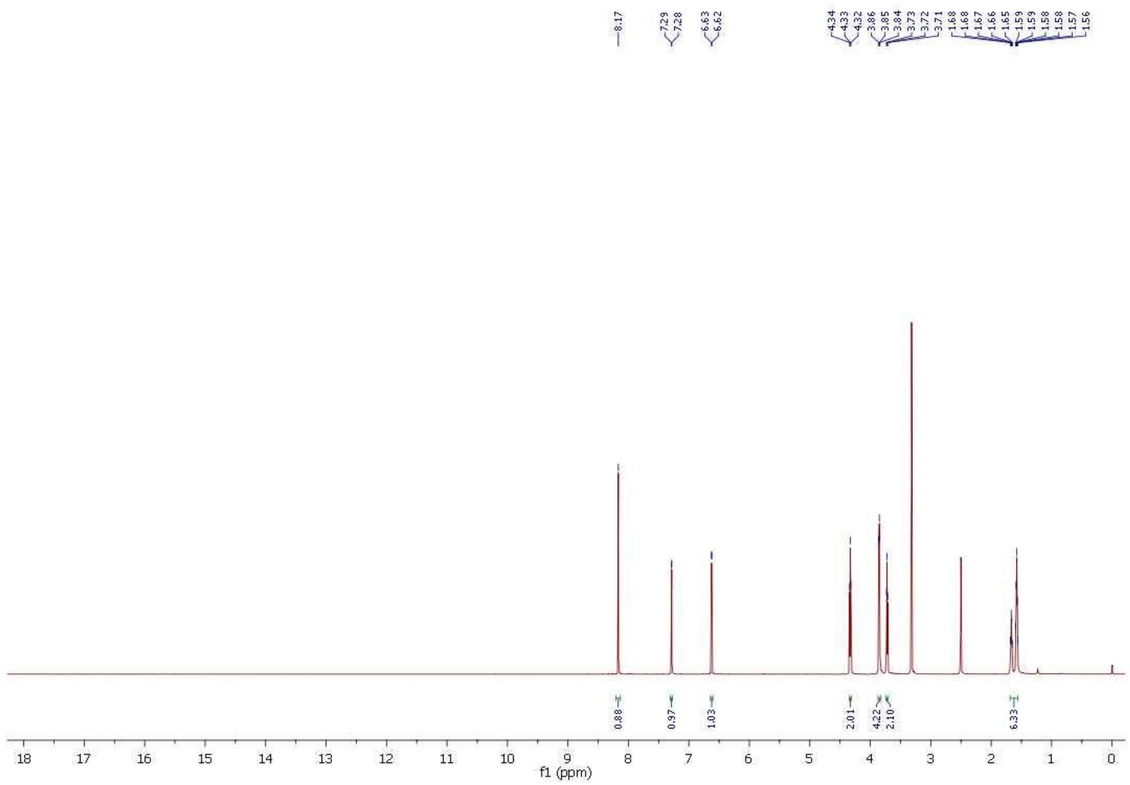

b)

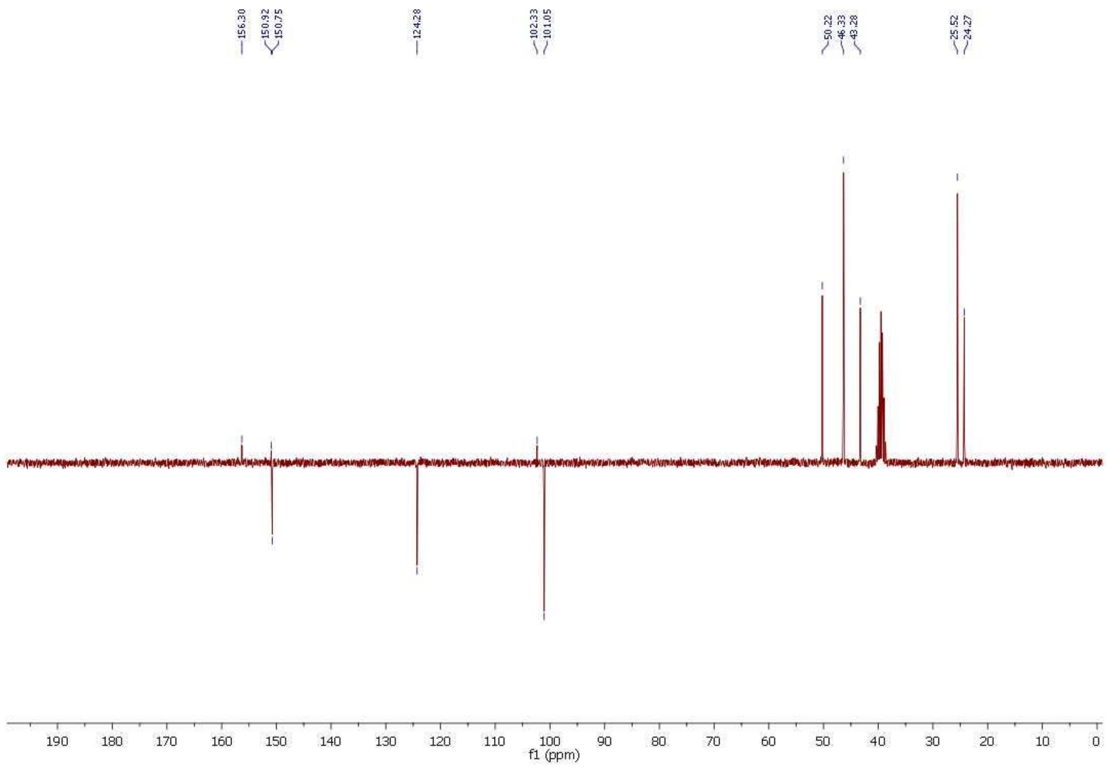

Fig. S8 a) ^{1}H NMR and b) ^{13}C NMR of compd. **5a**

a)

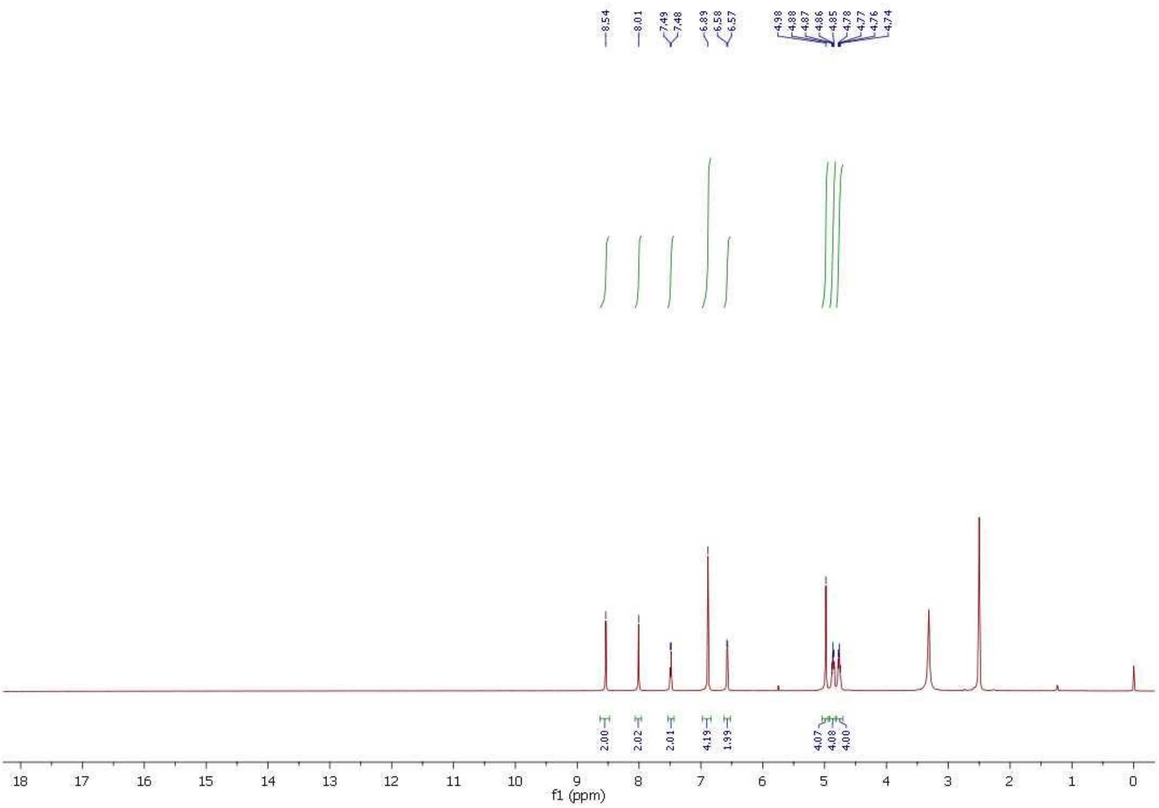

b)

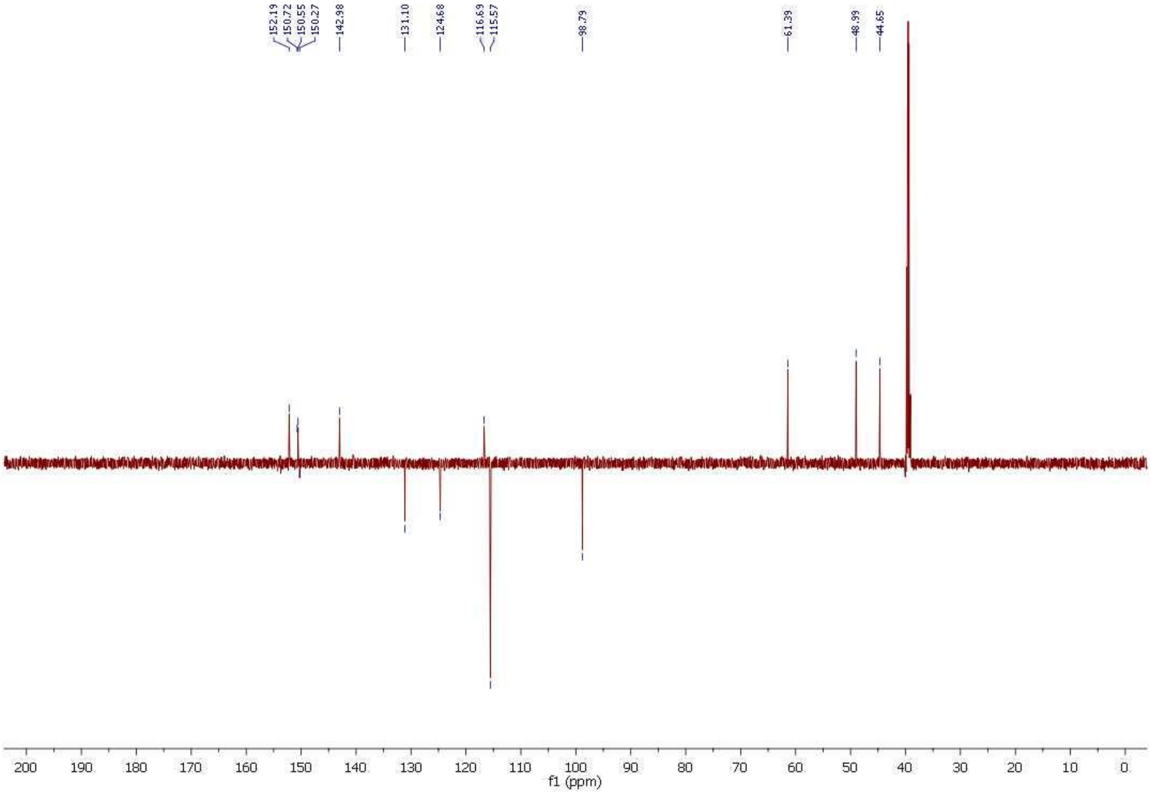

Fig. S9 a) ^{1}H NMR and b) ^{13}C NMR of compd. **5b**

a)

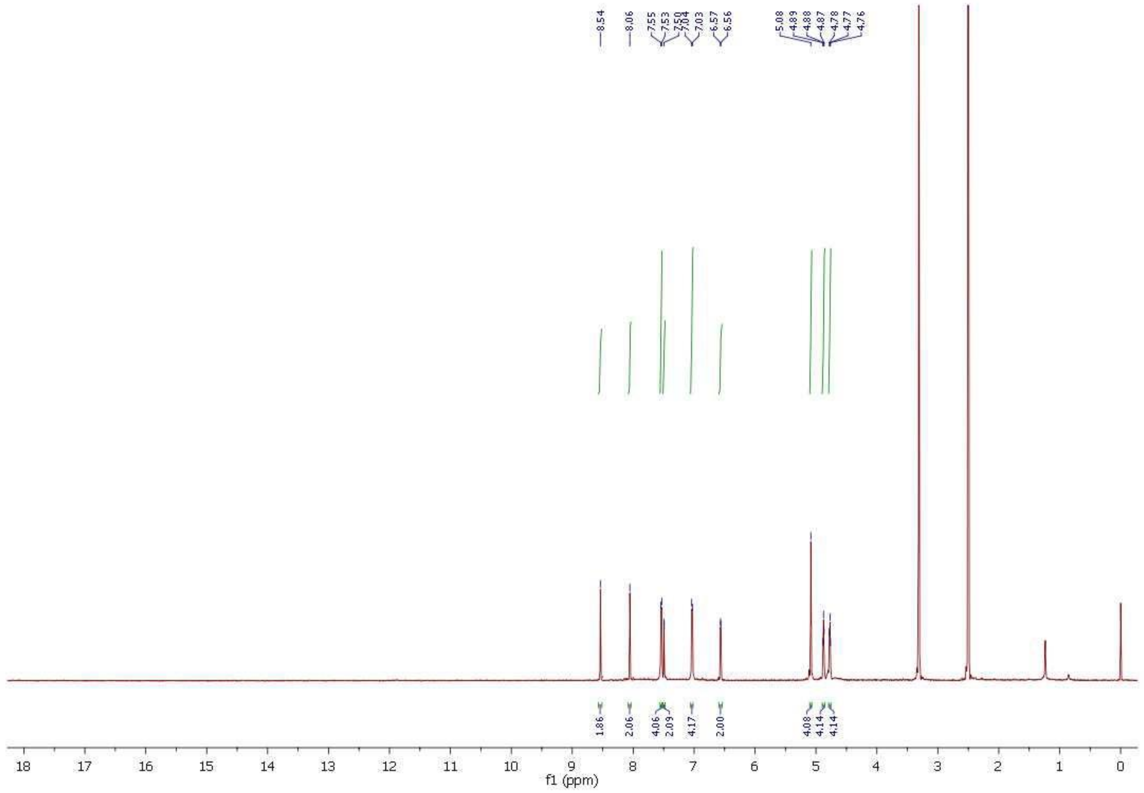

b)

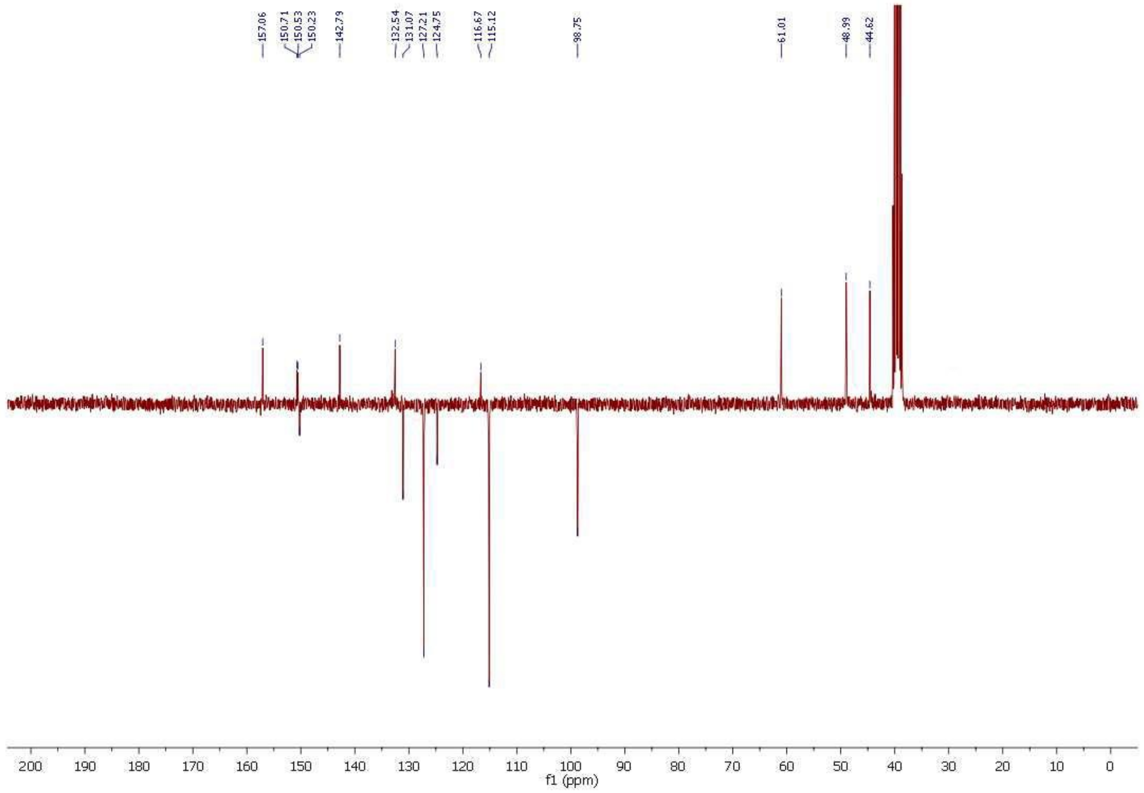

Fig. S10 a) ^{1}H NMR and b) ^{13}C NMR of compd. **5c**

a)

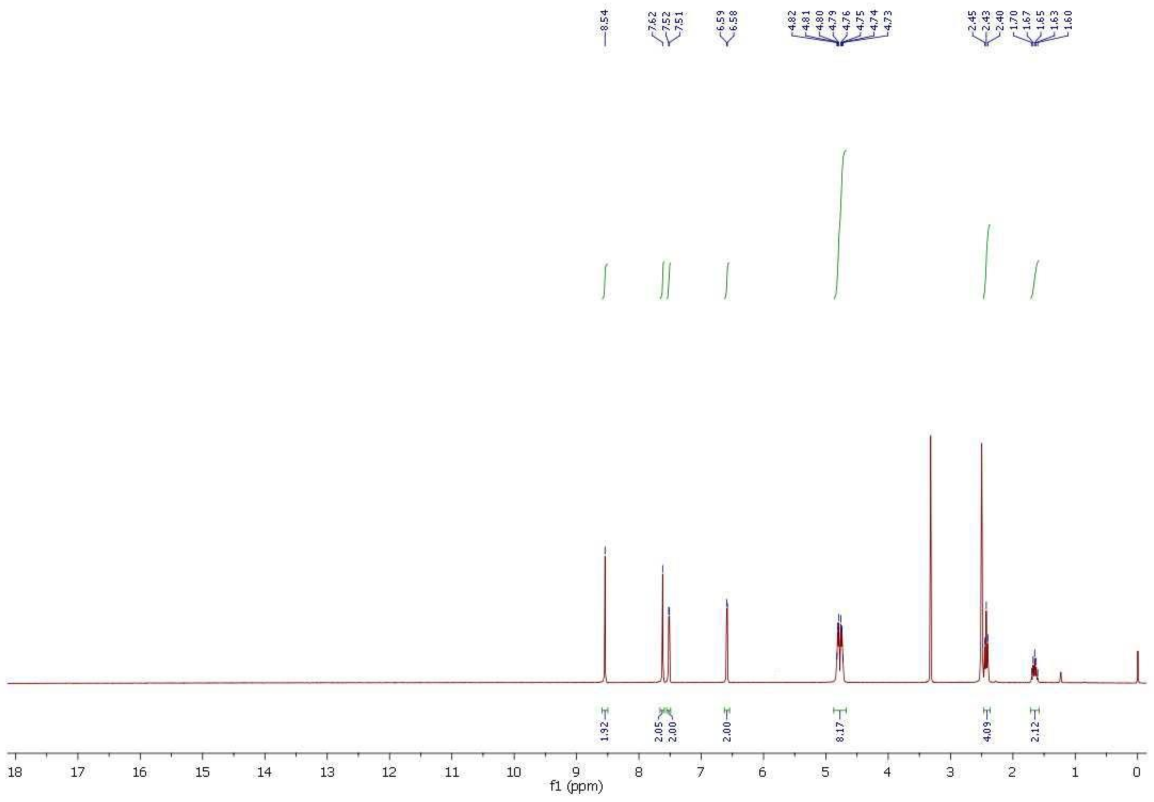

b)

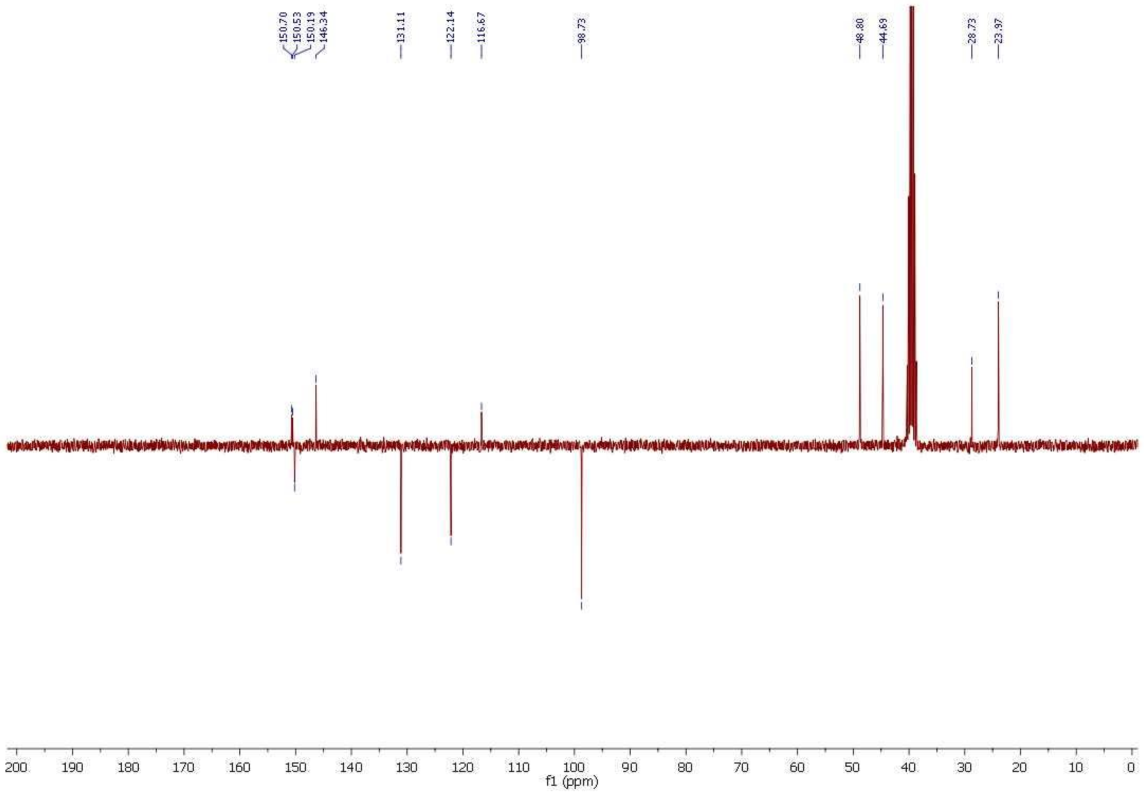

Fig. S11 a) ^{1}H NMR and b) ^{13}C NMR of compd. **5d**

a)

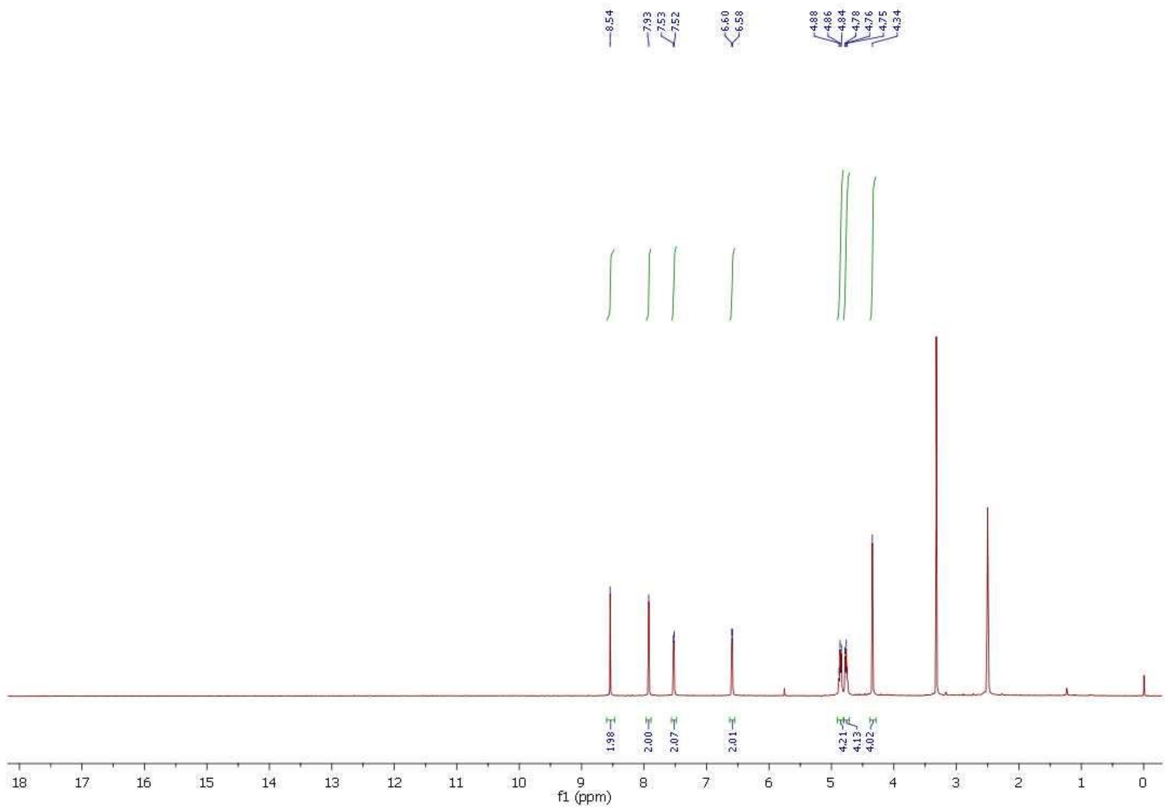

b)

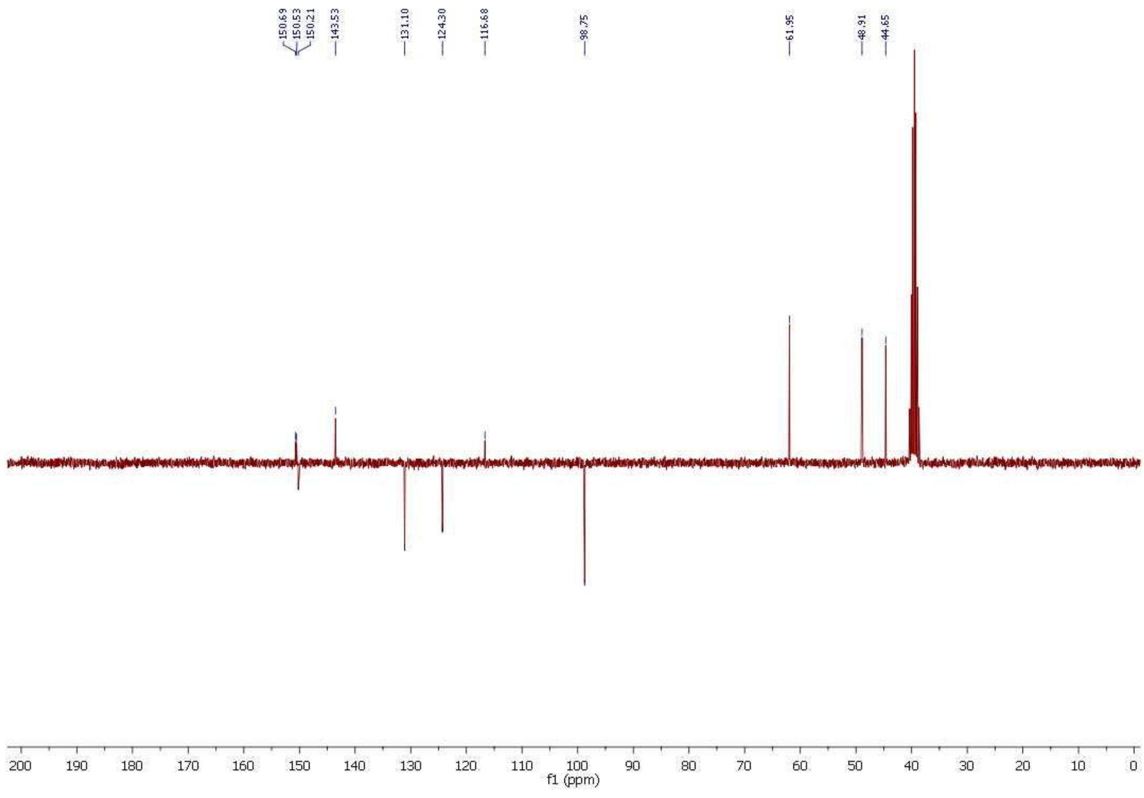

Fig. S12 a) ^{1}H NMR and b) ^{13}C NMR of compd. **5f**

a)

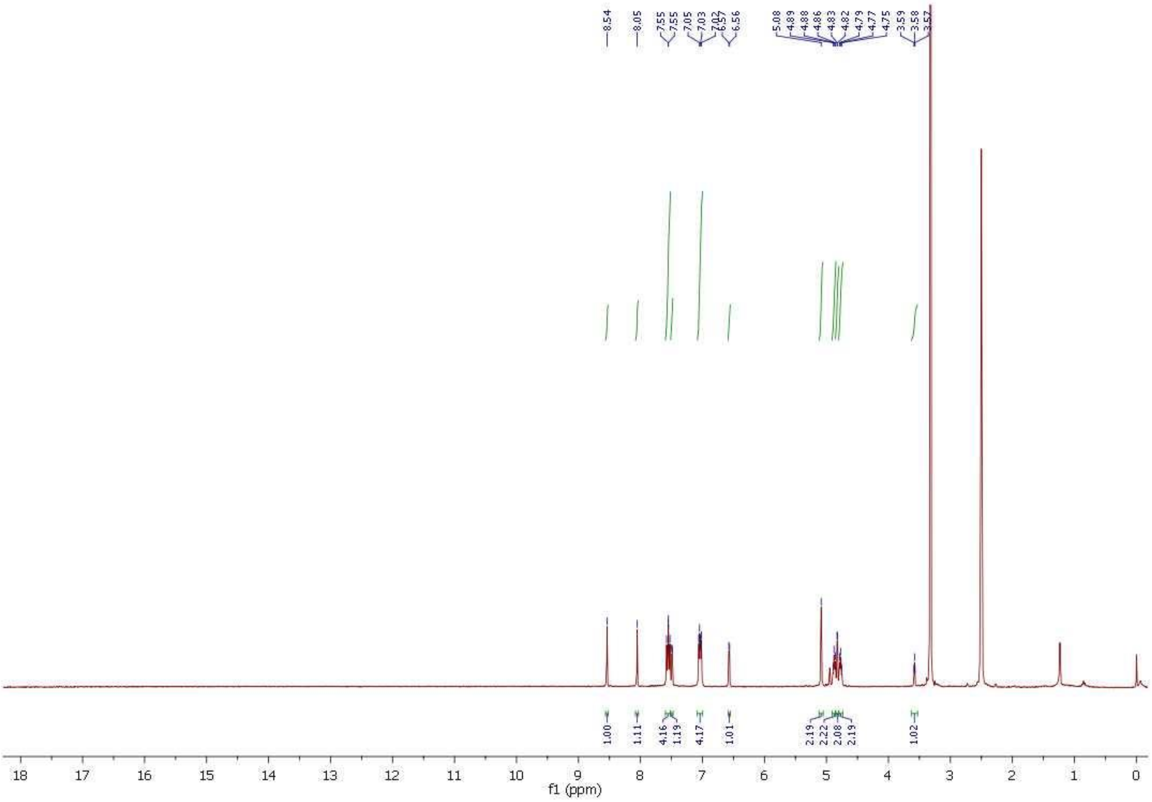

b)

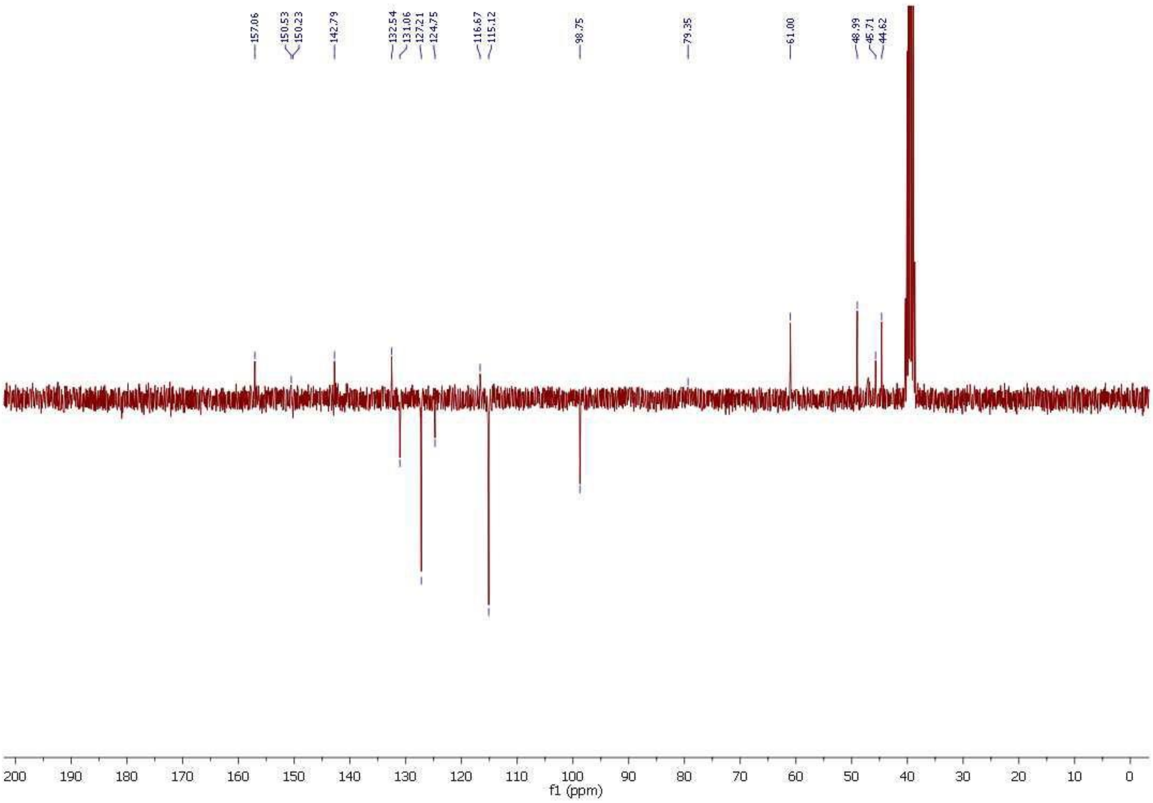

Fig. S13 a) ^{1}H NMR and b) ^{13}C NMR of compd. **5g**

a)

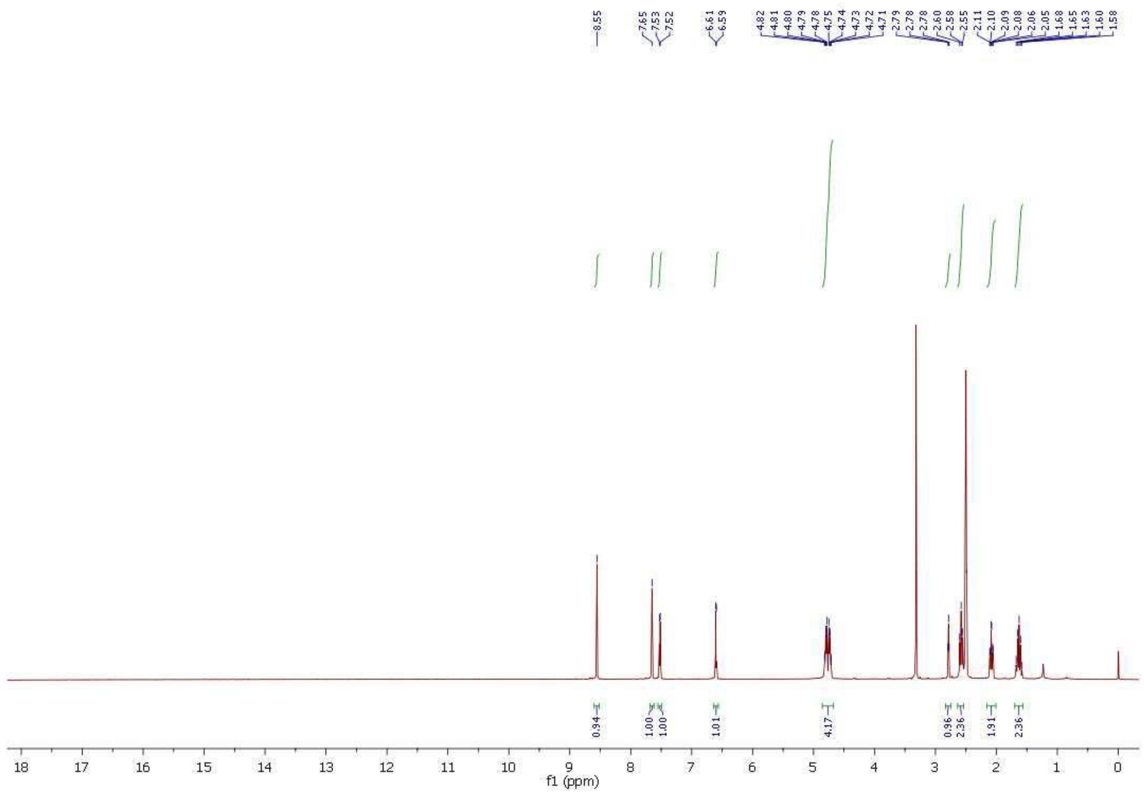

b)

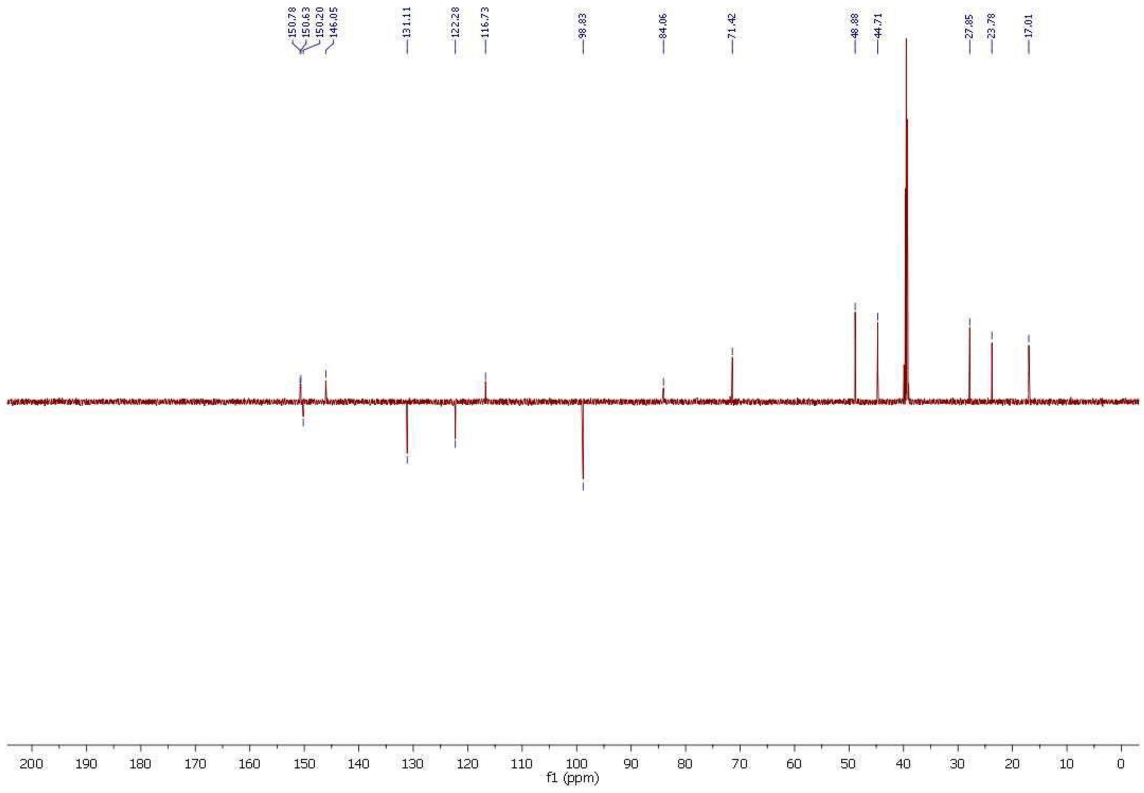

Fig. S14 a) ^{1}H NMR and b) ^{13}C NMR of compd. **6a**

a)

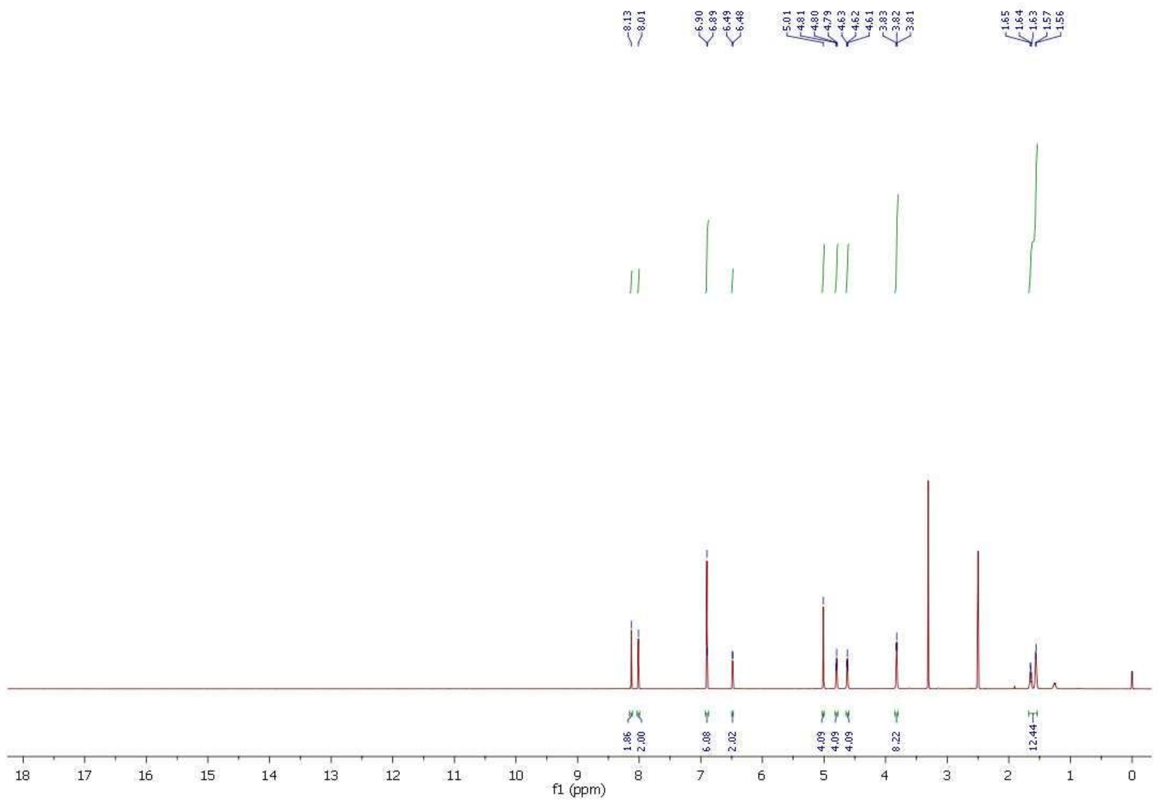

b)

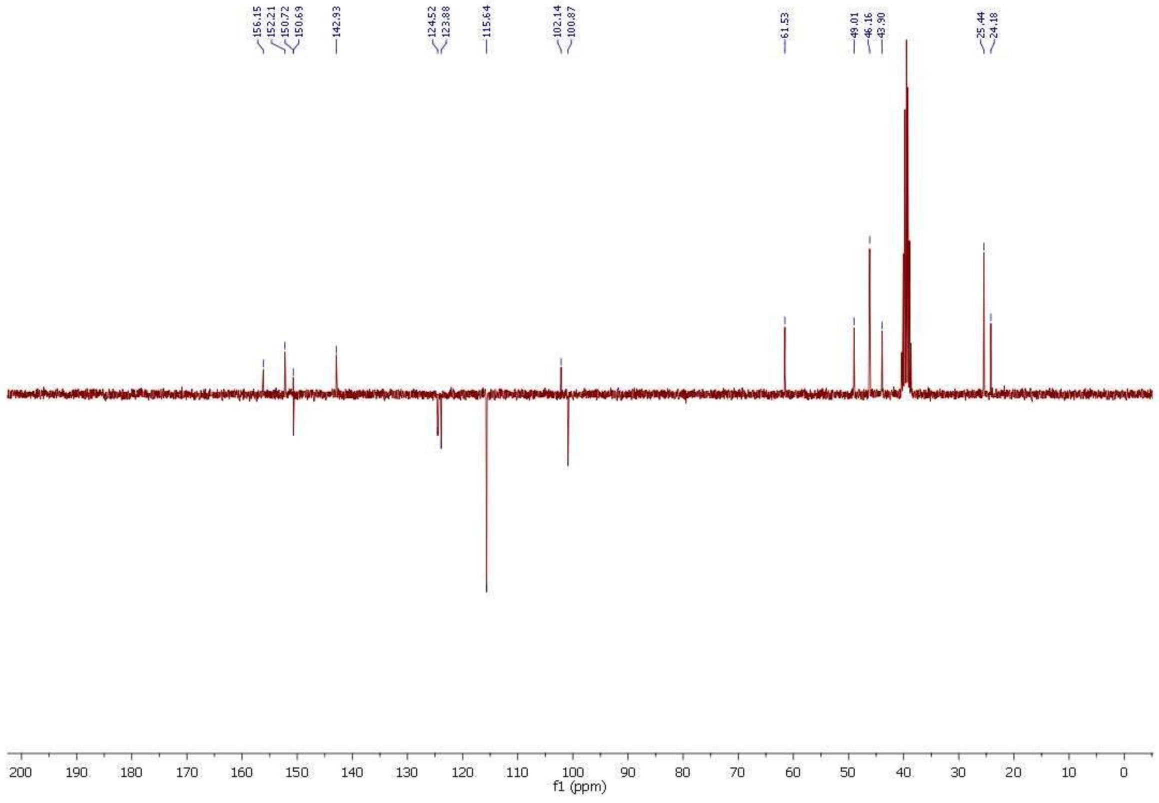

Fig. S15 a) ^{1}H NMR and b) ^{13}C NMR of compd. **6b**

a)

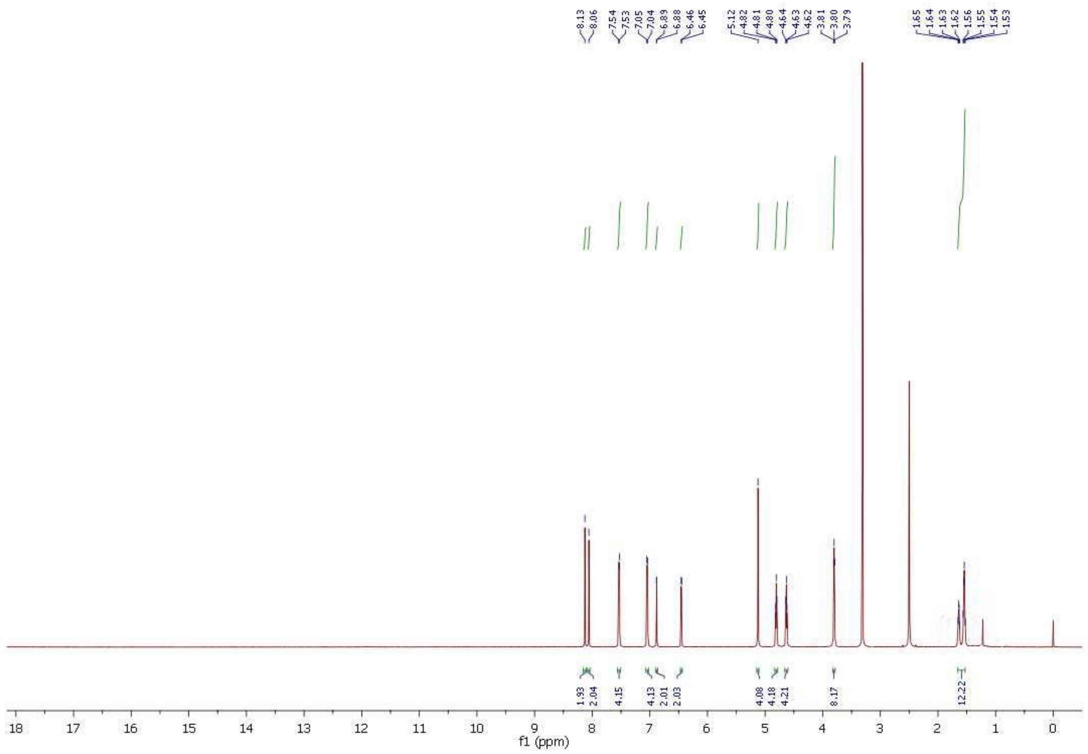

b)

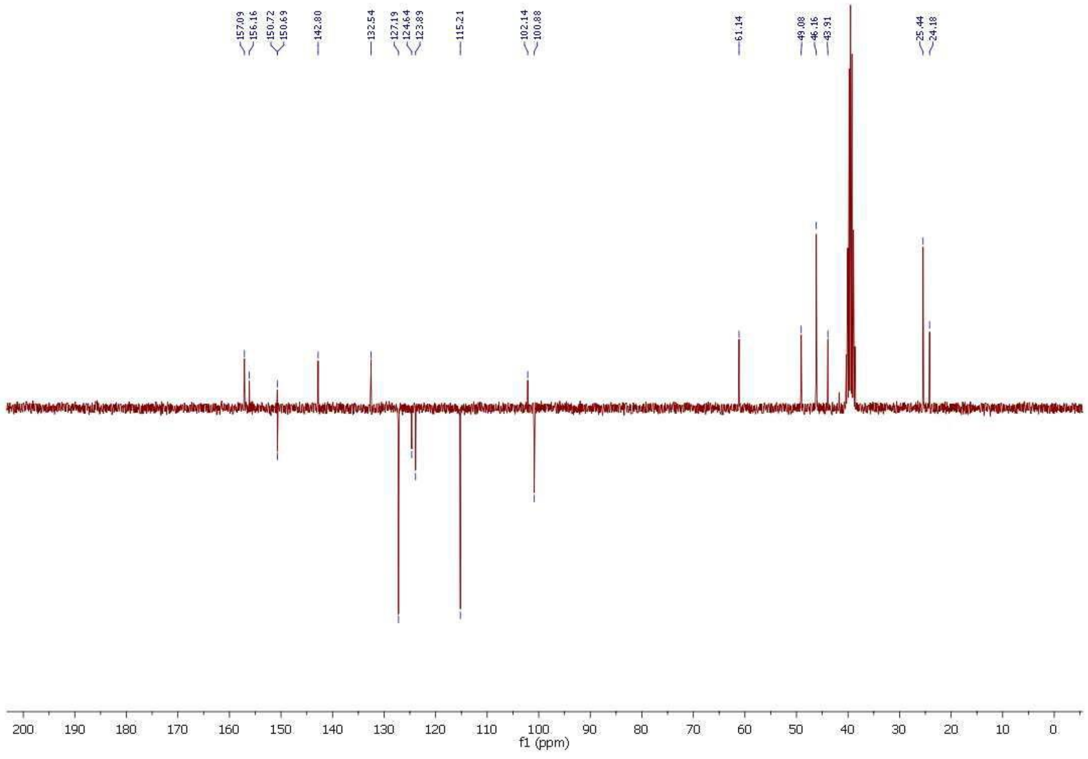

Fig. S16 a) ^{1}H NMR and b) ^{13}C NMR of compd. **6c**

a)

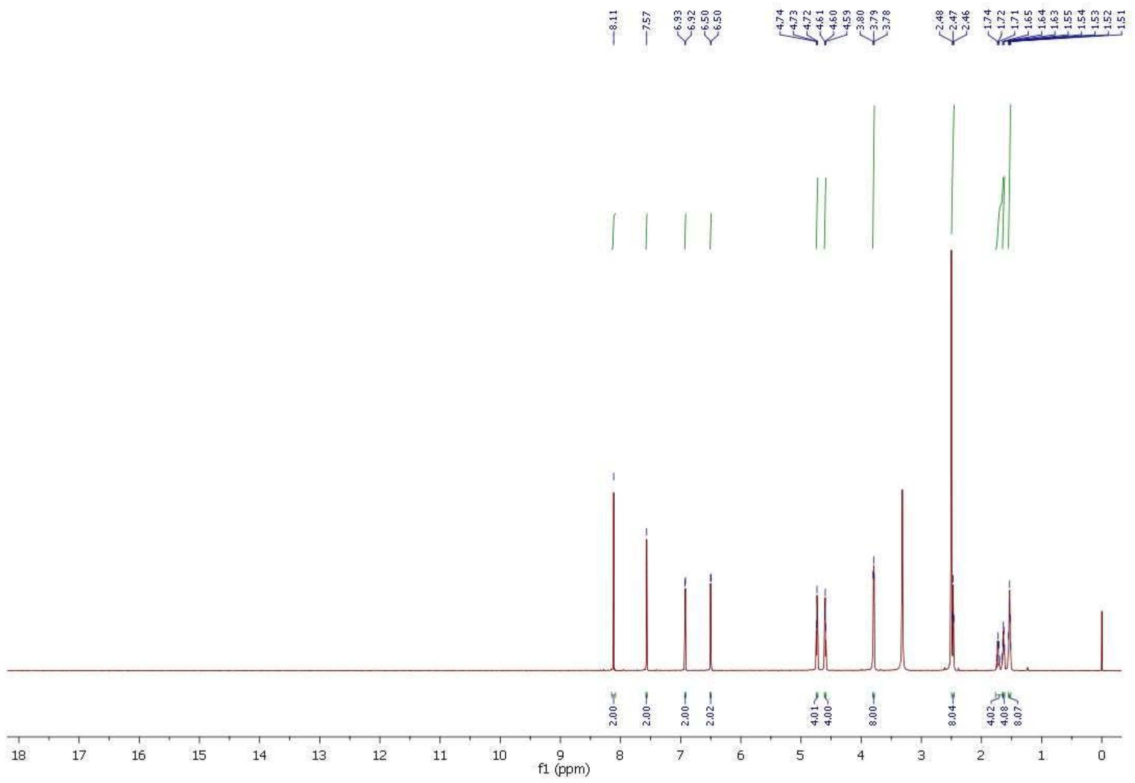

b)

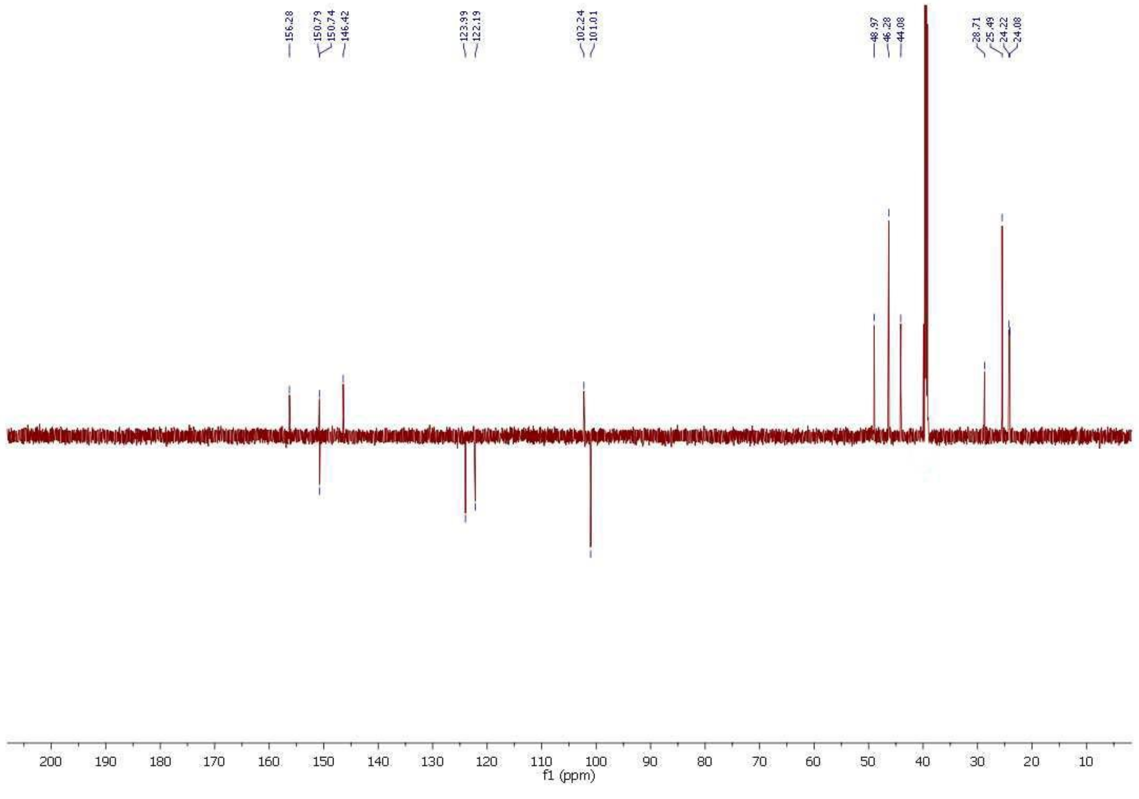

Fig. S17 a) ^{1}H NMR and b) ^{13}C NMR of compd. **6d**

a)

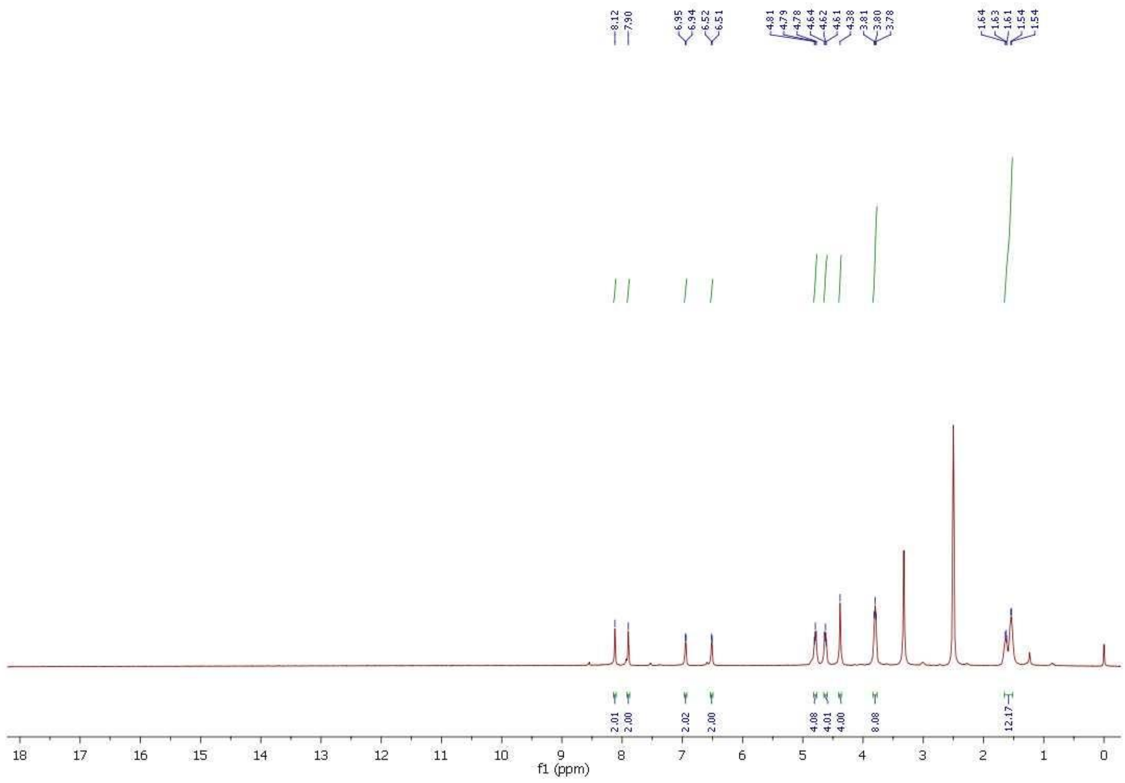

b)

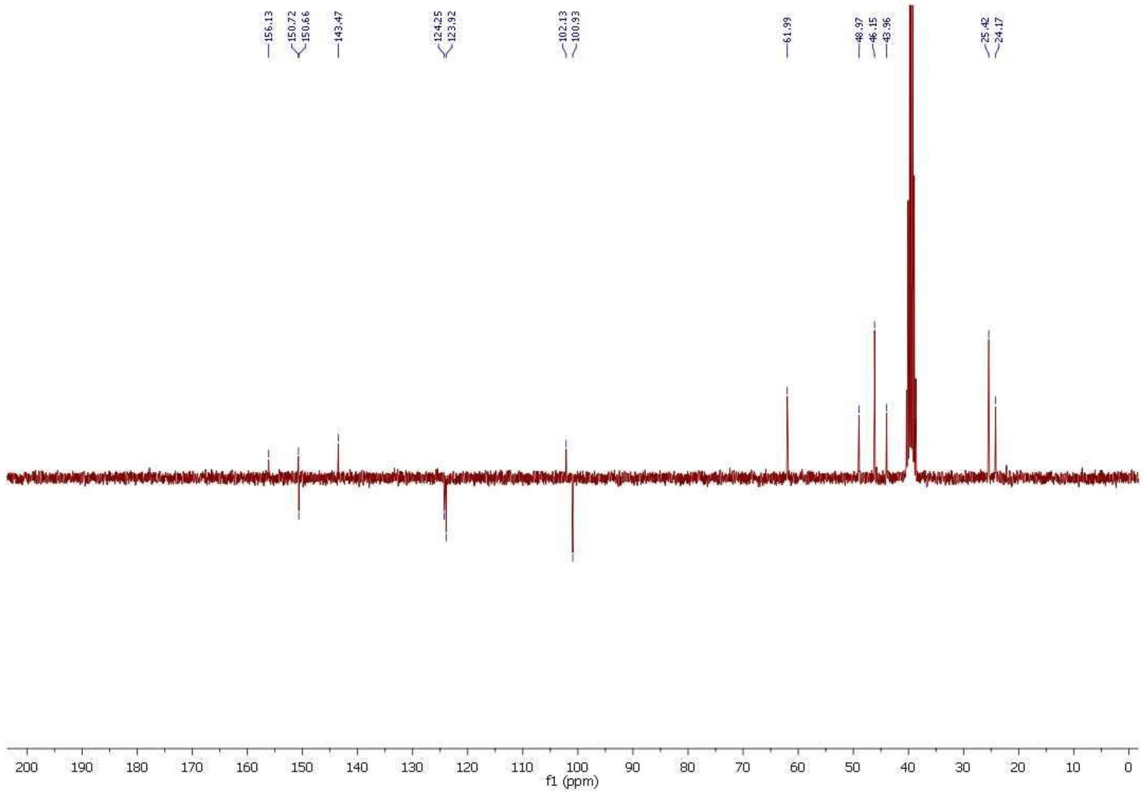

Fig. S18 a) ^{1}H NMR and b) ^{13}C NMR of compd. **7a**

a)

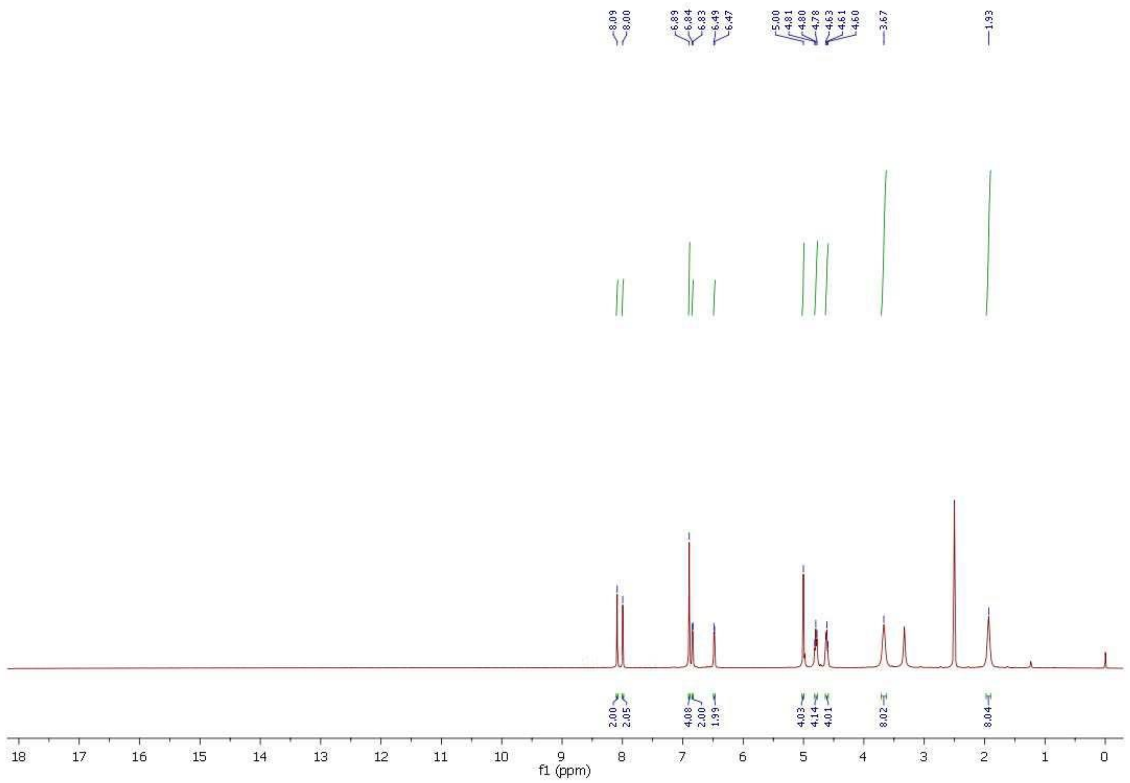

b)

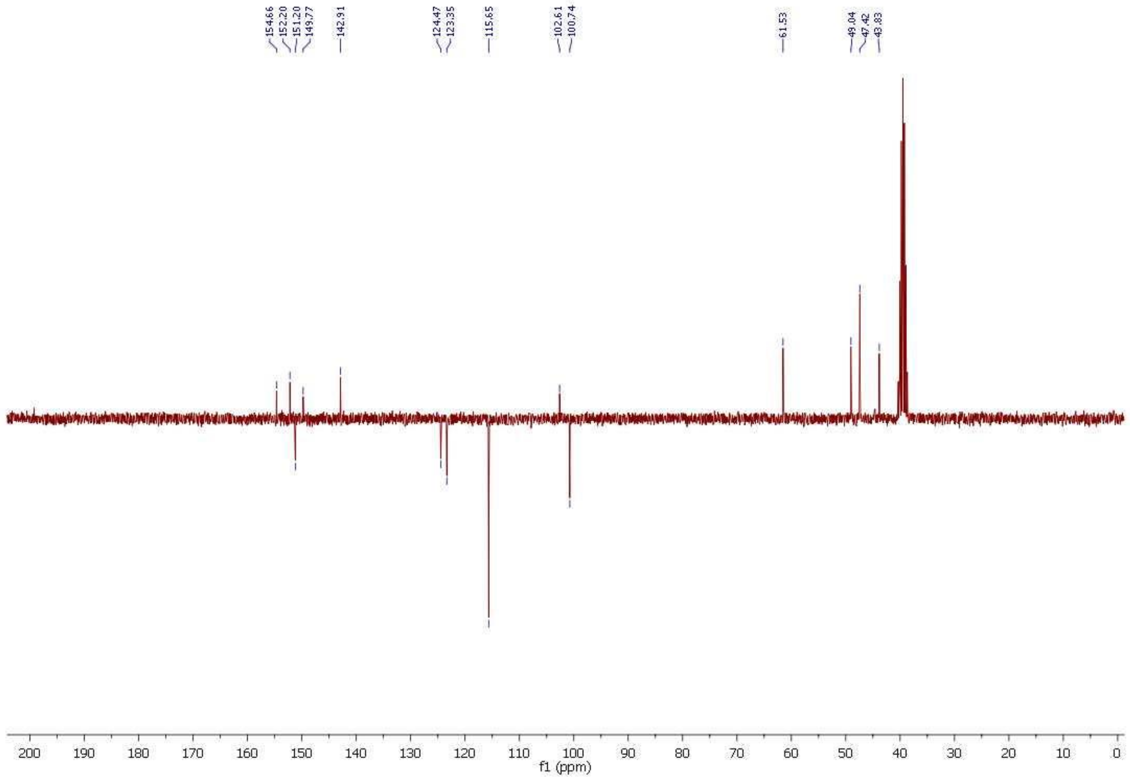

Fig. S19 a) ^{1}H NMR and b) ^{13}C NMR of compd. **7b**

a)

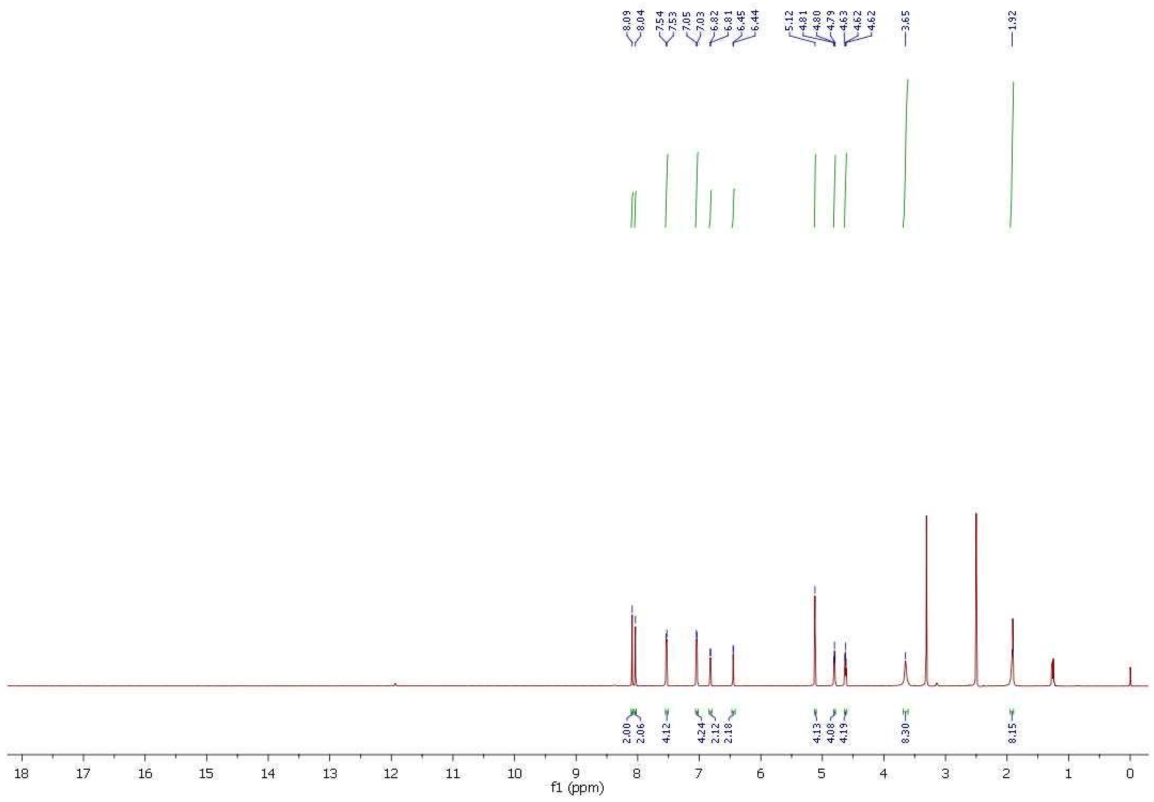

b)

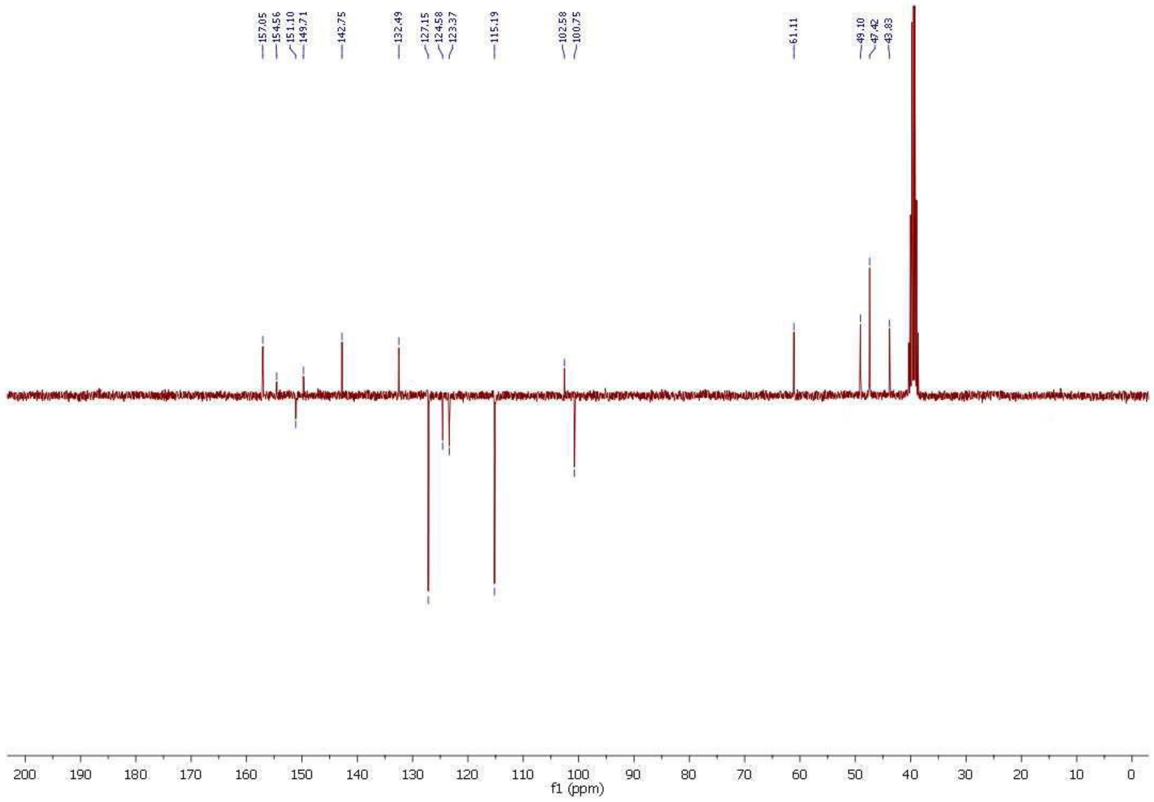

Fig. S20 a) ^{1}H NMR and b) ^{13}C NMR of compd. **7c**

a)

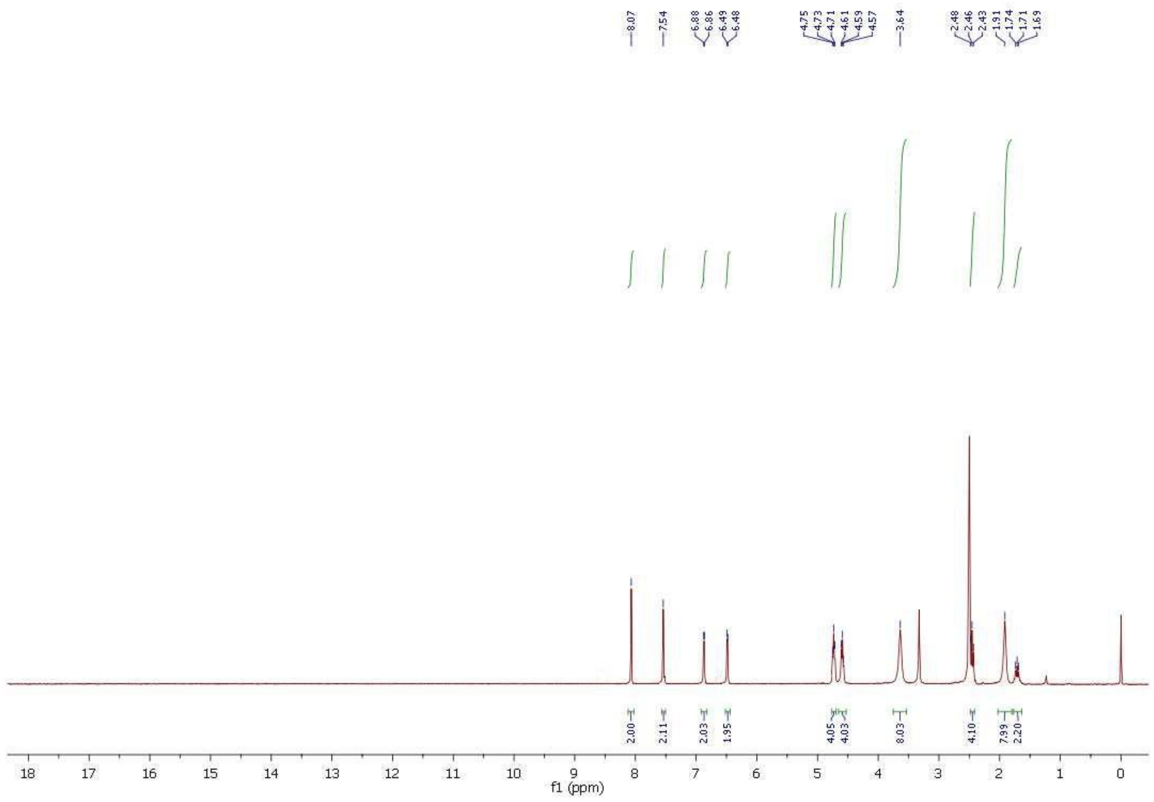

b)

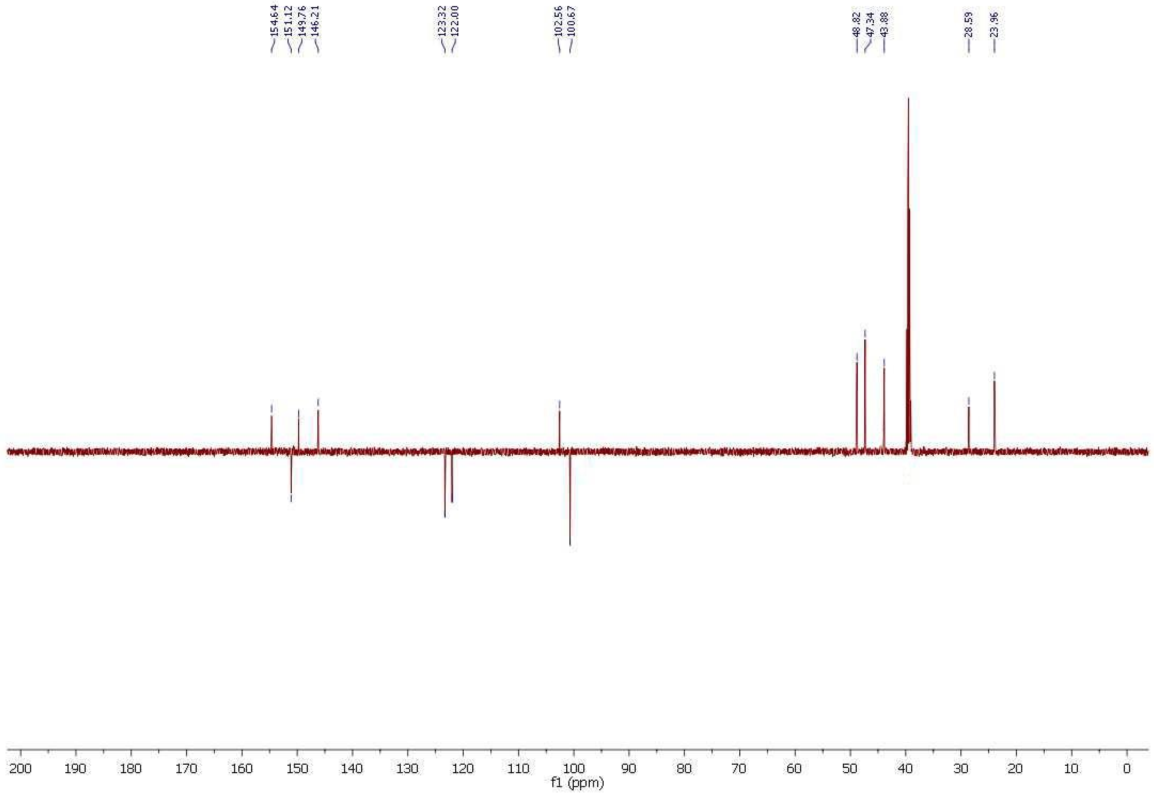

Fig. S21 a) ^{1}H NMR and b) ^{13}C NMR of compd. **7d**

a)

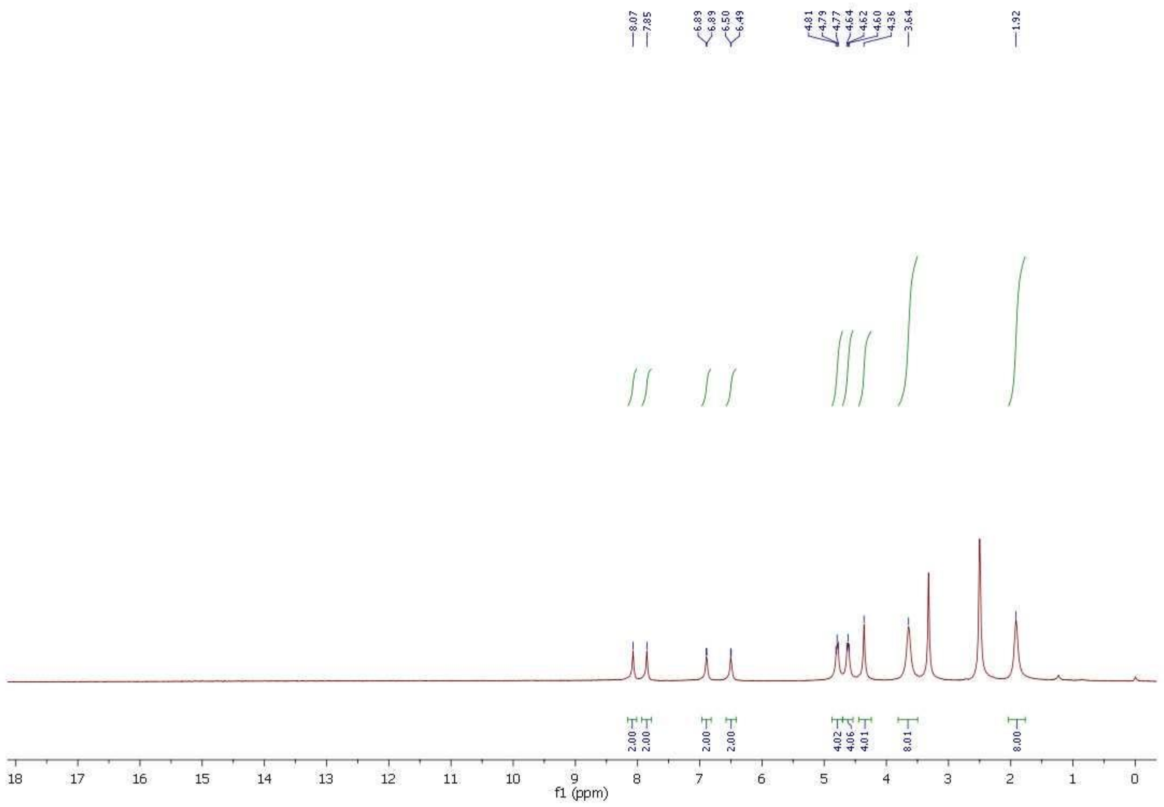

b)

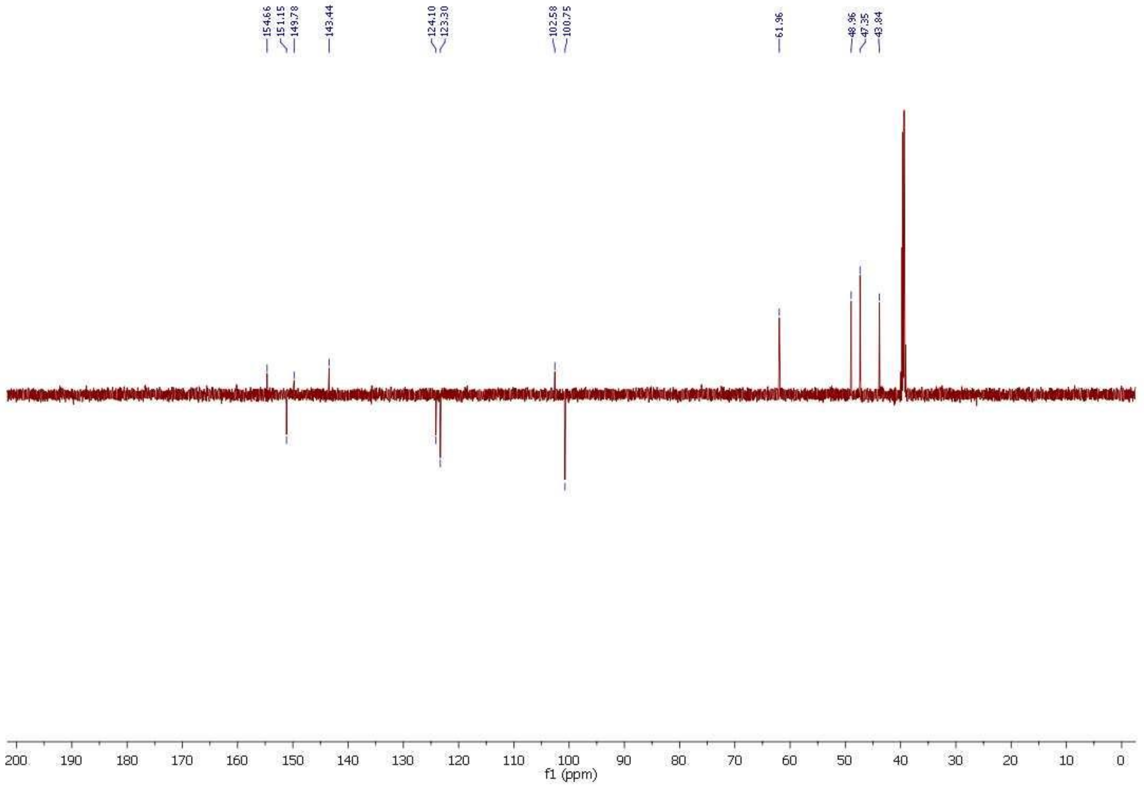

Fig. S22 a) ^{1}H NMR and b) ^{13}C NMR of compd. **8g NS-75-1**

a)

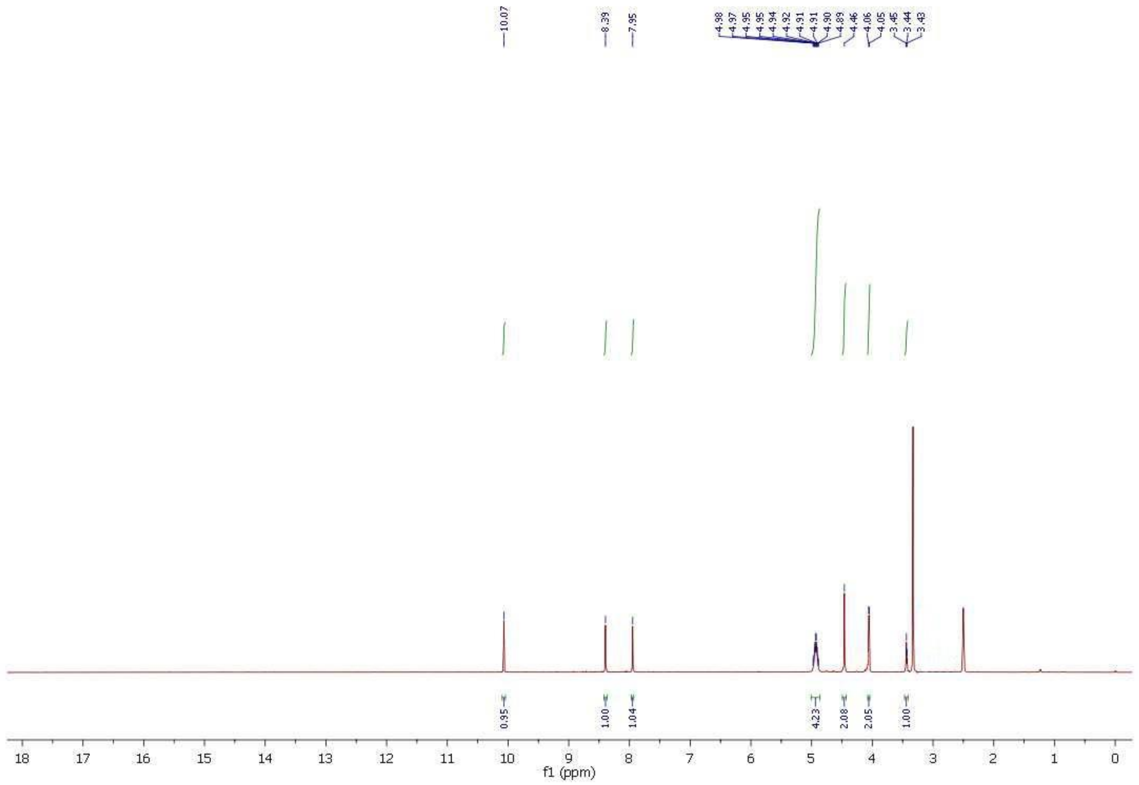

b)

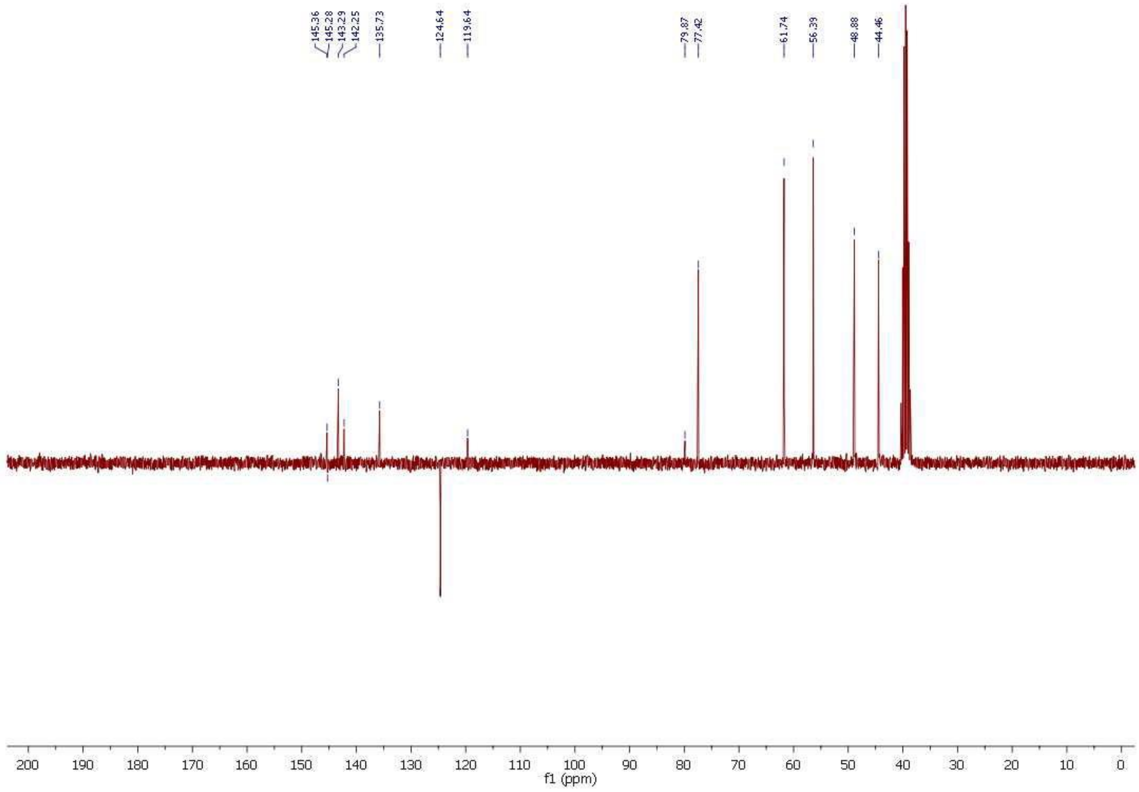

Fig. S23 a) ^{1}H NMR and b) ^{13}C NMR of compd. **9a A-260-Bis**

a)

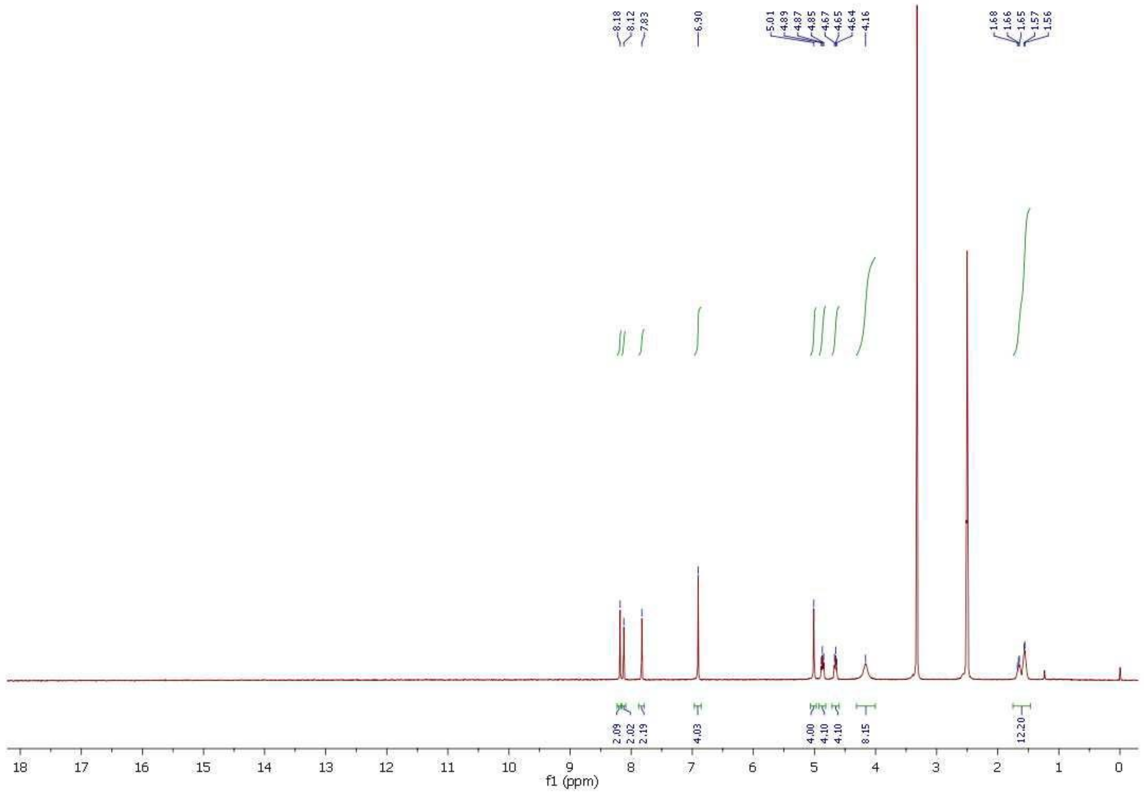

b)

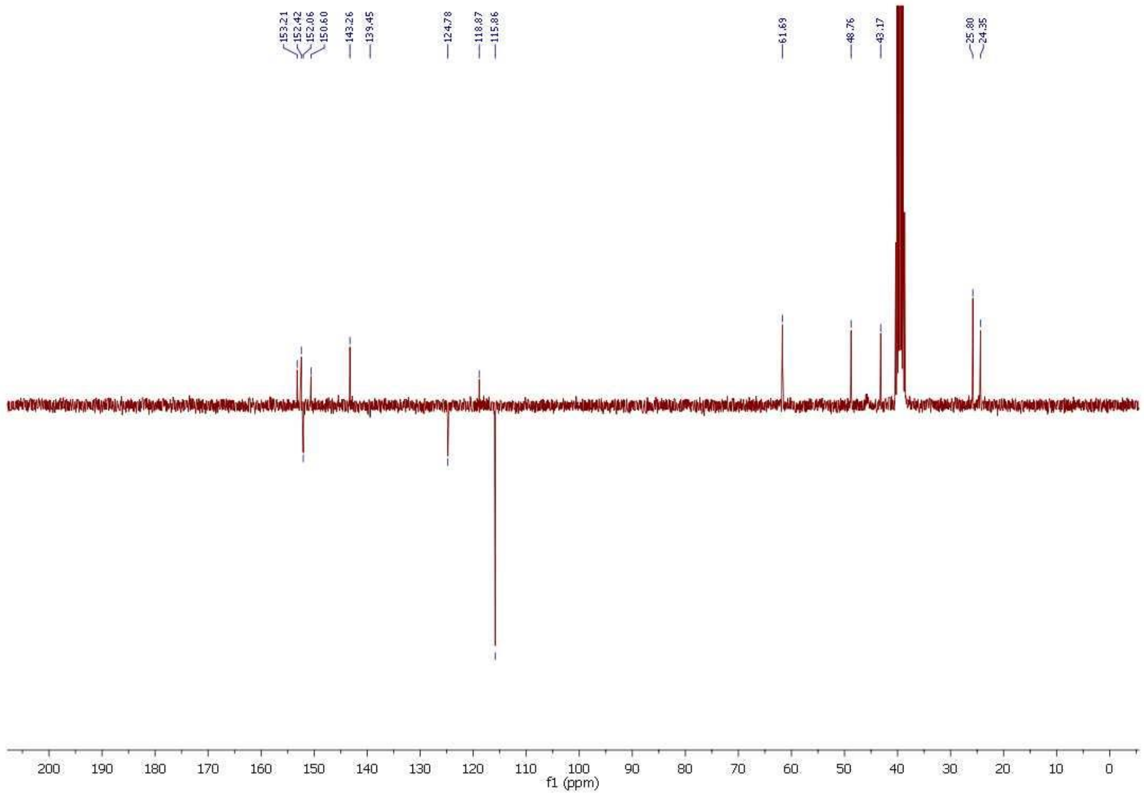

Fig. S24 a) ^{1}H NMR and b) ^{13}C NMR of compd. **9b A-261-Bis**

a)

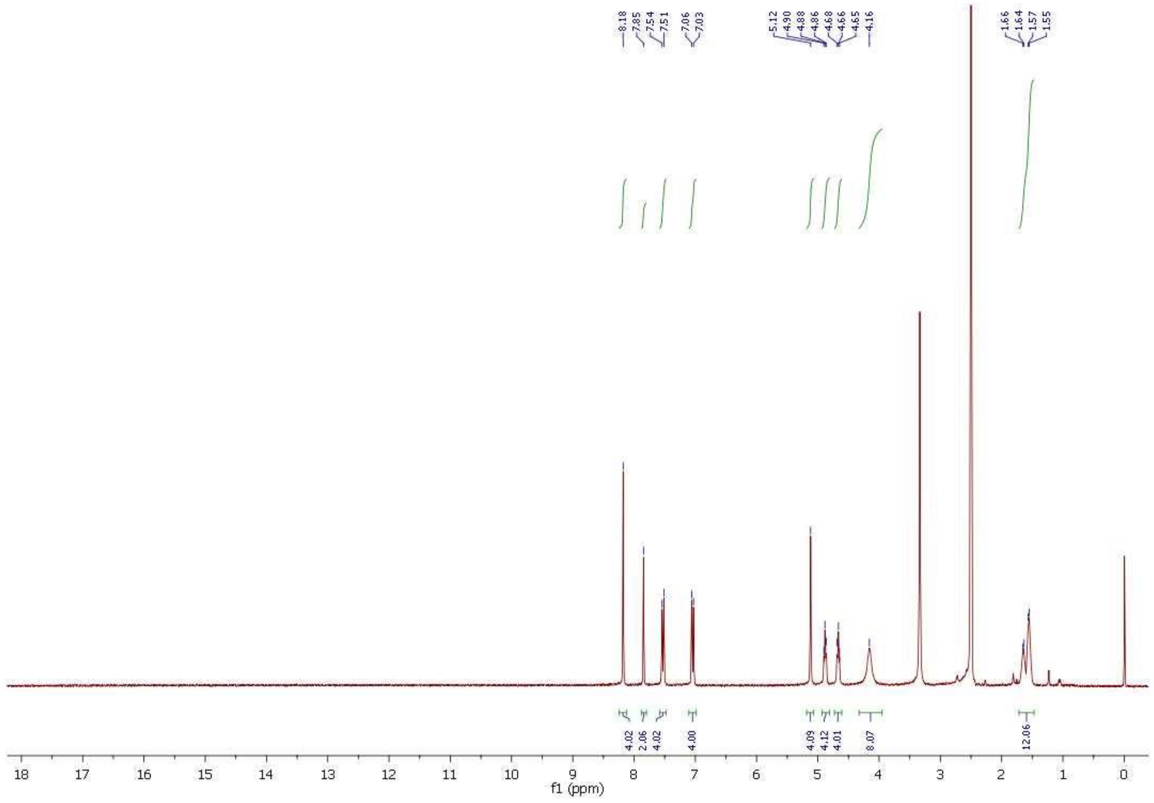

b)

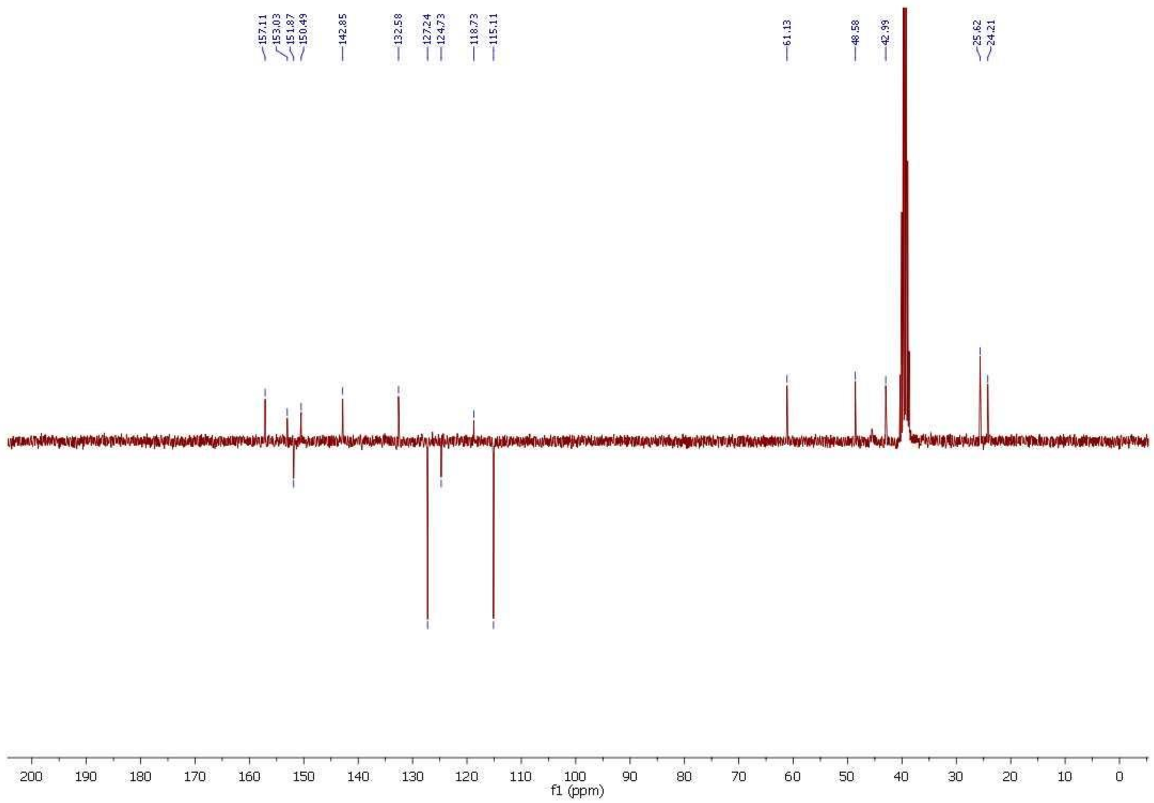

Fig. S25 a) ^{1}H NMR and b) ^{13}C NMR of compd. **9c A-262-Bis**

a)

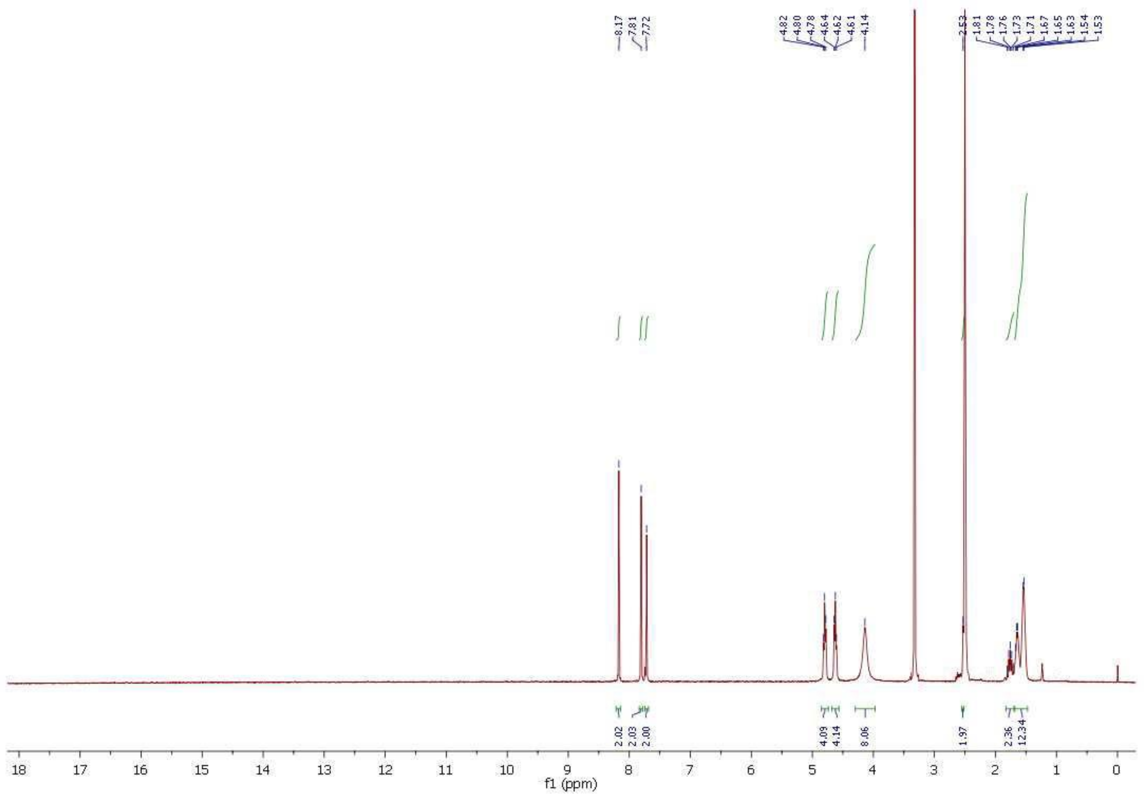

b)

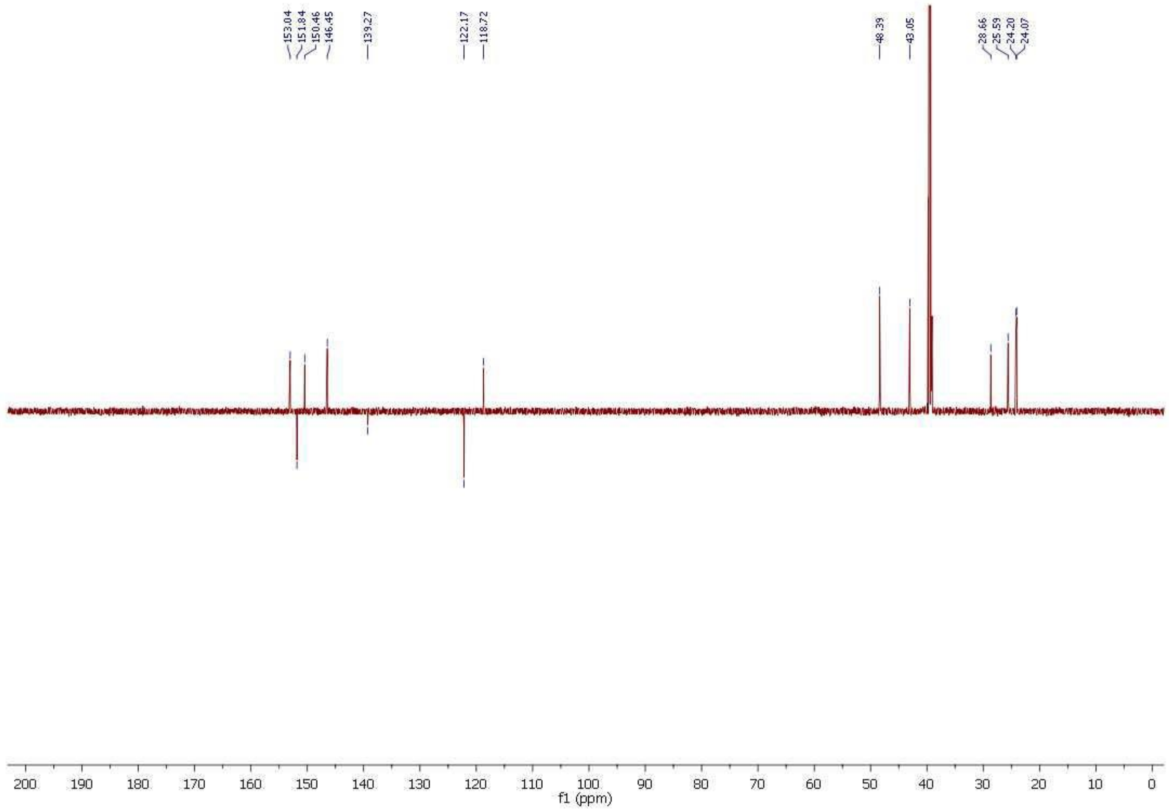

Fig. S26 a) ^{1}H NMR and b) ^{13}C NMR of compd. **9d A-266-Bis**

a)

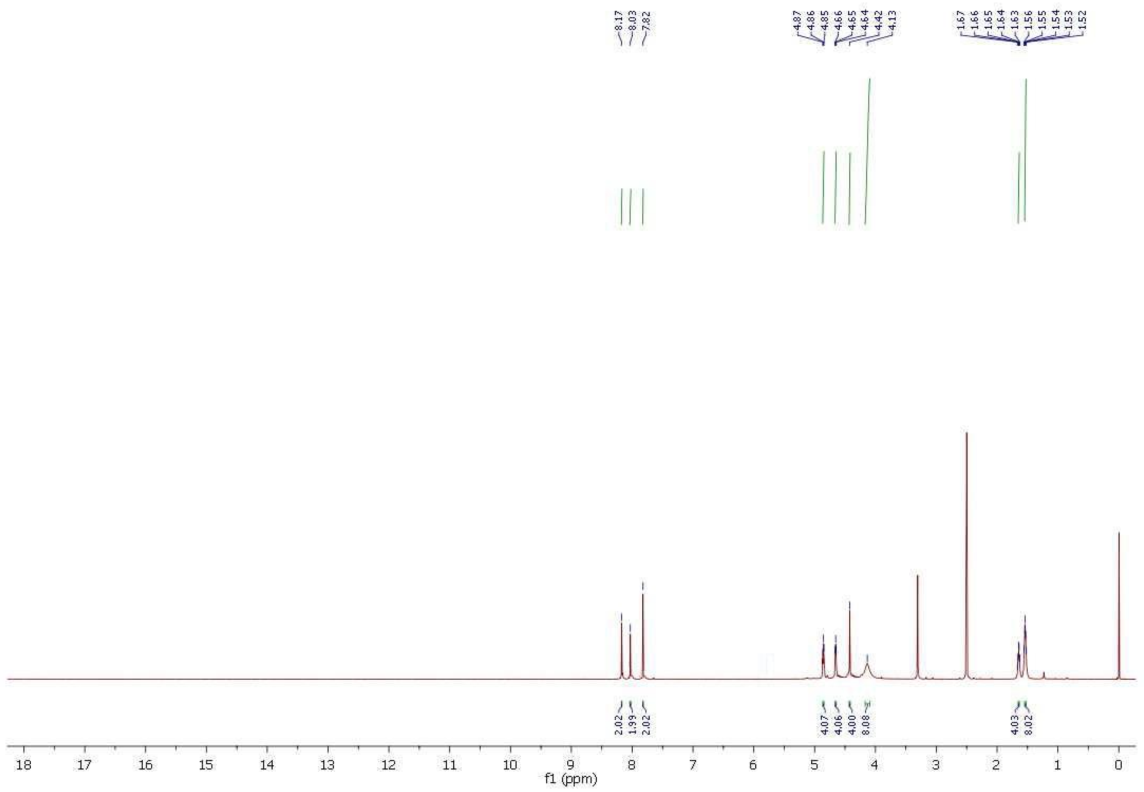

b)

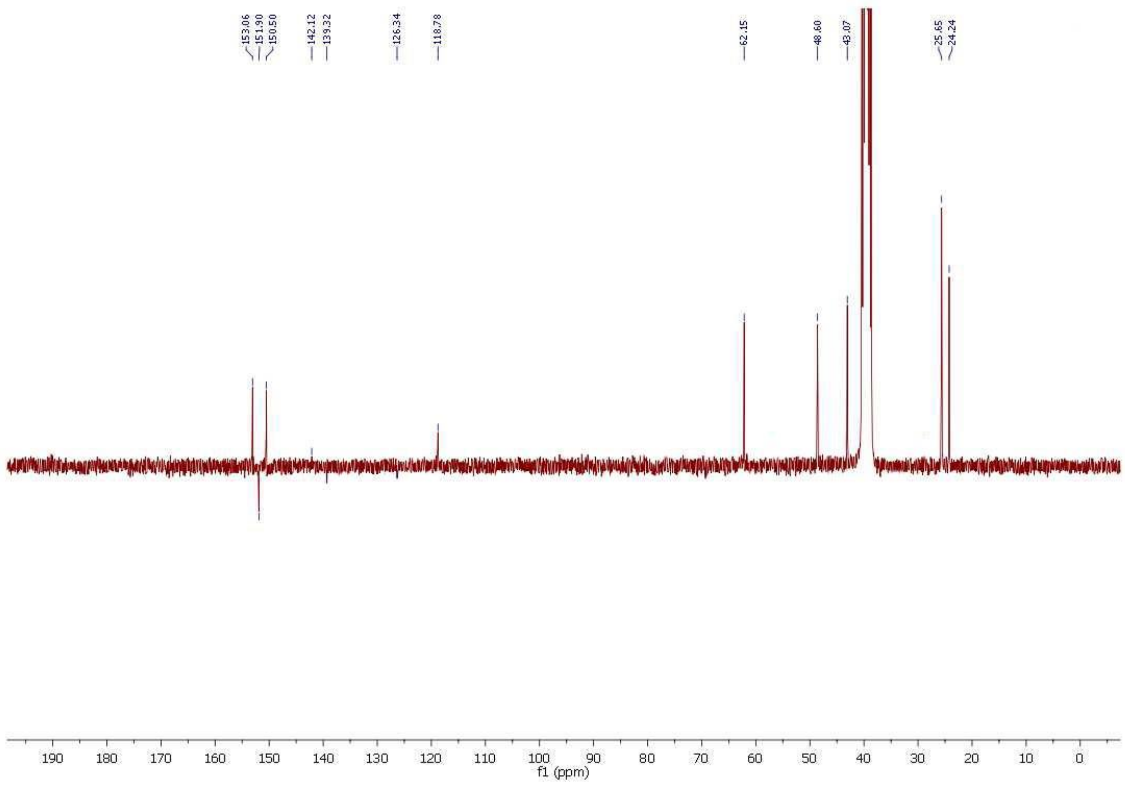

Fig. S27 a) ^{1}H NMR and b) ^{13}C NMR of compd. **9e A-260-Mono**

a)

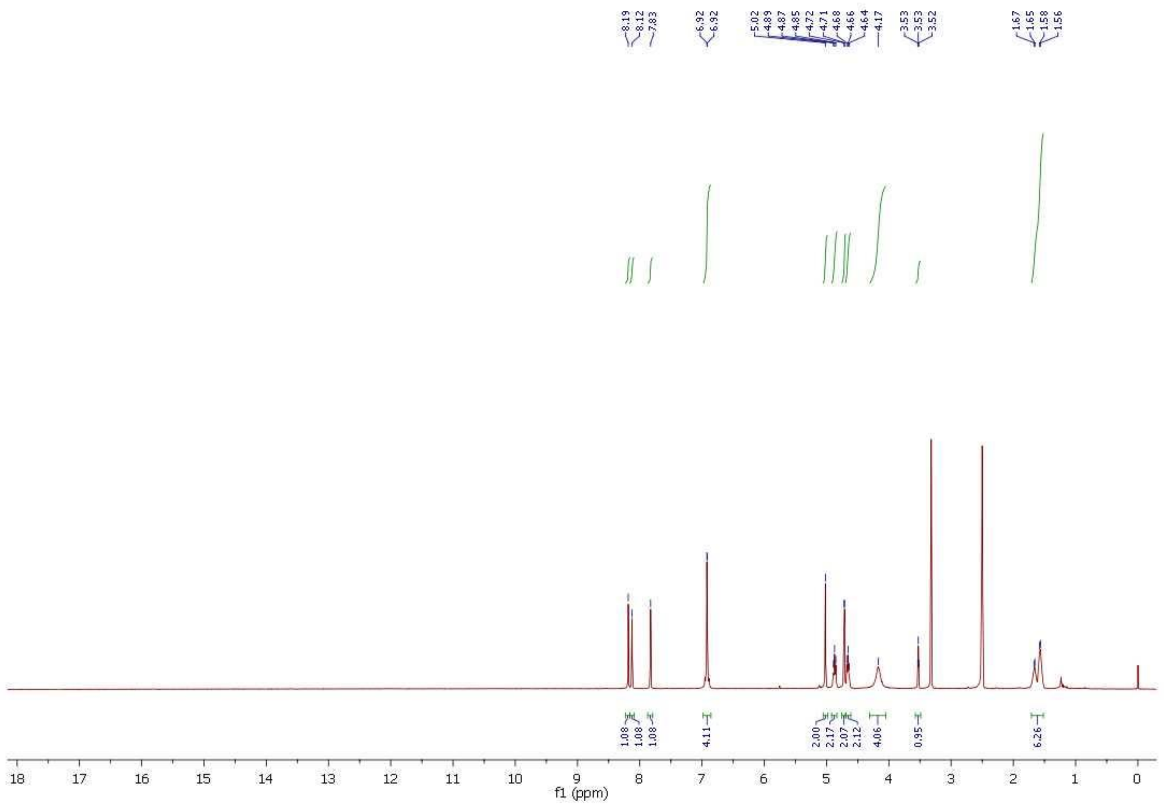

b)

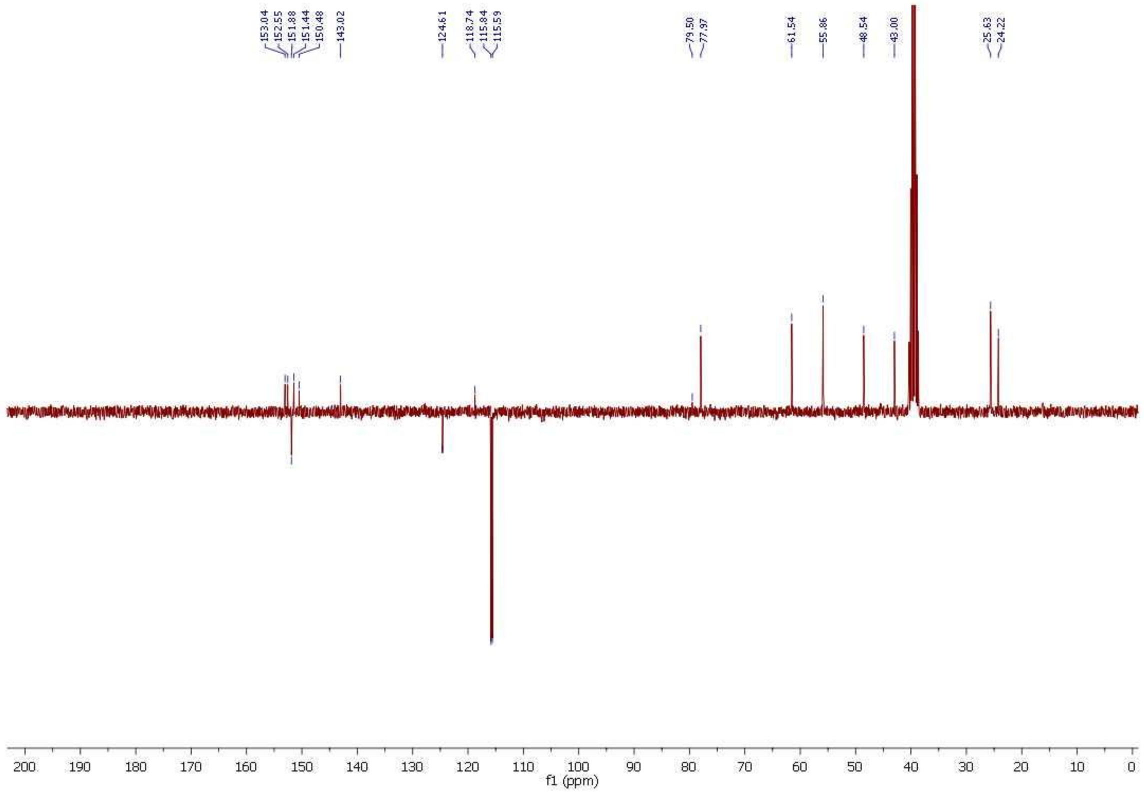

Fig. S28 a) ^{1}H NMR and b) ^{13}C NMR of compd. **9f A-261-Mono**

a)

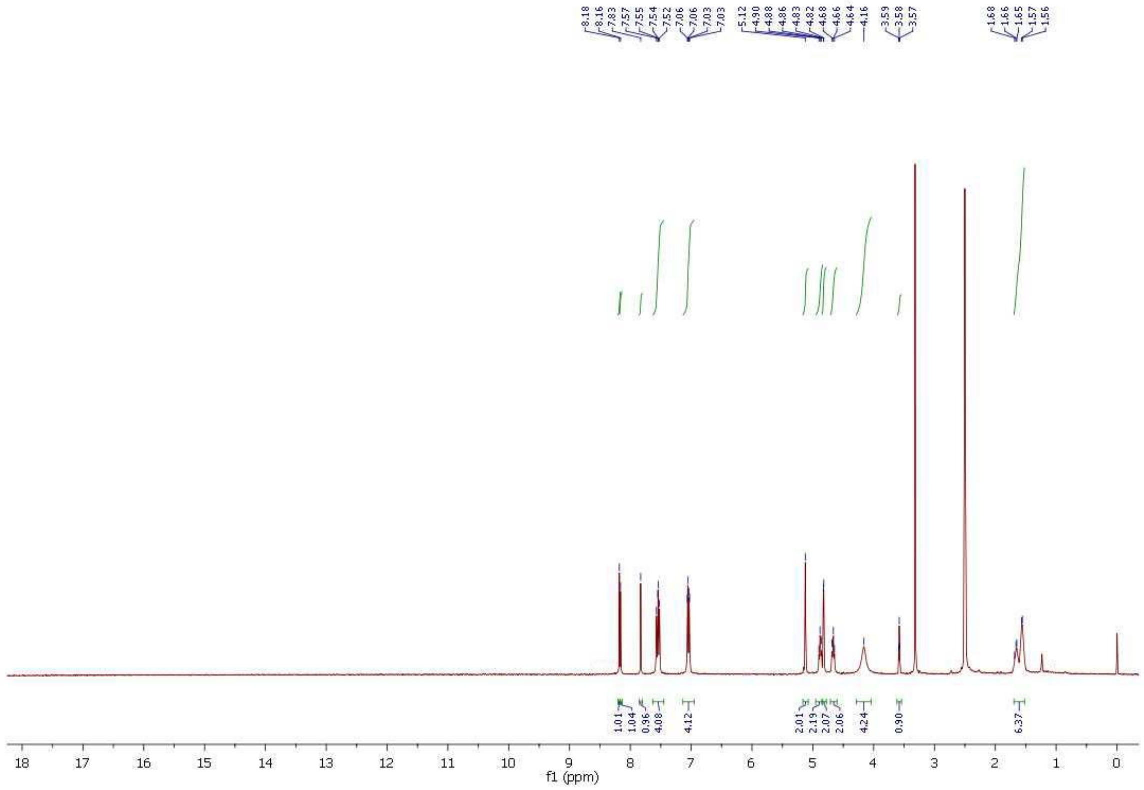

b)

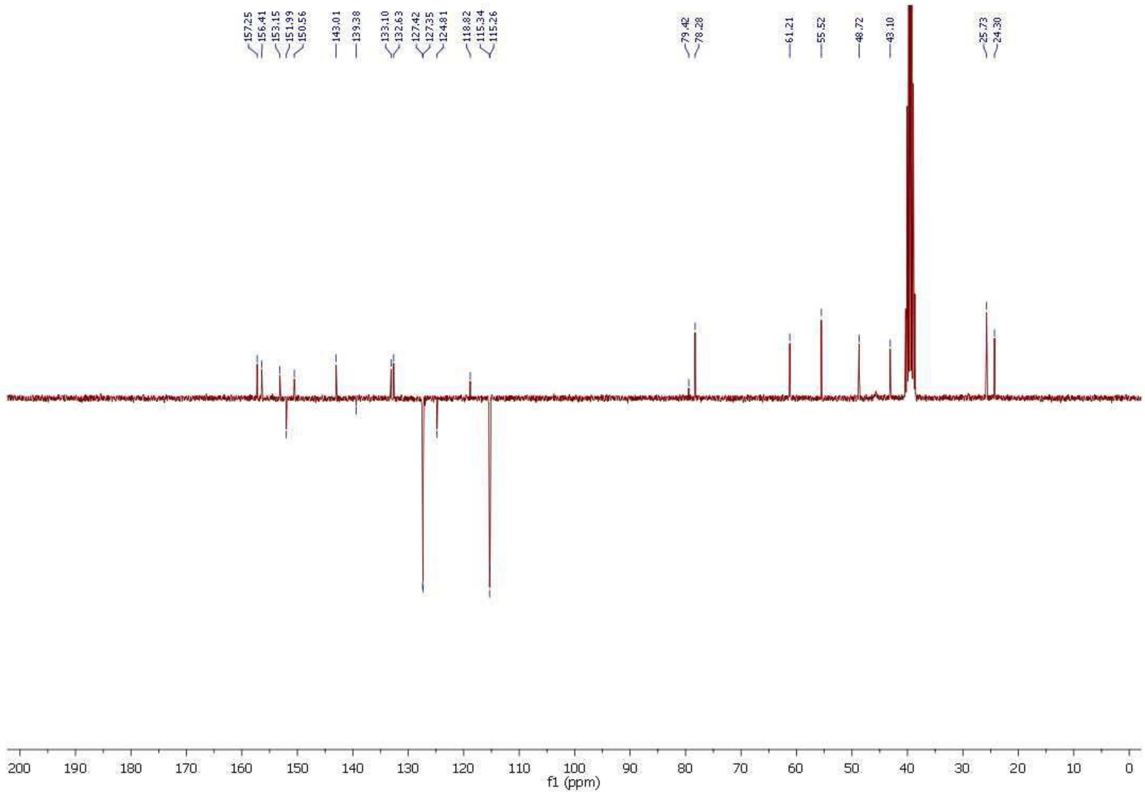

Fig. S29 a) ^{1}H NMR and b) ^{13}C NMR of compd. **9g A-262-Mono**

a)

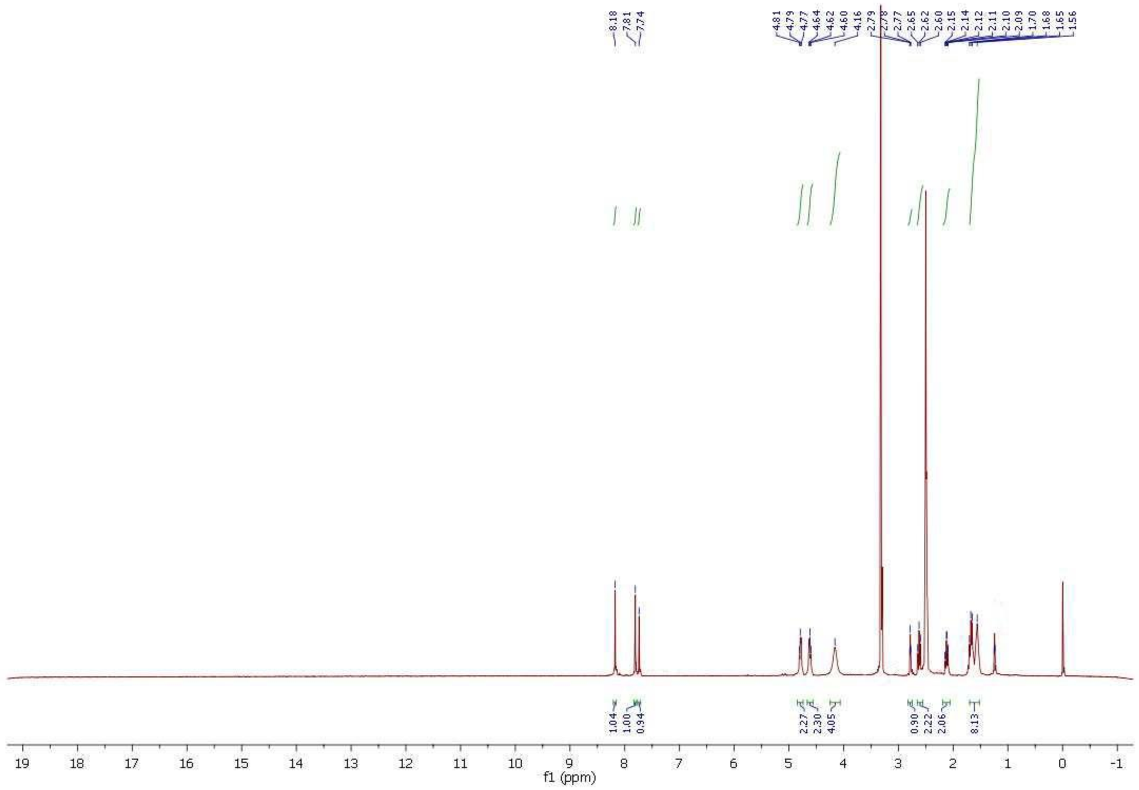

b)

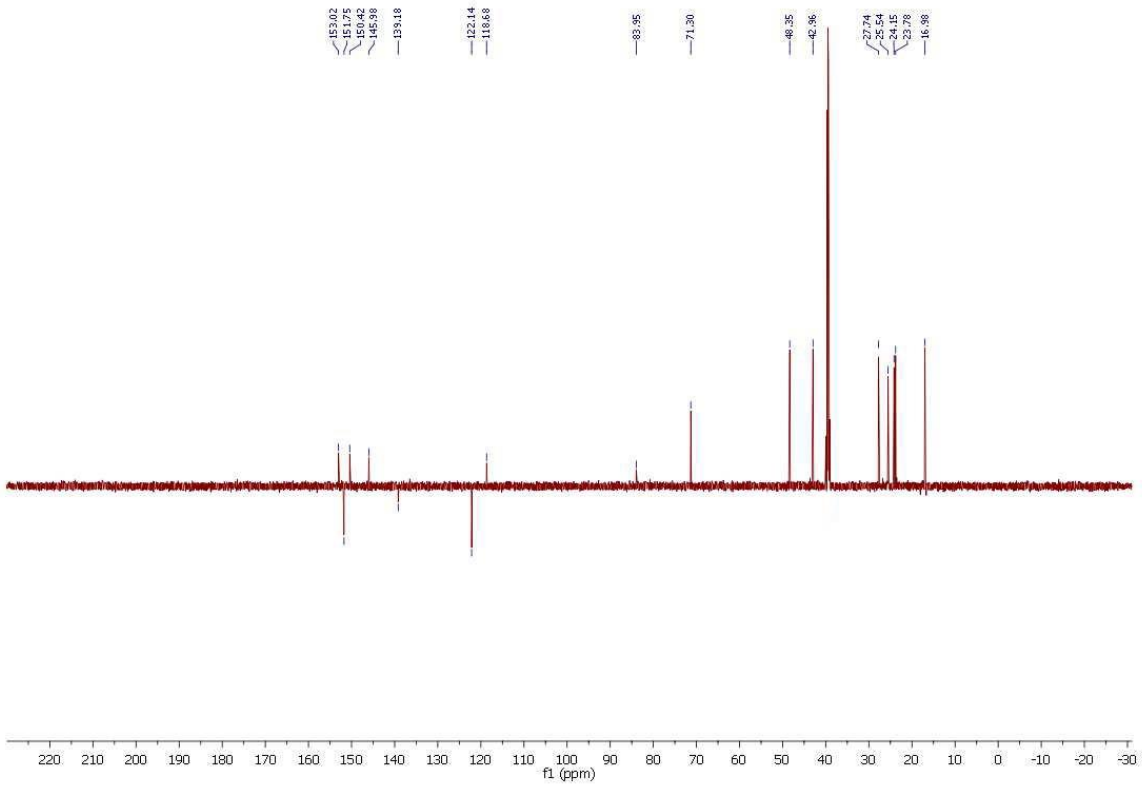

Fig. S30 a) ^{1}H NMR and b) ^{13}C NMR of compd. **10a A-263**

a)

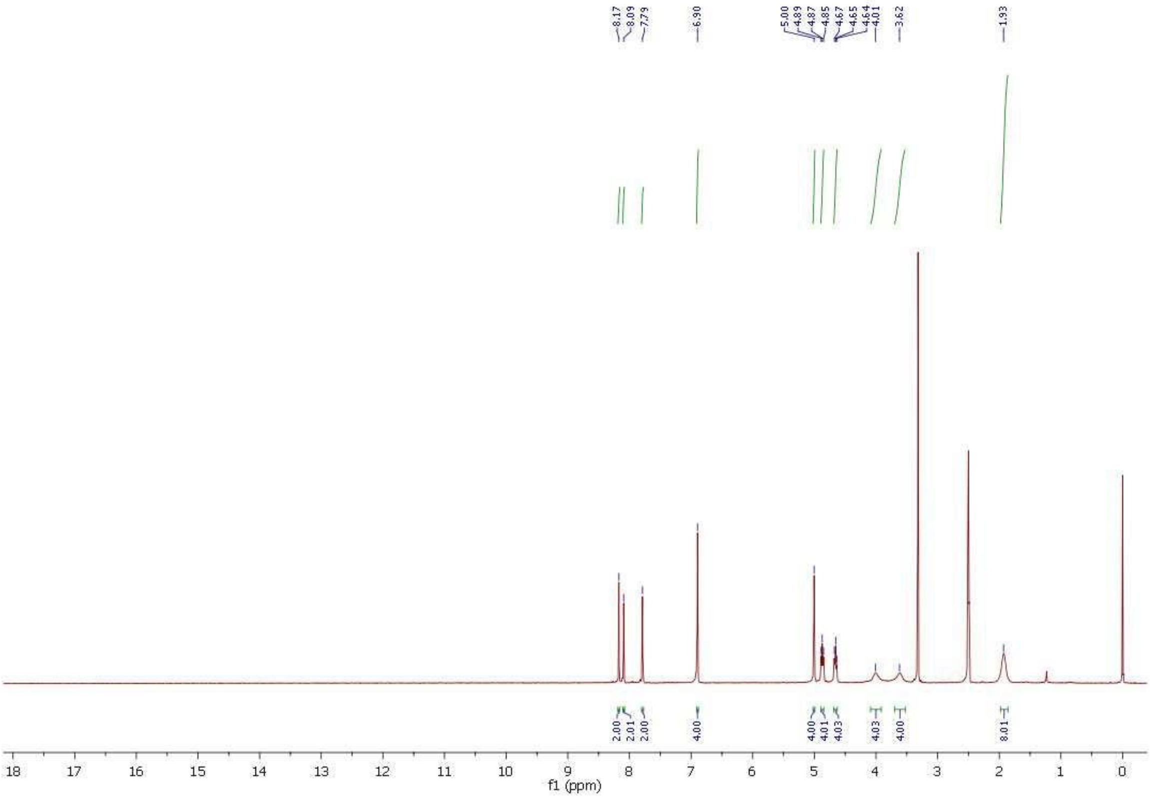

b)

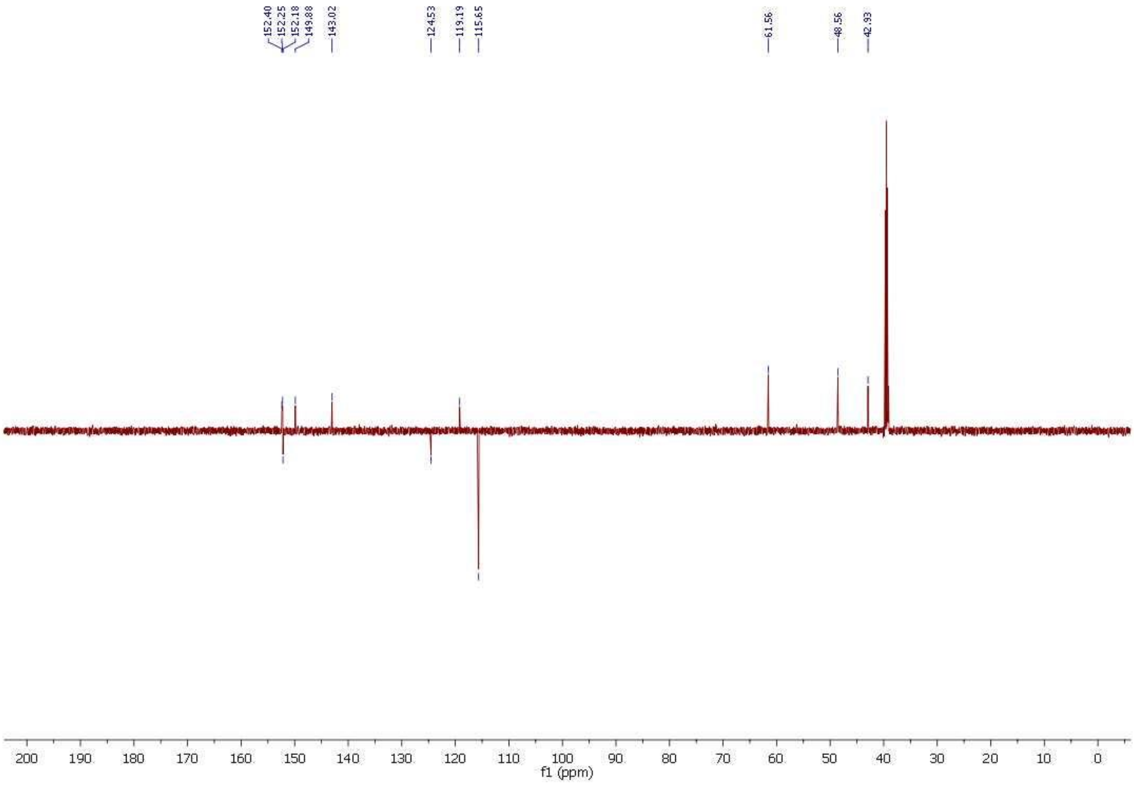

Fig. S31 a) ^{1}H NMR and b) ^{13}C NMR of compd. **10b A-265**

a)

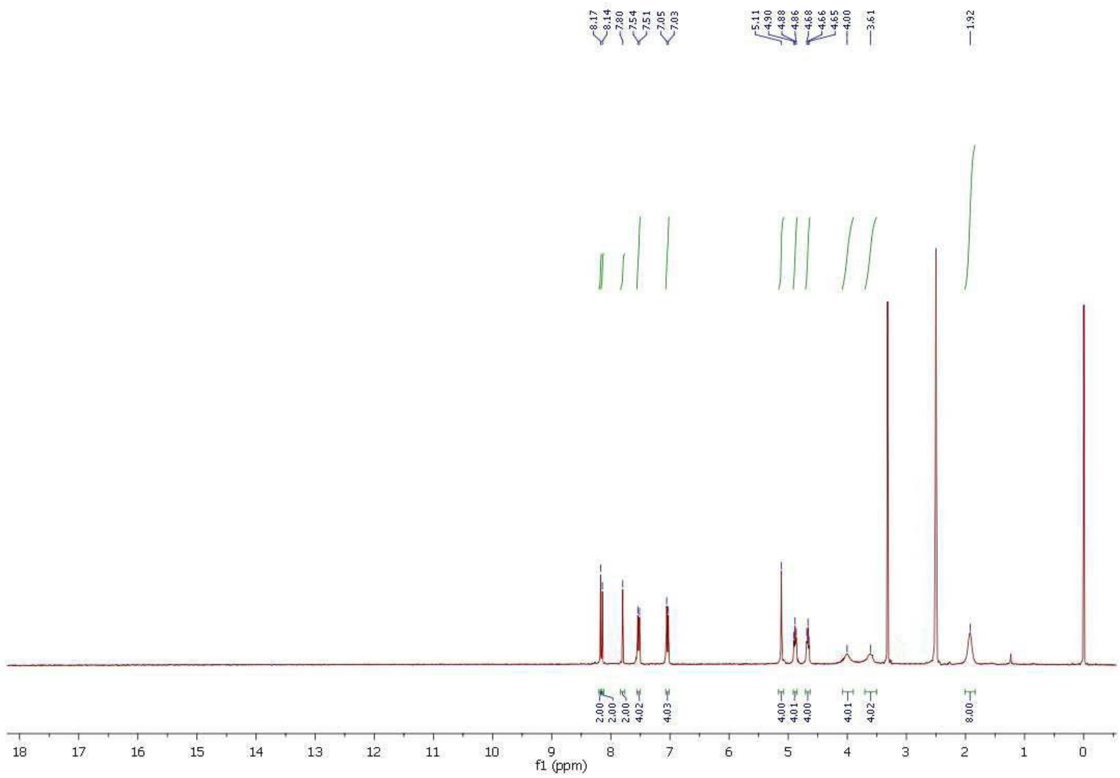

b)

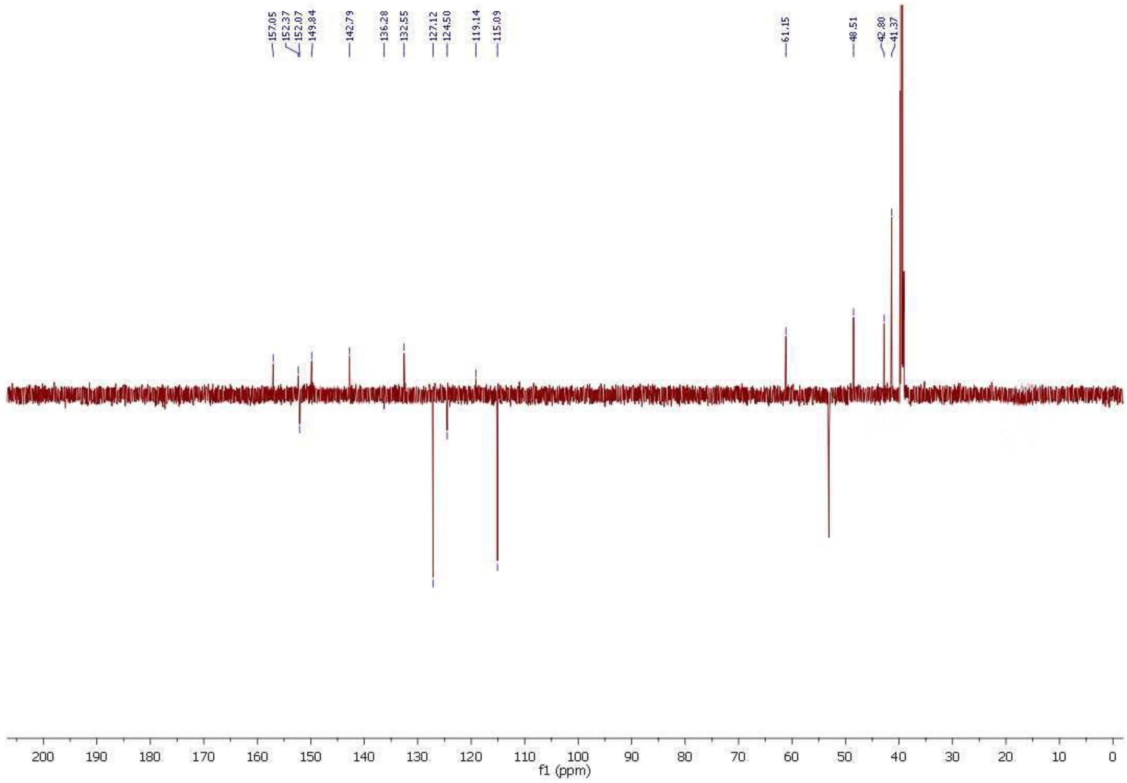

Fig. S32 a) ^{1}H NMR and b) ^{13}C NMR of compd. **10c A-264**

a)

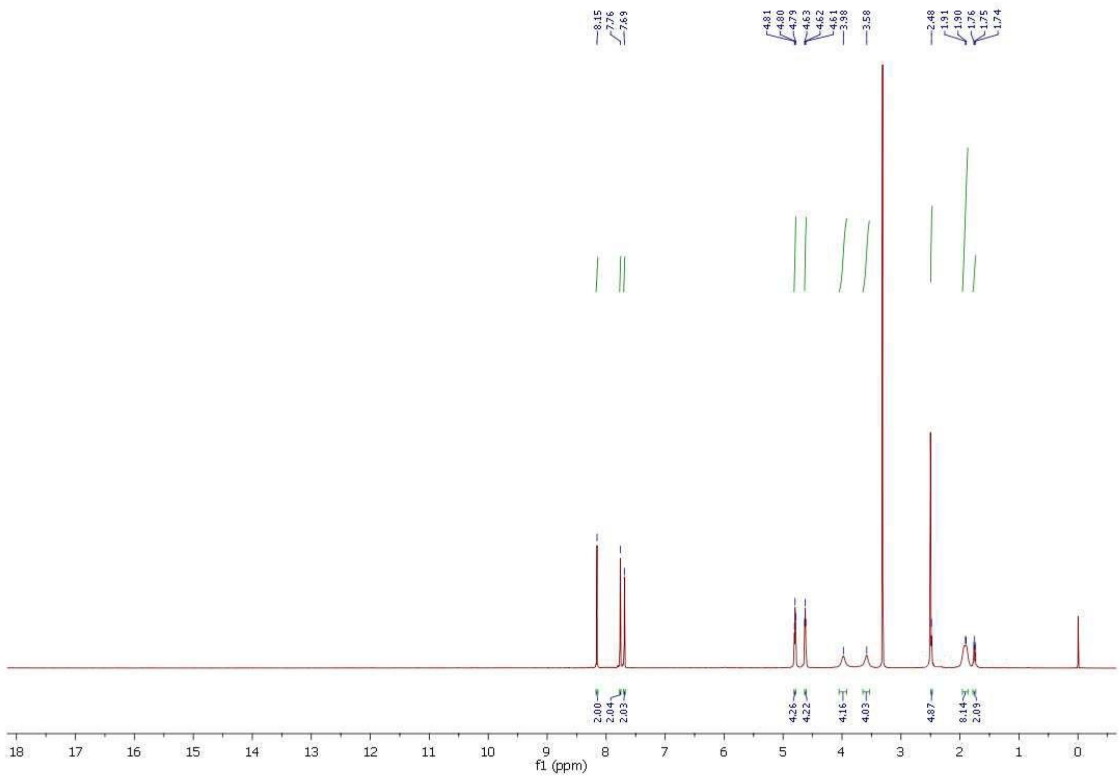

b)

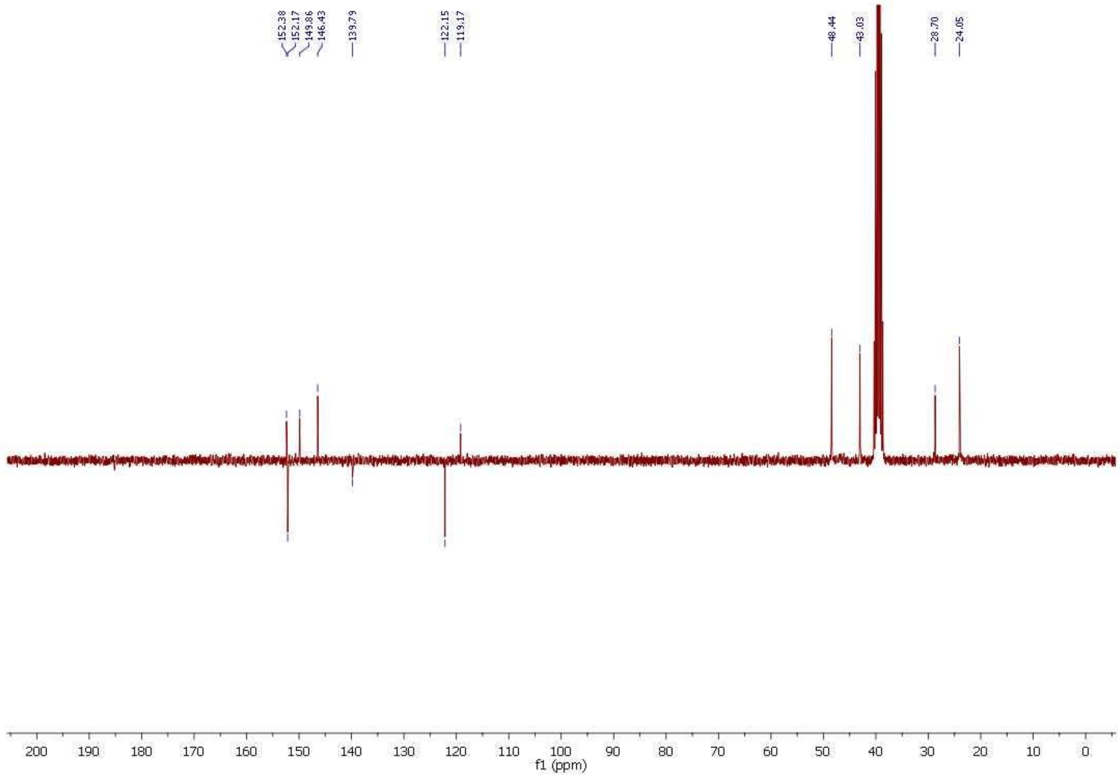

Fig. S33 a) ^{1}H NMR and b) ^{13}C NMR of compd. **10d A-267**

a)

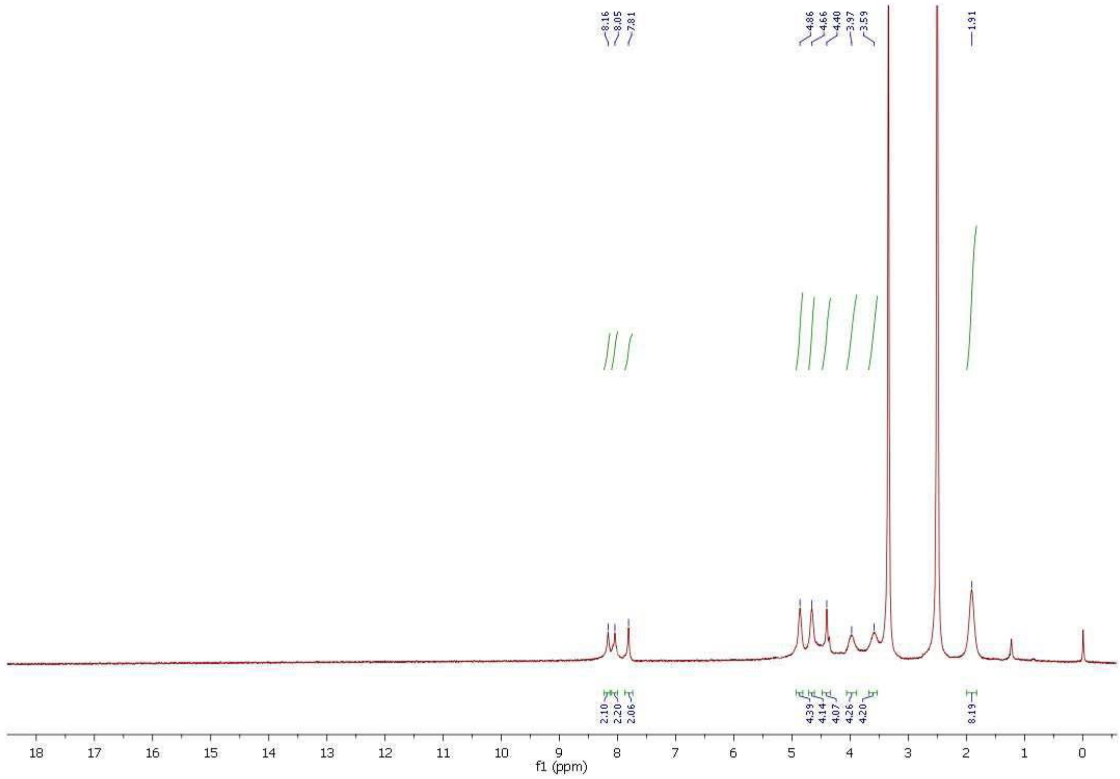

b)

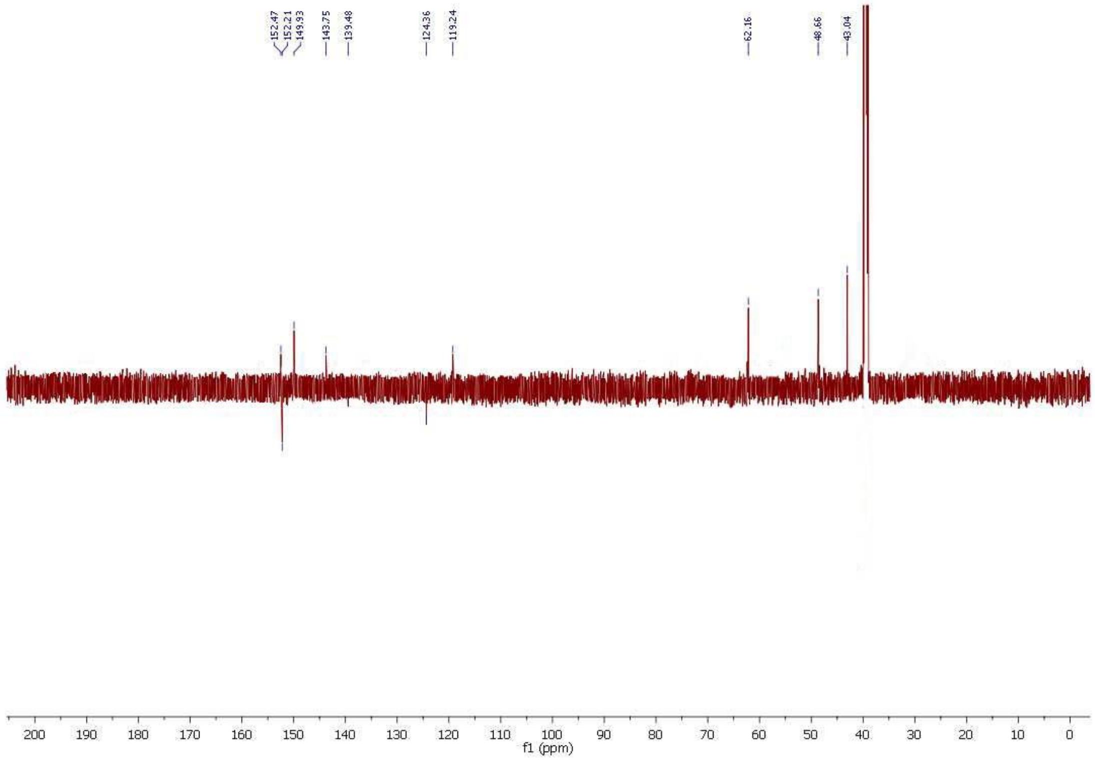

Fig. S34 a) ^{1}H NMR and b) ^{13}C NMR of compd. **10f**

a)

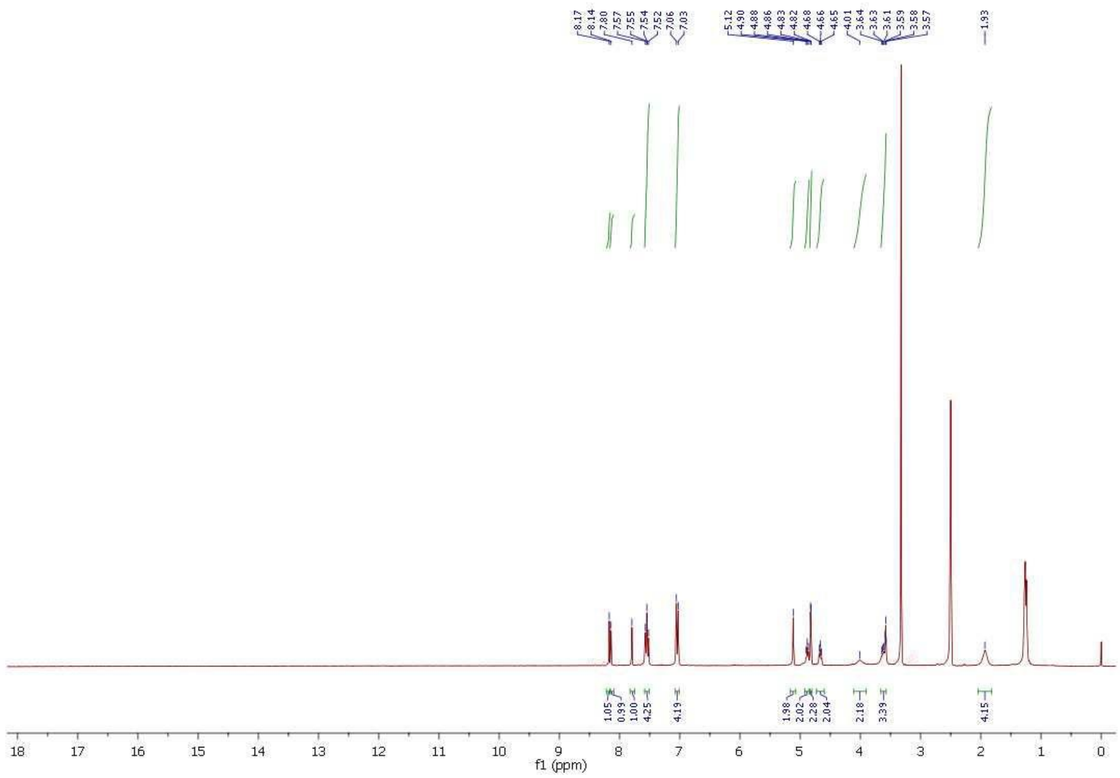

b)

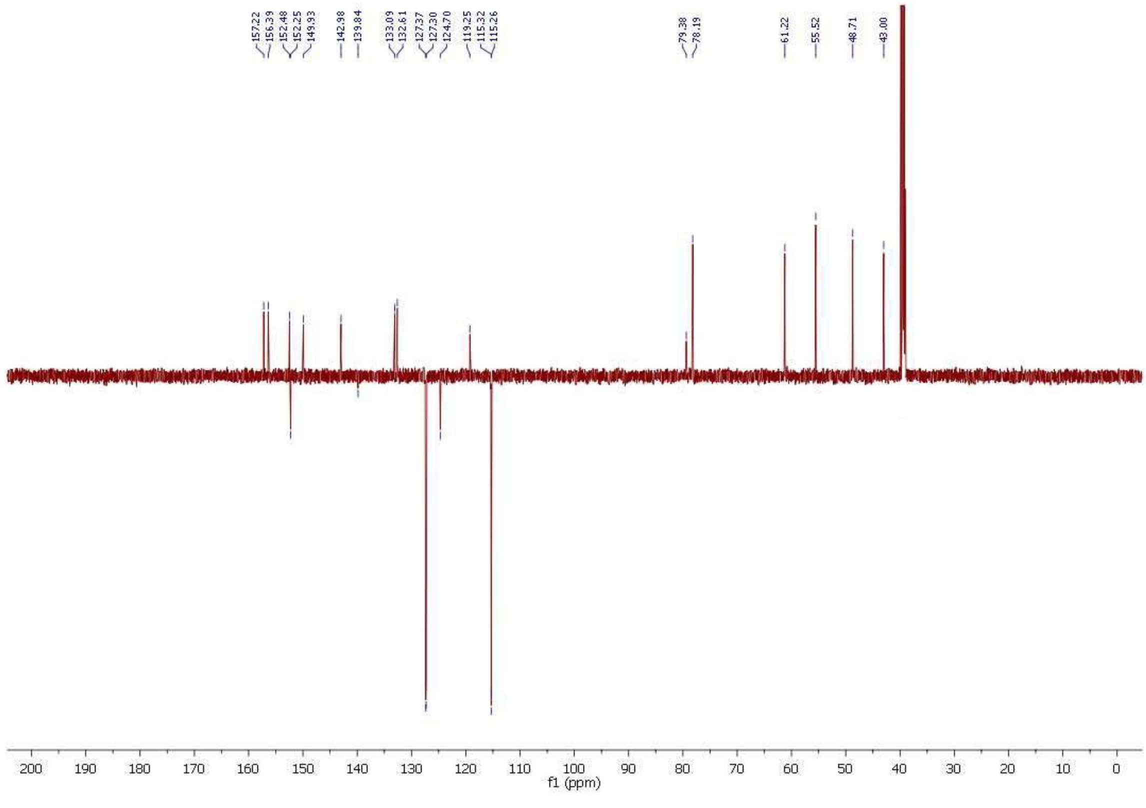

Fig. S35 a) ^{1}H NMR and b) ^{13}C NMR of compd. **11a**

a)

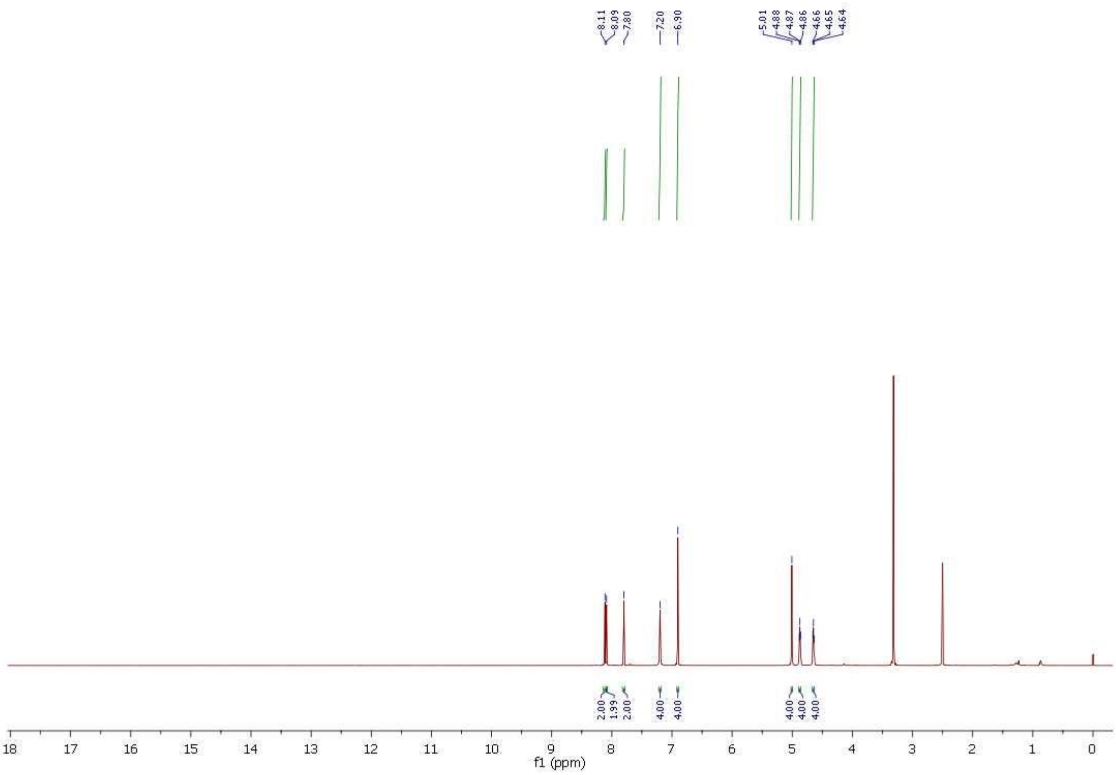

b)

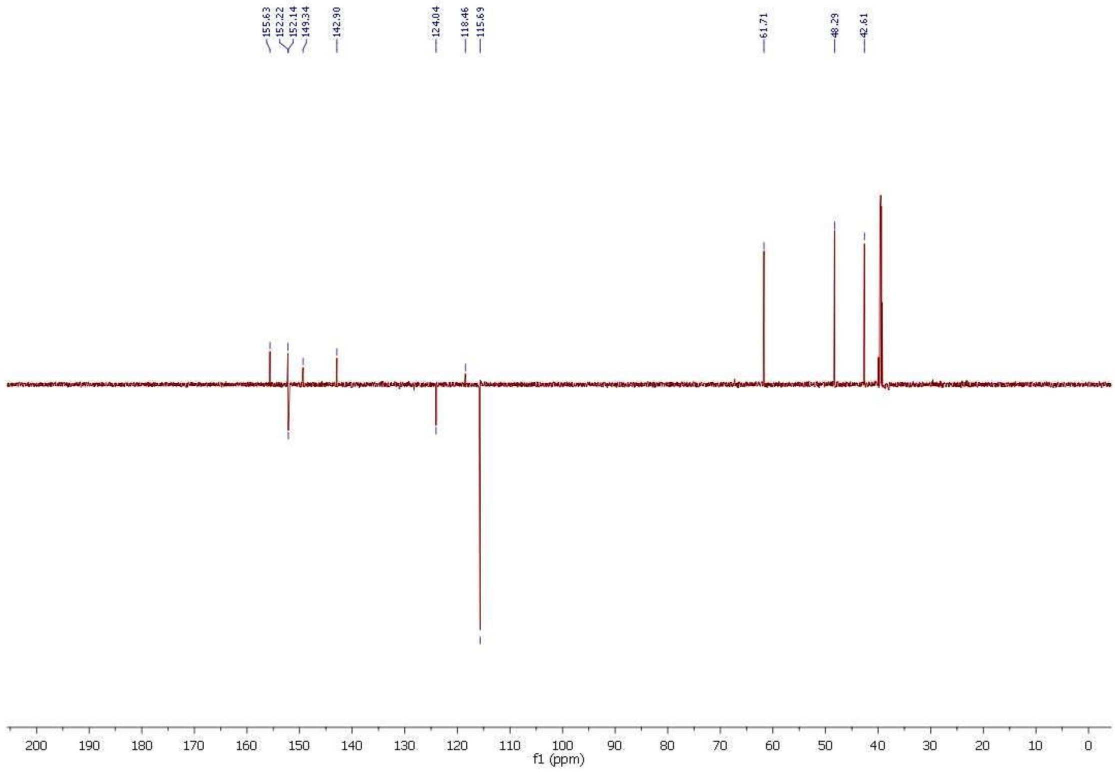

Fig. S36 a) ^{1}H NMR and b) ^{13}C NMR of compd. **11d**

a)

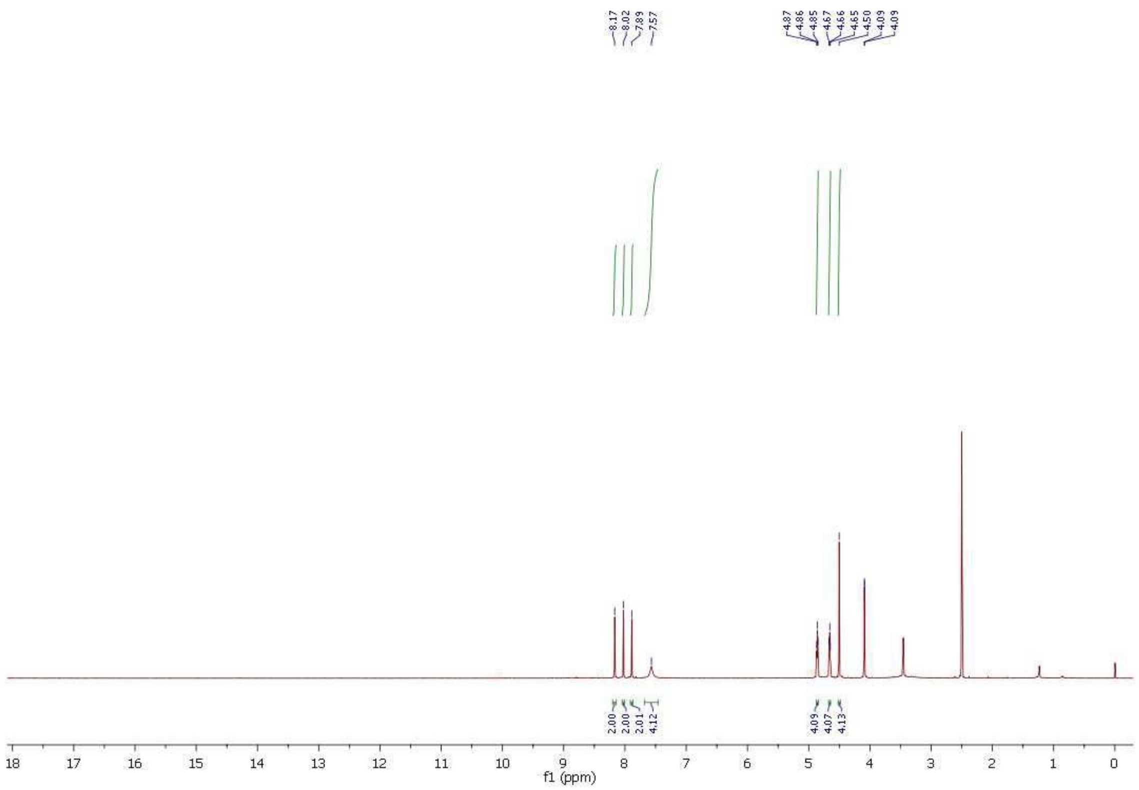

b)

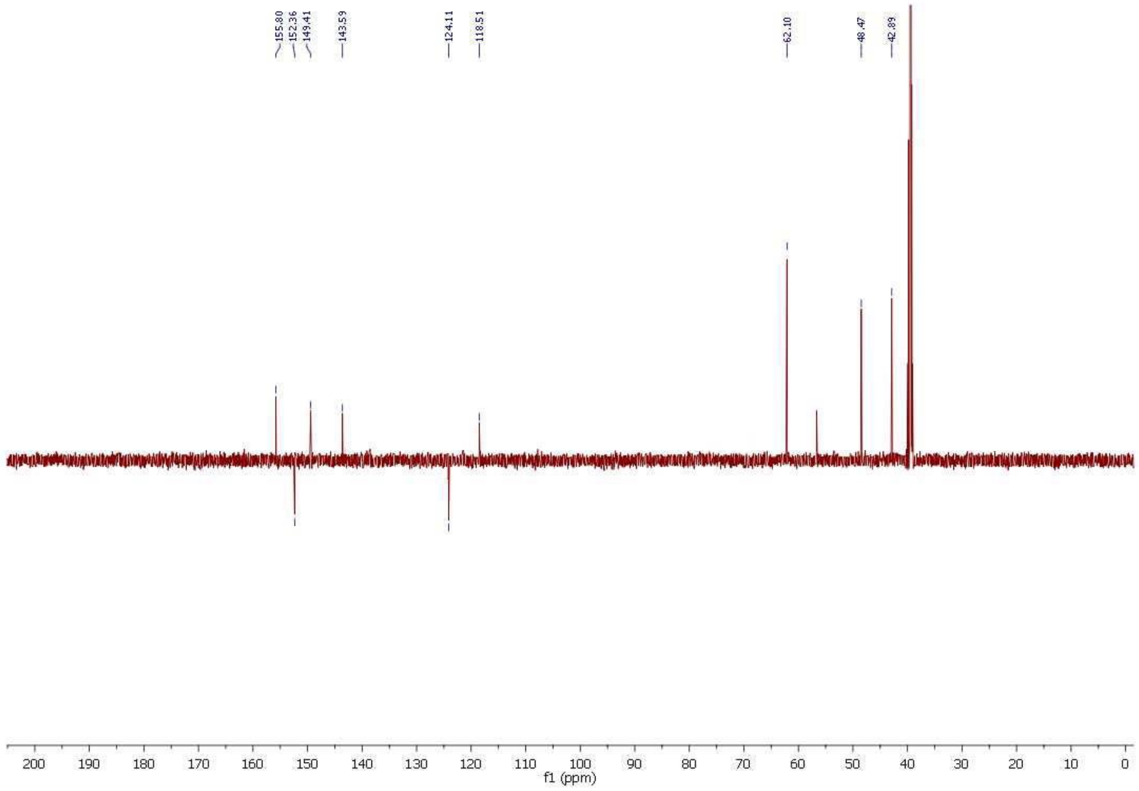

Fig. S37 a) ^{1}H NMR and b) ^{13}C NMR of compd. **11e A-198-1**

a)

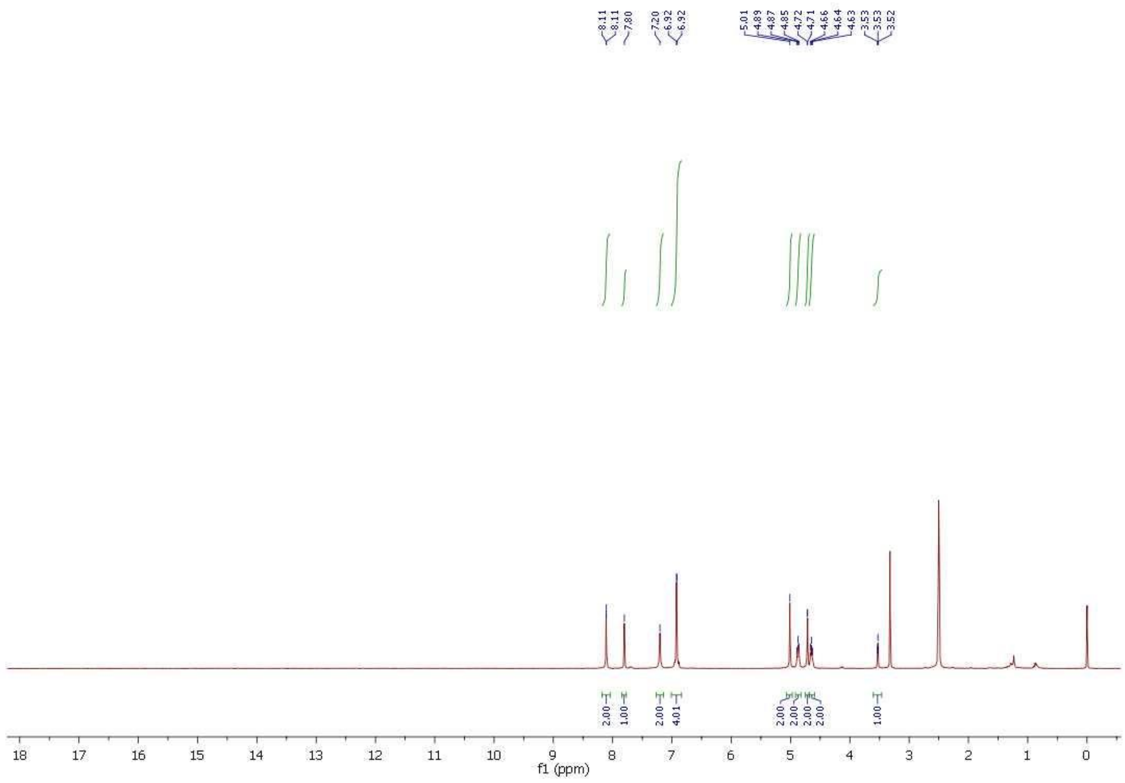

b)

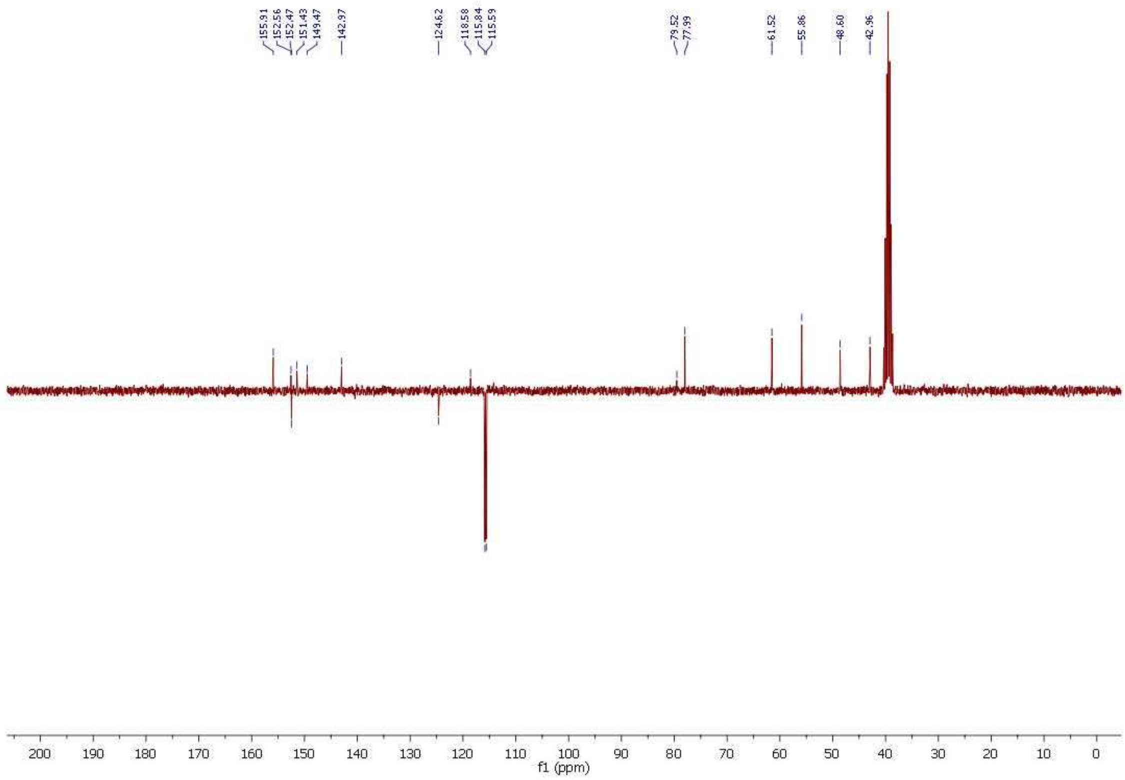

Fig. S38 a) ^{1}H NMR and b) ^{13}C NMR of compd. **11g A-199**

a)

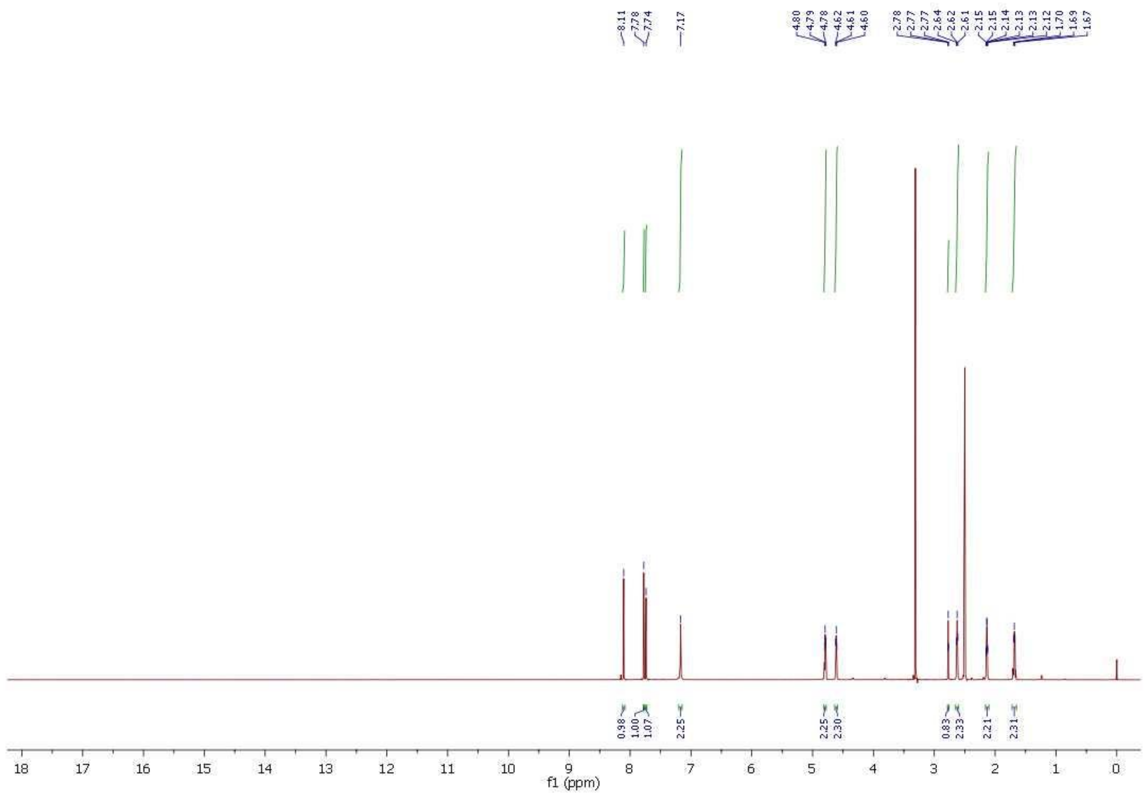

b)

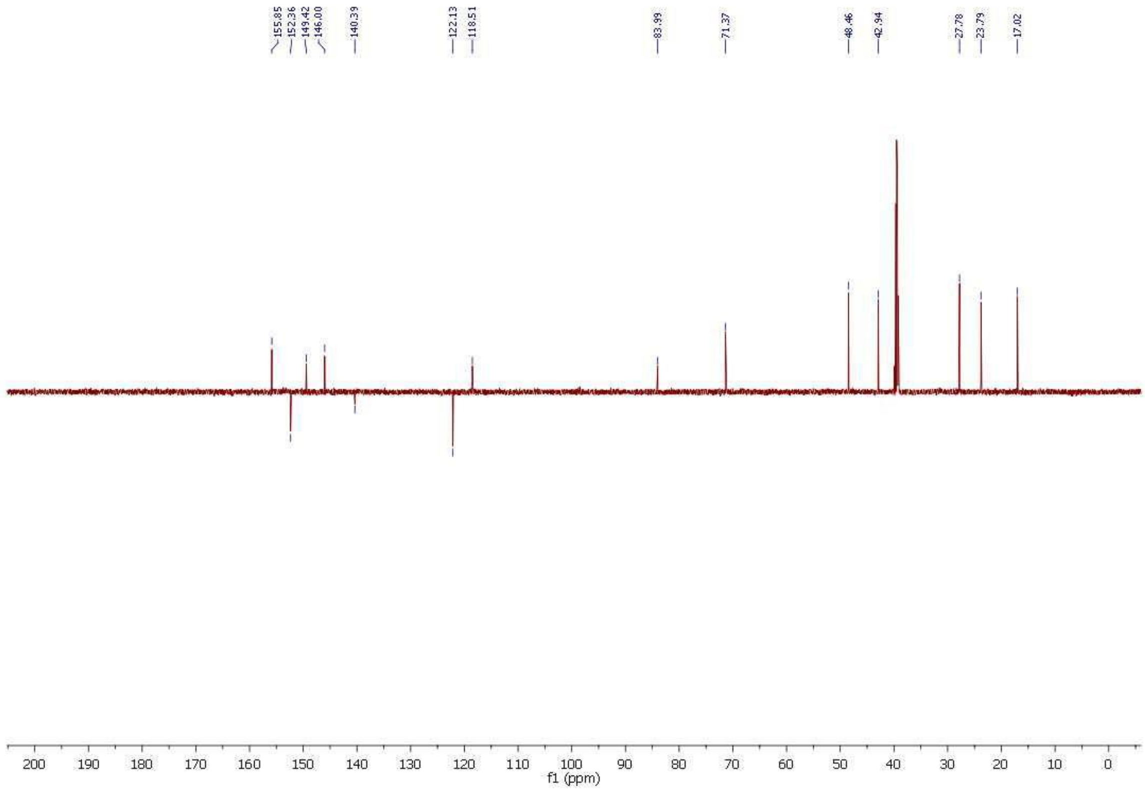

Fig. S39 a) ^{1}H NMR and b) ^{13}C NMR of compd. **11h NS-74-1**

a)

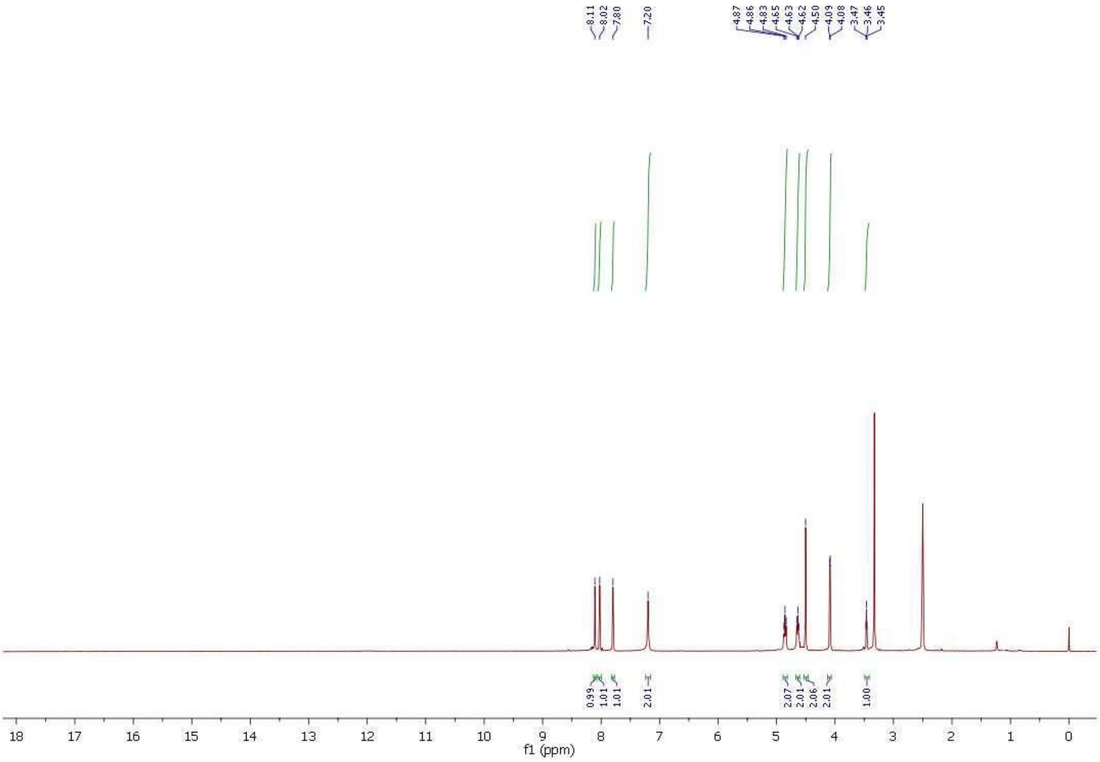

b)

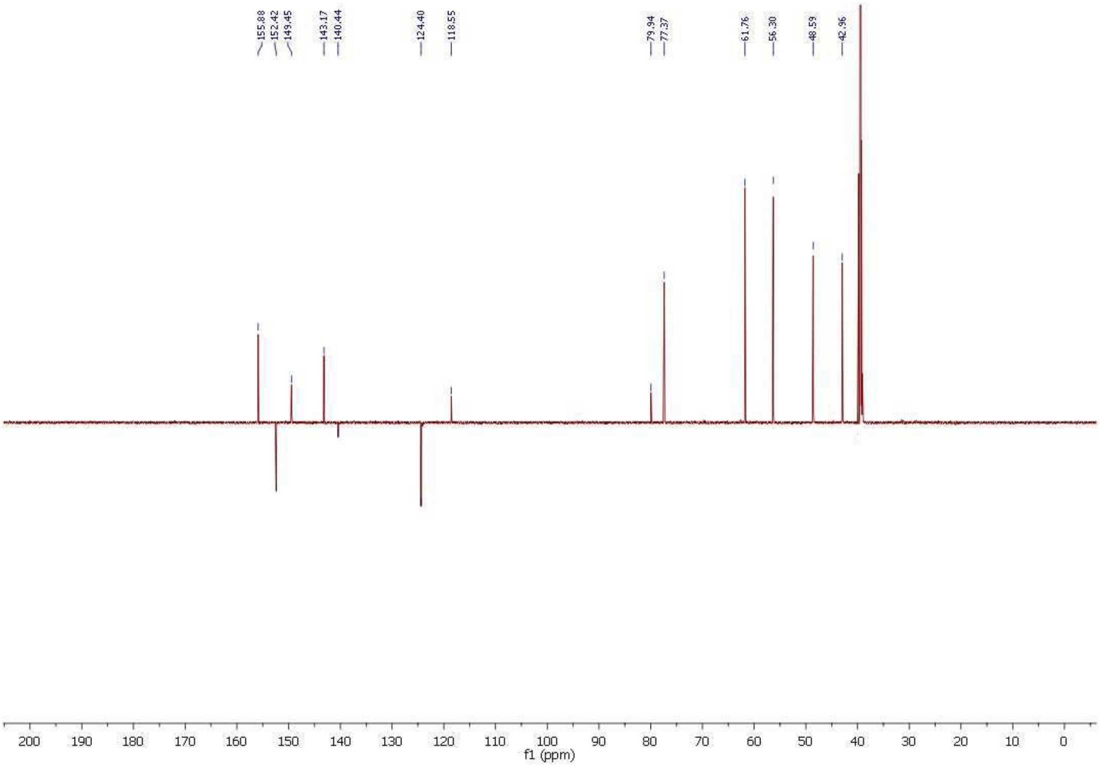

2. Computational analysis

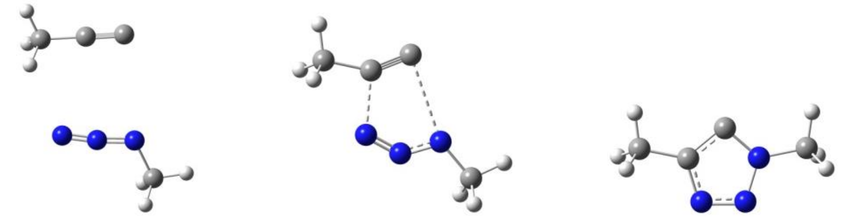

**R**	→	**RC**	→	**TS**	→	**P**
0.0		7.0		25.9		–39.4

Figure S40. Uncatalyzed conversion of the deprotonated alkyne **m1**$^{–}$ and azide **m2** into triazole. Relative Gibbs free energies for isolated reactants (**R**), reactant complex (**RC**), transition state (**TS**) and isolated product (**P**) correspond to the DMF solution (in kcal mol$^{–1}$). The reaction Gibbs free energy is ΔG_{R} = –39.4 kcal mol$^{–1}$, while the kinetic barrier is $\Delta G^{‡}$ = 25.9 kcal mol$^{–1}$ and corresponds to the rate-limiting process denoted in shading.

Metal State	Property	$m1^0$	$m1^-$	$m2^0$
Cu^0	**Structure**	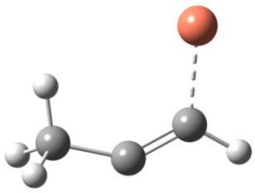	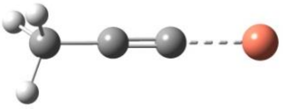	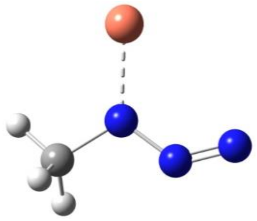
	**Cu–C/N bond**	1.91 Å	1.94 Å	1.90 Å
	**Cu–C/N–C angle**	121.6°	179.8°	123.6°
	**Δ*G*INT**	–11.0 kcal mol^{-1}	–11.5 kcal mol^{-1}	–20.5 kcal mol^{-1}
Cu^+	**Structure**	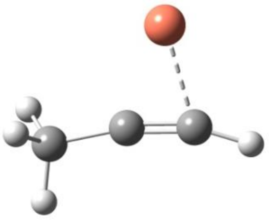	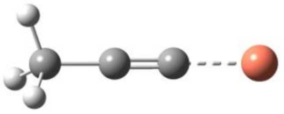	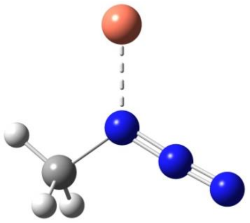
	**Cu–C/N bond**	1.99 Å	1.83 Å	1.95 Å
	**Cu–C/N–C angle**	76.7°	179.9°	123.0°
	**Δ*G*INT**	–11.9 kcal mol^{-1}	–41.4 kcal mol^{-1}	–5.6 kcal mol^{-1}
Cu^{2+}	**Structure**	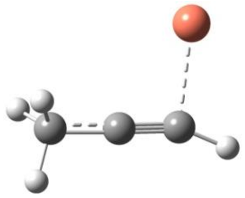	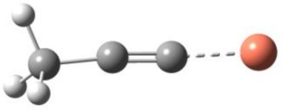	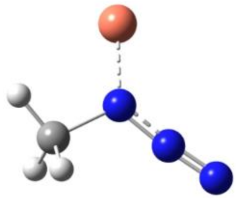
	**Cu–C/N bond**	2.11 Å	1.85 Å	2.05 Å
	**Cu–C/N–C angle**	99.9°	177.3°	118.0°
	**Δ*G*INT**	–13.0 kcal mol^{-1}	–66.6 kcal mol^{-1}	–13.0 kcal mol^{-1}

Figure S41. Structures and relevant geometric parameters for complexes among copper in various oxidation states and the considered neutral alkyne (**$m1^0$**), deprotonated alkyne (**$m1^-$**) and azide (**m2**), together with the calculated interaction Gibbs free energies (ΔG_{INT}). All values correspond to the DMF solution.

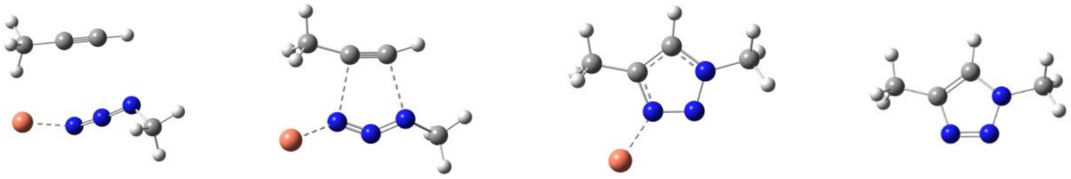

R	→	RC	→	TS	→	PC	→	P
0.0		3.0		18.4		–64.4		–51.0

Figure S42. Cu(I)-catalyzed conversion of alkyne **m1$^{\mathbf{0}}$** and azide **m2** into triazole with Cu(I) bound to azide. Relative Gibbs free energies for isolated reactants (**R**), reactant complex (**RC**), transition state (**TS**), product complex (**PC**) and isolated product (**P**) correspond to the DMF solution (in kcal mol^{-1}). The reaction Gibbs free energy is ΔG_R = –51.0 kcal mol^{-1}, while the kinetic barrier is $\Delta G^‡$ = 18.4 kcal mol^{-1} and corresponds to the rate-limiting process denoted in shading.

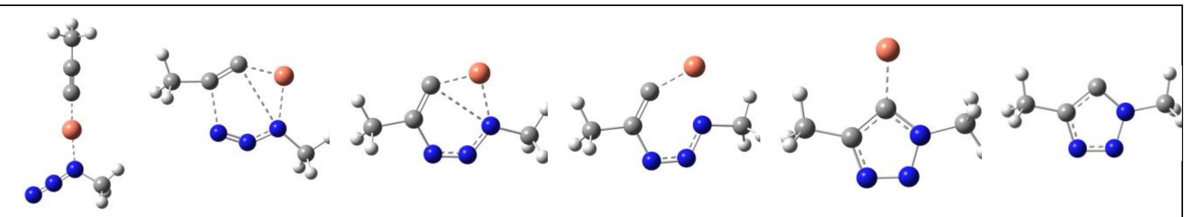

R	RC	TS1	INT	TS2	PC	P
0.0	–42.5	–22.7	–28.7	–24.2	–82.4	–40.3

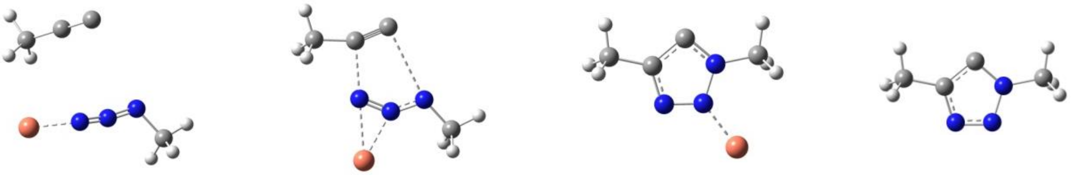

R	→	RC	→	TS	→	PC	→	P
0.0		3.2		22.0		–57.7		–40.3

Figure S43. Cu(I)-catalyzed conversion of deprotonated anionic alkyne **m1**$^{-}$ and azide **m2** into triazole with Cu(I) bound to either alkyne (top) or azide (bottom). Relative Gibbs free energies for isolated reactants (**R**), reactant complex (**RC**), transition state (**TS**), intermediate (**INT**), product complex (**PC**) and isolated product (**P**) correspond to the DMF solution (in kcal mol^{-1}). The reaction Gibbs free energy for both processes is ΔG_{R} = –40.3 kcal mol^{-1}, while the kinetic barriers are $\Delta G^{\ddagger}$ = 19.8 kcal mol^{-1} (top) and $\Delta G^{\ddagger}$ = 22.0 kcal mol^{-1} (bottom), and corresponds to the rate-limiting processes denoted in shading.

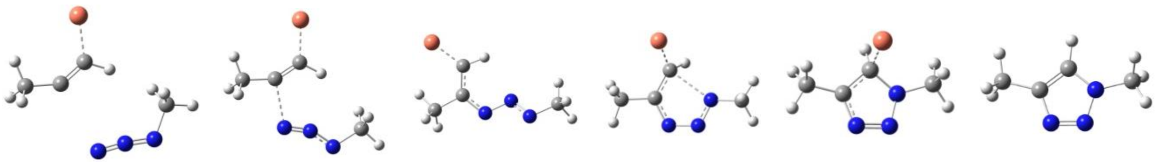

R	RC	TS1	INT	TS2	PC	P
0.0	–1.7	3.4	–25.3	–15.6	–50.1	–51.0

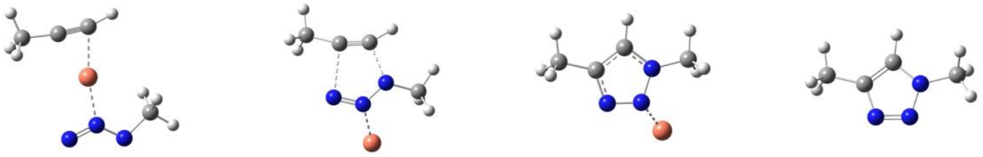

R	→	RC	→	TS	→	PC	→	P
0.0		–13.7		19.3		–48.0		–51.0

Figure S44. Cu(0)-catalyzed conversion of neutral alkyne **m1^{0}** and azide **m2** into triazole with Cu(0) bound to either alkyne (top) or azide (bottom). Relative Gibbs free energies for isolated reactants (**R**), reactant complex (**RC**), transition state (**TS**), intermediate (**INT**), product complex (**PC**) and isolated product (**P**) correspond to the DMF solution (in kcal mol^{-1}). The reaction Gibbs free energy for both processes is ΔG_{R} = –51.0 kcal mol^{-1}, while the kinetic barriers are $\Delta G^{\ddagger}$ = 9.7 kcal mol^{-1} (top) and $\Delta G^{\ddagger}$ = 33.0 kcal mol^{-1} (bottom), and corresponds to the rate-limiting processes denoted in shading.

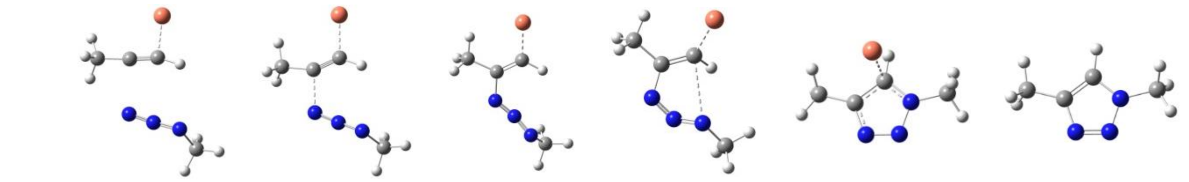

R	RC	TS1	INT	TS2	PC	P
0.0	–9.2	–2.0	–8.9	–0.6	–63.7	–51.0

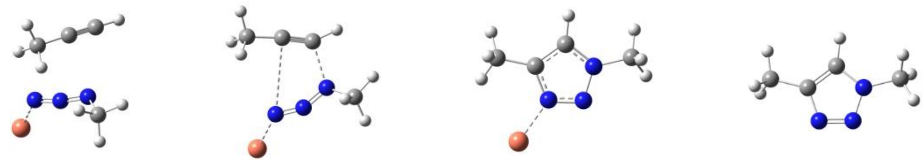

R	→	RC	→	TS	→	PC	→	P
0.0		–3.0		1.3		–78.2		–51.0

Figure S45. Cu(II)-catalyzed conversion of neutral alkyne **m1^{0}** and azide **m2** into triazole with Cu(II) bound to either alkyne (top) or azide (bottom). Relative Gibbs free energies for isolated reactants (**R**), reactant complex (**RC**), transition state (**TS**), product complex (**PC**) and isolated product (**P**) correspond to the DMF solution (in kcal mol^{-1}). The reaction Gibbs free energy for both processes is ΔG_R = –51.0 kcal mol^{-1}, while the kinetic barriers are $\Delta G^‡$ = 8.3 kcal mol^{-1} (top) and $\Delta G^‡$ = 4.3 kcal mol^{-1} (bottom), and corresponds to the rate-limiting processes denoted in shading.

**Model system**	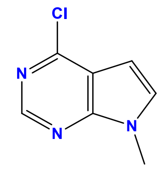	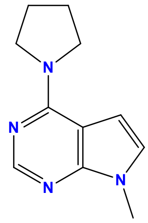	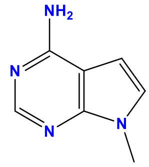
**Most stable complex**	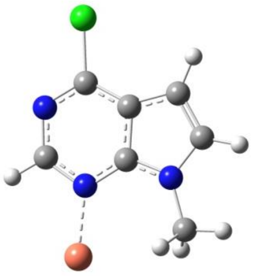	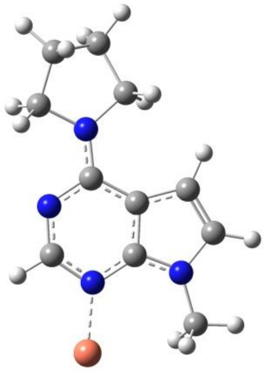	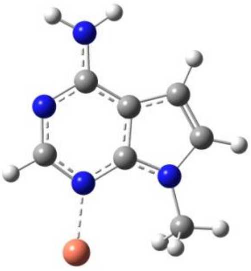
**Cu(I)···N distance**	1.94 Å	1.92 Å	1.93 Å
**Interaction energy Δ*G*$_{\mathbf{INT}}$**	–8.8 kcal mol^{-1}	–12.0 kcal mol^{-1}	–12.8 kcal mol^{-1}

**Model system**	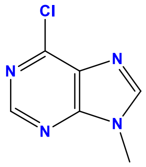	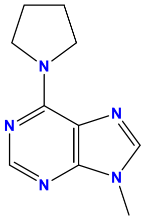	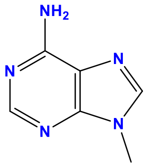
**Most stable complex**	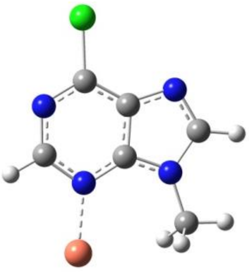	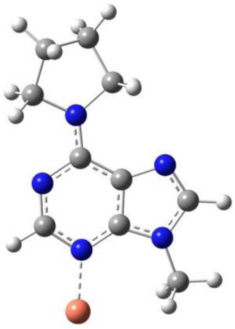	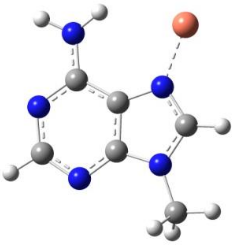
**Cu(I)···N distance**	1.94 Å	1.92 Å	1.91 Å
**Interaction energy Δ*G*$_{\mathbf{INT}}$**	–8.8 kcal mol^{-1}	–12.1 kcal mol^{-1}	–12.3 kcal mol^{-1}

Figure S46. Interaction Gibbs free energies between several model systems and Cu(I) ions together with relevant Cu(I)···N distances.
